# Specific RNA m6A modification sites in bone marrow mesenchymal stem cells from the jawbone marrow of type 2 diabetes patients with dental implant failure

**DOI:** 10.1038/s41368-022-00202-3

**Published:** 2023-01-12

**Authors:** Wanhao Yan, Xiao Lin, Yiqian Ying, Jun Li, Zhipeng Fan

**Affiliations:** 1 Laboratory of Molecular Signaling and Stem Cells Therapy, Beijing Key Laboratory of Tooth Regeneration and Function Reconstruction, Capital Medical University School of Stomatology, Beijing, China; 2 Department of Dental Implant Center, Beijing Stomatological Hospital, School of Stomatology, Capital Medical University, Beijing, China; 3 Department of Oral Implantology, Tianjin Stomatological Hospital, School of Medicine, Nankai University, Tianjin, China; 4 Research Unit of Tooth Development and Regeneration, Chinese Academy of Medical Sciences, Beijing, China

**Keywords:** Mesenchymal stem cells, Type 2 diabetes, Oral diseases

## Abstract

The failure rate of dental implantation in patients with well-controlled type 2 diabetes mellitus (T2DM) is higher than that in non-diabetic patients. This due, in part, to the impaired function of bone marrow mesenchymal stem cells (BMSCs) from the jawbone marrow of T2DM patients (DM-BMSCs), limiting implant osseointegration. RNA N6-methyladenine (m6A) is important for BMSC function and diabetes regulation. However, it remains unclear how to best regulate m6A modifications in DM-BMSCs to enhance function. Based on the “m6A site methylation stoichiometry” of m6A single nucleotide arrays, we identified 834 differential m6A-methylated genes in DM-BMSCs compared with normal-BMSCs (N-BMSCs), including 43 and 790 m6A hypermethylated and hypomethylated genes, respectively, and 1 gene containing hyper- and hypomethylated m6A sites. Differential m6A hypermethylated sites were primarily distributed in the coding sequence, while hypomethylated sites were mainly in the 3′-untranslated region. The largest and smallest proportions of m6A-methylated genes were on chromosome 1 and 21, respectively. MazF-PCR and real-time RT-PCR results for the validation of erythrocyte membrane protein band 4.1 like 3, activity-dependent neuroprotector homeobox (*ADNP*), growth differentiation factor 11 (*GDF11*), and regulator of G protein signalling 2 agree with m6A single nucleotide array results; *ADNP* and *GDF11* mRNA expression decreased in DM-BMSCs. Furthermore, gene ontology and Kyoto Encyclopedia of Genes and Genomes analyses suggested that most of these genes were enriched in metabolic processes. This study reveals the differential m6A sites of DM-BMSCs compared with N-BMSCs and identifies candidate target genes to enhance BMSC function and improve implantation success in T2DM patients.

## Introduction

Diabetes mellitus (DM) is a chronic metabolic disease identified by high blood glucose levels, which can impair the blood vessels, kidneys, heart, eyes, and nerves.^1^ Type 2 DM (T2DM) exceeds 90% of diabetes mellitus case and is characterised by a lack of insulin secretion from pancreatic islet β-cells, insufficient compensatory insulin secretory responses, and tissue insulin resistance (IR).^2^ According to research on both humans and animals, T2DM causes an increase in the susceptibility and severity of periodontal disease, increases inflammatory events in periodontal tissues, impairs the formation of new bone, and increases RANKL expression in response to bacterial challenge. Increased nuclear factor-kappa B (NF-κB) activation and the expression of inflammatory cytokines like tumour necrosis factor-alpha (TNF-α) and interferon-gamma (IFN‐γ) result from these responses, which can ultimately lead to tooth loss.^3^ Currently, the main clinical methods for tooth loss repair include fixed, removable, and implant dentures, and implant-based dental restorations have become the primary treatment option for patients who are missing all or part of their teeth due to advancements in surgical techniques and implant design. Our previous study shows that the failure rate of patients with DM, who have well-controlled glucose before implantation, was 10.77%, while that of non-diabetic patients was 0.75%.^4^ A previous study also reported that insulin-treated T2DM rats had improved bone regeneration and trabecular microstructure to some extent, but not comparable to the control group.^5^ Furthermore, a recent clinical study discovered that T2DM patients with good glycaemic control had lower implant stability during the healing stage than patients without diabetes.^6^ It is thus clear that we need to improve our knowledge regarding the key factors that affect implant failure in patients with diabetes in order to improve the success rate of implantation. In a previous study, the site-specific characteristics of bone marrow mesenchymal stem cells (BMSCs) derived from the iliac crest and jawbone marrow of the same person were described. It was found that BMSCs from the jawbone marrow had a higher proliferation, delayed senescence, and greater osteogenic differentiation ability when compared to those from the iliac crest. Additionally, BMSCs derived from the jawbone marrow have a lower potential for adipogenesis than those derived from the iliac bone marrow, which can reduce fat generation during the regeneration of bone tissue.^7^ After implant surgery, The characteristics of BMSCs from the jawbone marrow may be thought to be advantageous for alveolar bone regeneration after implant surgery. BMSCs start to congregate around the implant surface as soon as the implant is inserted into the jawbone. Following BMSC adhesion to the implant surface, osteogenic differentiation is induced, and new bone is gradually formed with the aid of cells, blood, and associated cytokines.^8^ These findings indicated that BMSCs in the jawbone marrow play a crucial role in implant osseointegration. However, our previous study revealed that the migration, proliferation, and osteogenic differentiation ability of BMSCs derived from the jawbone marrow of T2DM patients (DM-BMSCs) was significantly diminished compared to the BMSCs from normal controls (N-BMSCs).^9,10^ Therefore, enhancing the function of DM-BMSCs and improving the implant success rate are critical for implant surgery.

RNA N6-methyladenine (m6A) is the most prevalent form of methylation modification, which accounts for 0.1%–0.4% of adenosine,^11^ and it is widespread in eukaryotic mRNA. Specifically, m6A methylation refers to the modification of an adenosine to an m6A adenine, which occurs at the N6 site of the adenine base in RNA.^12^ RNA m6A methylation is mainly catalysed by methyltransferase and demethylase, which is similar to DNA methylation;^13^ the methyltransferases, known as “writers”, mainly include methyltransferase like 3 (METTL3), Wilms tumour 1-associated protein (WTAP), and so on. Through demethylases called “erasers”, such as ALKB family member 3 (ALKBH3), ALKBH5, and fat mass and obesity-related protein (FTO), m6A methylation is dynamically reversible and can return the modified RNA to its original RNA. In addition, m6A binding proteins act as “readers”, and these mainly include the YT521-B homology family (YTH), the insulin-like growth factor-2 binding protein family (IGF2BPs), and heterogeneous nuclear ribonucleoprotein families (HNRNP), which recognise m6A modified RNA and regulate mRNA metabolism and function.^14^ M6a participates in almost every process of mRNA metabolism, including RNA transcription, translation, and degradation. Sequencing analysis revealed that most m6A modifications were concentrated in the RRACH motif (R = G/A, H = A/C/U), and within this motif, it was primarily concentrated near the 3′-untranslated region (UTR), followed by the coding sequences (CDS) and 5′-UTR regions.^14^ A previous study found that the m6A level of T2DM patients and diabetic rats was significantly lower compared with the control group, and that T2DM could be characterised by the m6A content. The increased mRNA expression of FTO may be to blame for the reduction of m6A in T2DM, which may further increase the risk of T2DM complications. As potential novel T2DM biomarkers, low m6A levels should be further researched.^15^ A previous study reported that the m6A reader protein YTHDC1, which interacts with SQSTM1 mRNA, was decreased in diabetic keratinocytes during both the acute and long-term effects of hyperglycaemia. The depletion of YTHDC1 enhanced apoptosis rates and impaired wound-healing capacity of diabetic keratinocytes.^16^ The above studies indicate that m6A alterations are closely related to T2DM and may thus be a key target for future prevention and treatment methods. Furthermore, Liu et al. reported that m6A is required for the differentiation of hBMSCs, and that the m6A “reader” YTHDF1 could promote the osteogenesis of BMSCs through the translational control of ZNF839.^17^ Li et al. found that the osteogenic differentiation of BMSCs is negatively regulated by the m6A demethylase ALKBH5 via PRMT6.^18^ This above indicates that different biological functions can be produced by m6A modifications in BMSCs, and that m6A alterations can regulate BMSC function. In addition, in mice with diabetic foot ulcers (DFUs), adipose-derived mesenchymal stem cells (ADSCs) were found to promote lymphangiogenesis through the METTL3 pathway and improve wound healing by regulating VEGF-C through the METTL3/IGF2BP2-m6A pathway.^19^ However, how to regulate m6A modifications in DM-BMSCs to enhance impaired function and improve the implant success rate is currently unclear.

In this study, we used an m6A single nucleotide array to analyse the changes in the m6A sites of BMSCs in well-controlled T2DM patients and non-diabetic patients. To identify the potential biological functions of the target genes, bioinformatics analysis including Gene Ontology (GO) and Kyoto Encyclopedia of Genes and Genomes (KEGG) pathway enrichment analyses were also carried out.

## Results

### Differential m6A sites and differentially m6A-methylated genes

**Fig. 1:**
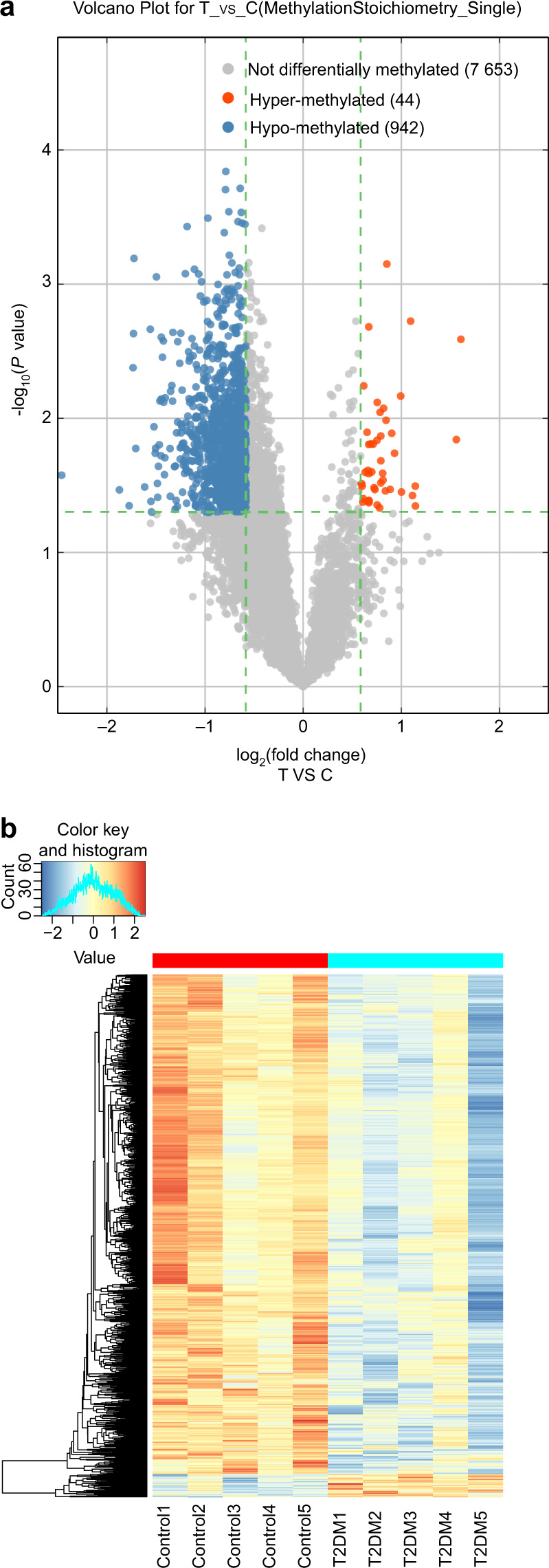
Differential m6A-methylated sites in the T2DM and control groups. **a** The differential m6A-methylated sites between the T2DM and control groups are shown in a volcano plot representation of the microarray data. The blue and red dots to the left and right of the two vertical lines denote statistically significant differential m6A-methylated sites and show a ≥ 1.5-fold change. **b** differential m6A-methylated sites between the T2DM and control groups were analysed using hierarchical clustering. The differential m6A-methylated sites finally aggregated into two main branches, including the T2DM group’s m6A hypermethylated (labelled in red) and hypomethylated (labelled in blue) sites, according to hierarchical clustering. The contrast is more pronounced the darker the colour

Using the filtering criteria of |FC| ≥ 1.5 and a P-value < 0.05, based on “m6A site methylation stoichiometry”, 986 differential m6A-methylated sites were identified in the BMSCs of the T2DM group when compared with the control group; of which 44 were m6A hypermethylated sites (Table S1; including the 3’UTR of *EPB41L3*, CDS of *YTHDC1*, 3’UTR of *NAA60*, CDS of *DIDO1*, and 3’UTR of *MAFK*) and 942 were m6A hypomethylated sites (Table S2; including the 3’UTR of *ADNP*, CDS of *YTHDF3*, CDS of *YTHDF2*, CDS of *GDF11*, and 3’UTR of *RGS2*) (Fig. 1a), and hierarchical clustering was done based on all notable m6A-methylated sites to speculate on the relationship between samples (Fig. 1b). In addition, 834 differential m6A-methylated genes in the BMSCs were identified in the T2DM group when compared with the control; of which 43 were m6A hypermethylated genes (including those in *ZNF12, MAFK, NCOA7, EPB41L3*, and *TIFA*) and 790 were m6A hypomethylated genes (including those in *TM4SF1*, *PRPSAP1*, *OTUD5*, *GTPBP4*, and *TAF3*). *PRRC2C* has both hyper- and hypomethylated m6A sites.

Similarly, using the filtering criteria of |FC| ≥ 1.5 and a P-value < 0.05, based on “m6A site abundance”, 800 differential m6A-methylated sites in the BMSCs were identified in the T2DM group when compared with the control; of which 44 were m6A hypermethylated sites (Table S3; including the 3’UTR of *SPEN*, CDS of *INF2*, CDS of *ZFYVE19*, CDS of *ZNF394*, and 3’UTR of *BORCS8*) and 756 were m6A hypomethylated sites (Table S4; including the 3’UTR of *METTL14*, 3’UTR of *YTHDC2*, CDS of *YTHDF2*, CDS of *TRMT61B*, and 3’UTR of *RARRES3*). In addition, 710 differential m6A-methylated genes in BMSCs were identified in the T2DM group when compared with the control using the array analysis; of which 38 were m6A hypermethylated genes (including *LRIG3*, *BICD2*, *KLF12*, *ZFYVE19*, and *RPUSD4*) and 666 were m6A hypomethylated genes (including *LURAP1L*, *TRIB3*, *RCAN1*, *PRPSAP1*, and *RRAGC*). Furthermore, 6 genes were identified as having both hyper- and hypomethylated m6A sites (*TRIAP1*, *ZNF623*, *FOXK2*, *PDE4B*, *SPEN*, and *GCC1*).

**Fig. 2:**
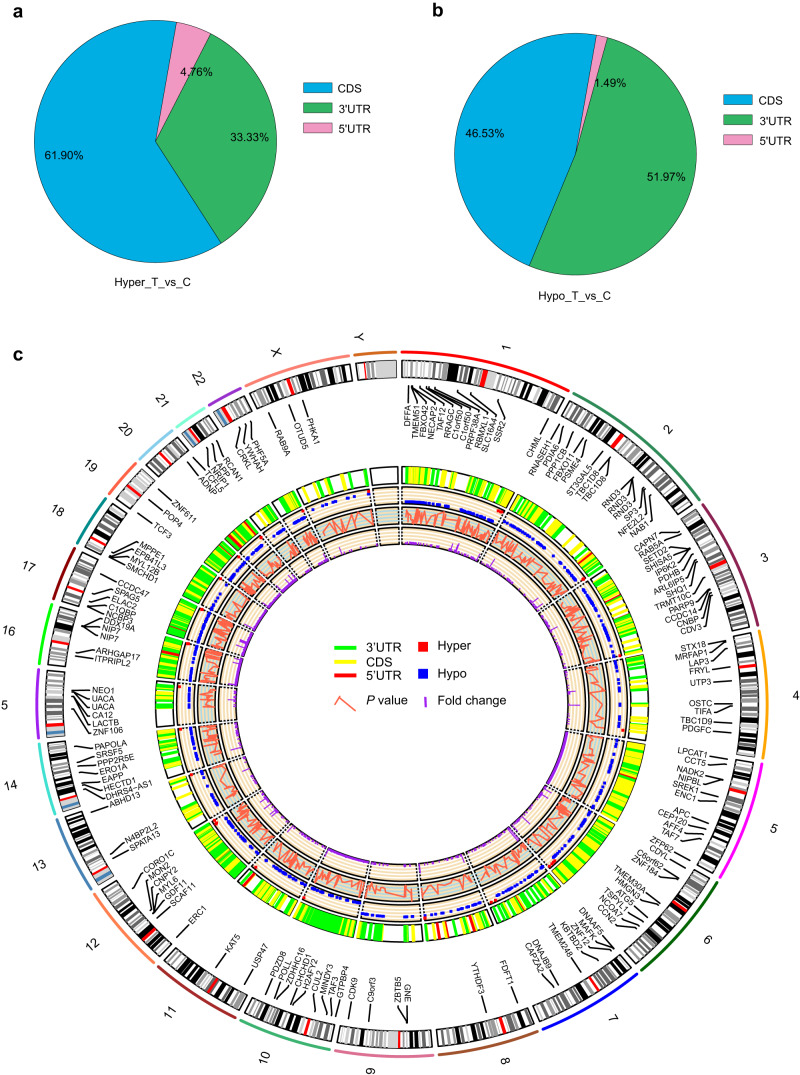
Distribution of differential m6A-methylated sites and a chromosome circos diagram of the differentially m6A-methylated genes in the T2DM and control groups. Sector graphs showing the distribution of m6A (**a**) hypermethylation and (**b**) hypomethylation in the indicated regions of the T2DM group. **c** Chromosome circos diagram showing the differentially m6A-methylated genes per chromosome in the T2DM and control groups

We then analysed the proportion of m6A methylation sites in different gene structures based on the “m6A site methylation stoichiometry”. The differentially m6A hypermethylated sites were mainly distributed in the CDS (61.90%; including *NCOA7*, *TIFA*, *SPAG5*, *BIRC6*, and *FAM83H*), followed by the 3ʹ-UTR (33.33%; including *ZNF12*, *MAFK*, *EPB41L3*, *ZBTB37*, and *SMIM13*), and the 5ʹ-UTR (4.76%; including *PCED1A* and *MAGI1*; Fig. 2a). The differentially m6A hypomethylated sites were distributed with similar percentages in the 3ʹ-UTR (51.97%; including *TM4SF1*, *PRPSAP1*, *OTUD5*, *GTPBP4*, and *PCYT1A*) and the CDS (46.53%; including *TAF3*, *CDYL*, *C1orf50*, *CCDC14*, and *CORO1C*), with the fewest located in the 5ʹ-UTR (1.49%; including *CNPY2*, *DDX6*, *EPN2*, *SLC20A2*, and *HSBP1*; Fig. 2b). Additionally, we analysed the distribution of genes with differential m6A-methylated sites in the chromosomes (Fig. 2c). The chromosome circos diagram showed that there were multiple differentially m6A-methylated genes on most chromosomes. The largest amount was 4 m6A hyper-regulated genes and 96 m6A hypo-regulated genes on chromosome 1, and the smallest amount was 12 m6A hypo-regulated genes and no m6A hyper-regulated genes on chromosome 21, and both were in the T2DM group. There were no m6A hyper- and hypo-regulated genes on chromosome Y (Fig. 2c).

### MazF-polymerase chain reaction and real-time reverse transcriptase-polymerase chain reaction of genes with differential m6A modifications and mRNA expression

**Fig. 3:**
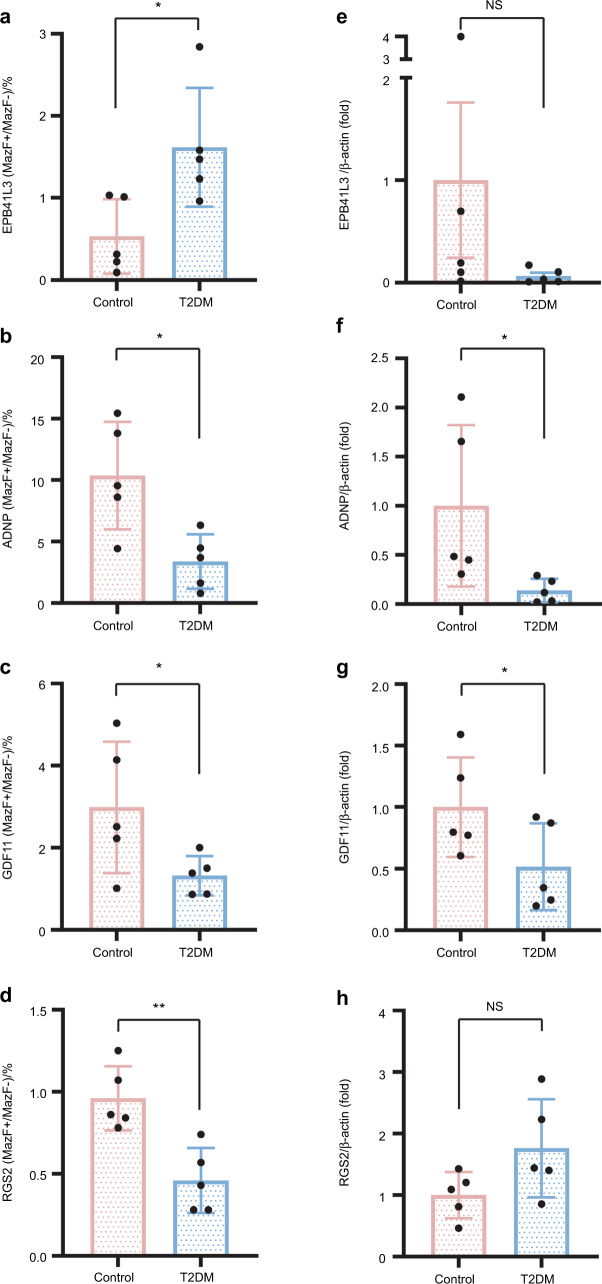
MazF-PCR and real-time RT-PCR verification of genes with distinct m6A alterations and varied mRNA expression. **a-d** m6A methylation levels of the BMSCs were measured using MazF-PCR in the T2DM and control groups. m6A methylation levels for (**a**) *EPB41L3*, (**b**) *ADNP*, (**c**) *GDF11*, and (**d**) *RGS2*. **e-h** mRNA expression levels of BMSCs in the T2DM and control groups were measured using real-time RT-PCR. mRNA expression levels for (**e**) *EPB41L3*, (**f**) *ADNP*, (**g**) *GDF11*, and (**h**) *RGS2*. Internal control was provided by β-actin. To evaluate statistical significance, the student’s t-test was used. SD (*n* = 3) is shown by error bars. *P ≤ 0.05; **P ≤ 0.01

To further validate the m6A single nucleotide array results, MazF-polymerase chain reaction (MazF-PCR) and real-time reverse transcriptase-polymerase chain reaction (RT-PCR) were used to verify the m6A methylation and mRNA levels for *EPB41L3*, *ADNP*, *GDF11*, and *RGS2*. The m6A methylation level for *EPB41L3* was significantly upregulate in the T2DM group when compared with the control group, in accordance with the m6A single nucleotide array results (Fig. 3a), whereas there was no significant difference in mRNA levels (Fig. 3e). On the contrary, the m6A methylation levels of *ADNP*, *GDF11*, and *RGS2* were significantly downregulated in the T2DM group when compared with the control group (Fig. 3b–d), and the mRNA levels of the *ADNP* and *GDF11* were decreased in accordance with the m6A single nucleotide array results (Fig. 3f, g), and there was no significant difference in the mRNA levels of the *RGS2* (Fig. 3h).

### Protein–protein interactions of the differentially m6A-methylated genes

**Fig. 4:**
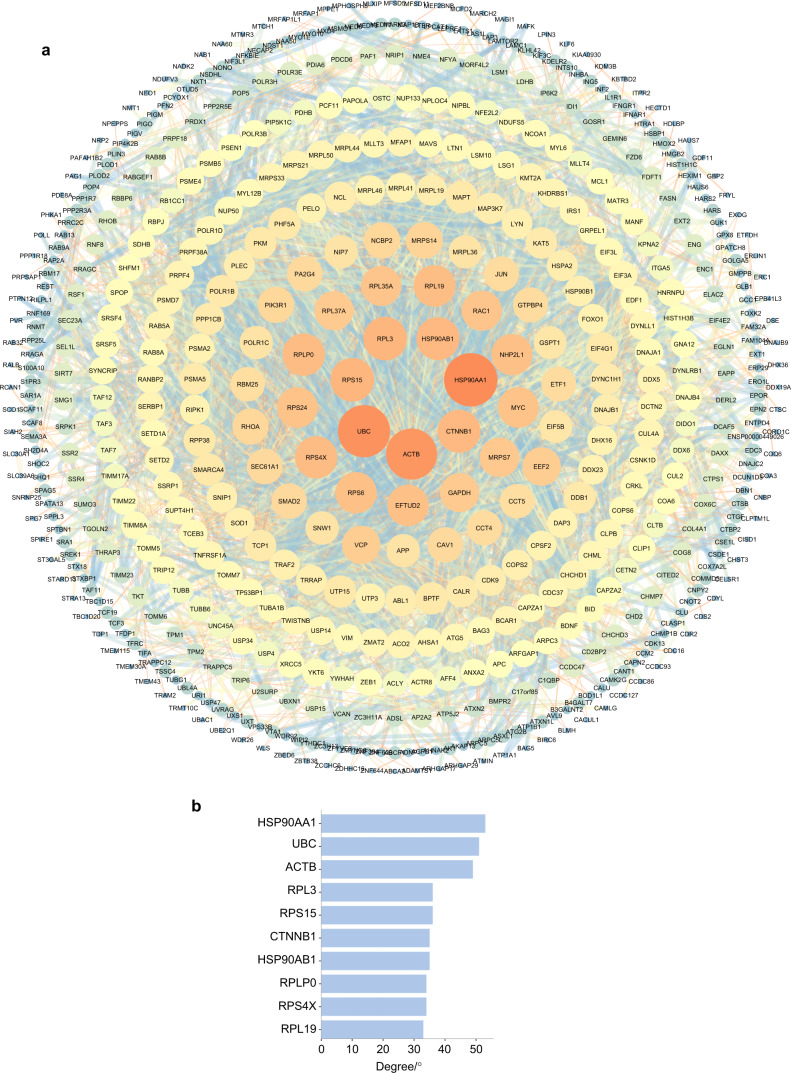
PPI network of differentially m6A-methylated genes. **a** Differentially m6A methylated genes with interactions and a protein–protein interaction network diagram was created using combined scores >0.7. **b** Top ten hub genes with a higher degree of connectivity

Based on “m6A site methylation stoichiometry” and interactions with combined scores ≥0.7,^20^ using the STRING online database and Cytoscape software, a total of 564 differentially m6A-methylated genes of the 834 commonly altered differentially m6A-methylated genes were filtered into the protein–protein interaction **(**PPI) network complex, consisting of 564 nodes and 3684 edges (Fig. 4a). Information on the differentially m6A-methylated genes in the PPI network is shown in Table S5. The top ten hub genes were *HSP90AA1*, *UBC*, *ACTB*, *RPL3*, *RPS15*, *CTNNB1*, *HSP90AB1*, *RPLP0*, *RPS4X*, and *RPL19*, and they were identified using the cytoHubba plug-in as having a higher degree of connectivity (Fig. 4b).

### Functional enrichment analysis of the differentially m6A-methylated genes

**Fig. 5:**
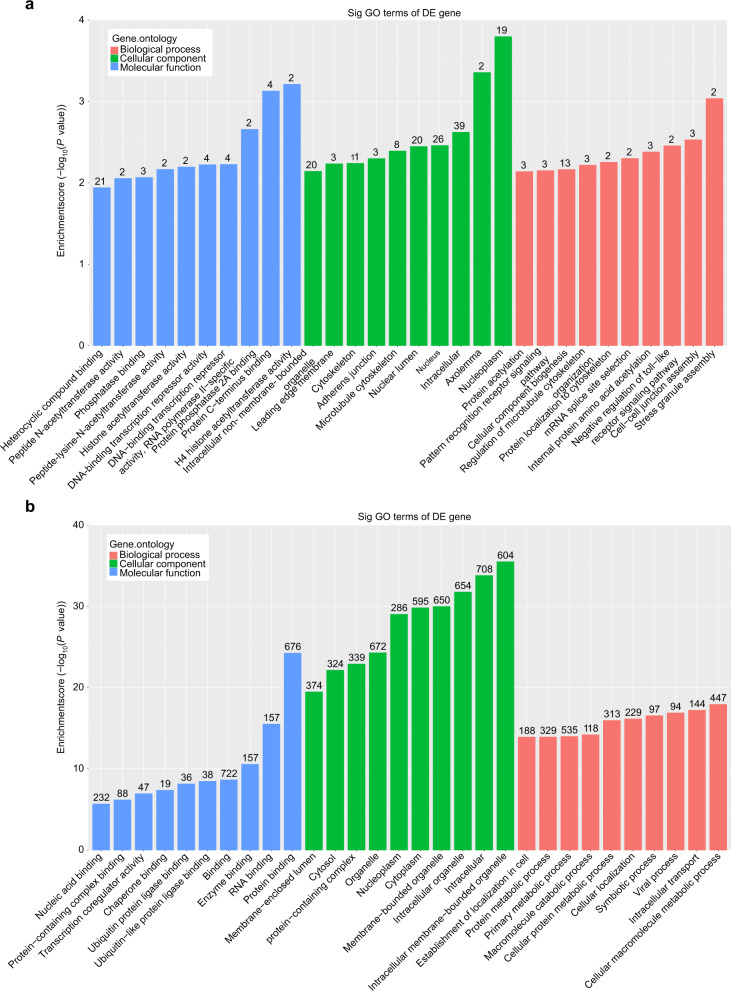
GO analyses of the differentially m6A-methylated genes. Top 10 enriched items obtained from the GO analyses of the differentially expressed m6A (**a**) hypermethylated and (**b**) hypomethylated genes

Based on “m6A site methylation stoichiometry”, the GO analysis results show a significant level of m6A-methylation for the upregulated genes with differential expression in the T2DM group, which were enriched in 188 GO terms. These included 125 GO terms related to biological processes, mainly distributed in cellular component biogenesis, cell-cell junction assembly, and the internal protein amino acid acetylation pathway (Fig. 5a); 32 terms related to cellular components which participate in the intracellular, nucleus, and nuclear lumen pathways (Fig. 5a); and 31 terms related to molecular function categories, mainly heterocyclic compound binding, protein C-terminus binding, DNA-binding transcription repressor activity, and the RNA polymerase II-specific pathway (Fig. 5a). Similarly, significant m6A-methylated downregulated genes with differential expression in the T2DM group were enriched in 1605 GO terms. These included 1205 GO terms related to biological processes, mainly distributed in primary metabolic processes, cellular macromolecule metabolic processes, and protein metabolic process pathways (Fig. 5b); 230 terms related to cellular components which are involved with intracellular, organelles and intracellular organelle pathways (Fig. 5b); and 170 terms related to molecular function categories, mainly binding, protein binding, and nucleic acid binding pathways (Fig. 5b).

**Fig. 6:**
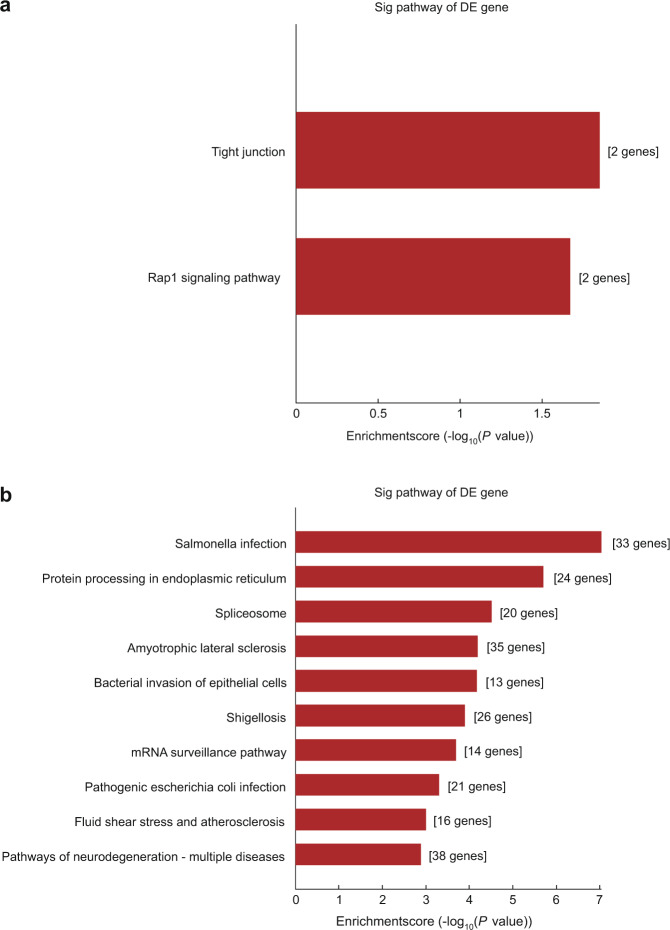
KEGG analyses of the differentially m6A-methylated genes. The first ten enriched pathways identified in the KEGG analysis of the differentially expressed m6A (**a**) hypermethylated and (**b**) hypomethylated genes

The KEGG results show that the main pathways enriched for the m6A-methylated upregulated genes involved tight junctions and the Rap1 signalling pathway (Fig. 6a), while neurodegeneration, amyotrophic lateral sclerosis, and salmonella infection pathways were mainly enriched among the top ten pathways based on the m6A-methylated downregulated genes (Fig. 6b).

## Discussion

In this study, we selected two groups of five BMSCs from implant failure well-controlled T2DM patients and non-diabetic patients and used m6A single nucleotide arrays to investigate the hub mRNAs and the changes in the m6A sites of the BMSCs. On average, m6A modifications on each mRNA occurred in the order of three.^21^ While most mRNAs have only one m6A site, some can carry >20 m6A modifications.^22^ If there were no other m6A sites upstream or downstream of a particular site, it was defined as an ‘m6A single nucleotide site’.^23^ Studies have shown that m6A single-nucleotide sites are involved in molecular functions and processes, such as mRNA translation, initiation, and elongation. These sites also regulate non-coding RNA activity and degradation.^24^ In addition, the present study revealed that m6A single-nucleotide sites have significant functions at the molecular, cellular, and organismal levels.^25–27^ In summary, m6A single-nucleotide modifications are closely associated with the occurrence and progression of numerous diseases. Furthermore, m6A modification reportedly impacts BMSC proliferation, differentiation, and apoptosis by regulating the expression of *ALP, RUNX2*, *OSX*, *VEGF*, and other related genes.^28^ M6A also regulates SOX9 translation during the chondrogenic differentiation of BMSCs, and, thus, represents a potential therapeutic target for repair of cartilage defects.^29^ Moreover, METTL3 knockdown decreases the osteogenic differentiation ability of BMSCs via the PI3K-Akt signalling pathway.^30^ Taken together, these findings indicate that RNA m6A methylation could regulate the fate of BMSCs. In our study, based on “m6A site methylation stoichiometry”, our finding found the DM-BMSCs had 986 differentially m6A-methylated sites and 834 differentially m6A-methylated genes when compared with the N-BMSCs. Analysis of the differential m6A site methylation stoichiometry genes in the DM-BMSCs and N-BMSCs groups, showed that hypomethylated genes were much more abundant than hypermethylated genes in the T2DM group. Our results showed that the RNA m6A readers, YTHDF2 and YTHDF3, were hypomethylated in DM-BMSCs. Zheng et al. found that YTHDF2 was a crucial gene in the emergence of T2DM, and it could thus be used as a biomarker and therapeutic target.^31^ In addition, the increased adipogenic differentiation ability of METTL3-depleted porcine BMSCs is partially inhibited by the overexpression of YTHDF2.^32^ Furthermore, YTHDF3 levels were also found to be upregulated in the placentas of gestational diabetes mellitus (GDM), showing excellent classifying power for the GDM and control groups.^33^ These findings indicate a critical role for the m6A readers YTHDF2 and YTHDF3 in the occurrence, progression, and prevention of DM, and could thus help to restore the damaged functions of DM-BMSCs to improve the success rate of implantation. However, the role of YTHDF2 and YTHDF3 in DM-BMSCs and their effects on the implant success rate require further exploration.

Moreover, analysis of the differential m6A sites in the T2DM and control groups, showed that the hyper- and hypo-regulated m6A sites were not the same, among which the differentially m6A hypermethylated sites were mostly concentrated in the CDS, while m6A hypomethylated sites were mainly concentrated in the 3ʹ-UTR. In addition, the results showed that the differentially hypermethylated genes were mainly located on chromosomes 1, 6, 16, 17, and 20, whereas the differentially hypomethylated genes were mainly located on chromosome 1. The most hypermethylated gene, *ZNF12* was located on chromosome 7, and the most hypomethylated gene, *TM4SF1* was located on chromosome 3. In summary, chromosomes 1 has the largest number of differentially m6A-methylated genes, as well as some osteogenesis differentiation related and m6A related genes, such as *YTHDF2* and *RGS2*, indicating that it should be the focus of further research.

According to their biological functions, we selected *EPB41L3*, *ADNP*, *GDF11*, and *RGS2* for further analysis. The MazF-PCR results showed that the m6A methylation level of *EPB41L3* was significantly higher in the T2DM group when compared with the control group accord with m6A single nucleotide array results, while the real-time RT-PCR results revealed that there was no discernible difference in mRNA levels. The m6A methylation levels of *ADNP*, *GDF11*, and *RGS2* were significantly downregulated in the T2DM group when compared with those in the control group, while the mRNA levels of *ADNP* and *GDF11* were decreased, and there was no discernible difference in the mRNA levels of *RGS2*. M6a is involved in almost all mRNA metabolism-related processes, including its regulation, transcription, maturation, translation, degradation, and stability.^34^ When m6A sites simply affect the translational efficiency, but not the stability of the mRNA, mRNA levels can remain unchanged, and m6A sites on *EPB41L3* and *RGS2* may precisely do so. Thus, there were no discernible changes in the mRNA levels of *EPB41L3* and *RGS2*. Erythrocyte membrane protein band 4.1 like 3 (EPB41L3) has been approved as a biomarker to identify high-grade intraepithelial lesions and cancers in the cervical region, and for distinguishing between these lesions and those that are most likely to clear.^35^ Likewise, it has been demonstrated that there is a strong correlation between the degree of EPB41L3 methylation and anal disease.^36^ EPB41L3 methylation was significantly higher in oropharyngeal cancer (OPC), which can maximise specificity and sensitivity for early OPC detection, regardless of the stage of early vs. late disease.^37^ In conclusion, these results show that EPB41L3 is closely associated with the occurrence and progression of cancer. Activity-dependent neuroprotector homeobox (ADNP), a transcription factor and cytoskeletal-binding protein, plays a crucial role in cellular growth and proliferation.^38^ Wound healing experiments have shown that ADNP promotes cell migration.^39^ NAP, a brief peptide with eight amino acids derived from ADNP, acts as a protective agent against cerebral ischaemia, central nervous system complications of DM, and retinal damage induced by different insults.^40–42^ A previous study found that NAP inhibits hyperglycaemia/hypoxia-induced retinal pigment epithelium barrier breakdown via modulating HIFs and VEGF production.^43^ Additionally, it also has antioxidant properties, metal-chelating, prevents ROS formation, anti-apoptotic activity, and anti-inflammatory properties.^43–46^ As a member of the transforming growth factor-β (TGF-β) superfamily, growth differentiation factor 11 (GDF11) was first identified in preodontoblasts at the late cap stage.^47^ A portion of the human pulp tissue, particularly the odontoblast layer, which is the outermost pulp layer, showed GDF11-positive staining.^48^ Nakashima et al. showed that exogenous GDF11 gene delivery could positively upregulate the odontogenic differentiation of dental pulp stem cells (DPSCs).^49–51^ Qi et al. reported that endogenous GDF11 could enhance the odontogenic differentiation of DPSCs,^48^ indicating that GDF11 plays a crucial role in MSC differentiation. Moreover, GDF11 attenuated the progression of T2DM by enhancing islet β-cell function and survival.^52^ Recombinant GDF11 (rGDF11) has been reported to reduce body weight and improve glucose homeostasis in mice,^53^ suggesting that it also regulates diabetes. In addition, GDF11 has anti-inflammatory and antioxidant properties and inhibits cell apoptosis and anti-ageing properties.^54–57^ Moreover, the GDF11-FTO-PPARγ axis prompted the shift of BMSC commitment to adipocyte and inhibited bone formation during osteoporosis, as a result of the imbalance between bone mass and fat, indicating that m6A “eraser” can affect the function of GDF11 in BMSCs.^58^ These results indicate that GDF11 may be an important target gene by which to improve the implant success rate in patients with T2DM. Regulator of G protein signalling 2 (RGS2) belongs to the B/R4 subfamily of the RGS protein family and is reportedly expressed in osteoblasts and induced by the cAMP-PKA pathway.^59^ Studies have shown that RGS2 is highly upregulated in human dental follicle cells (hDFCs) and in hADSCs undergoing osteogenic differentiation.^60,61^ RGS2 was also reported to be expressed in rat metaphyseal and diaphyseal bones, as well as in cultured mouse osteoblasts, suggesting that it may play a role in bone development.^62^ In summary, previous studies reported that the function of DM-BMSCs was impaired,^9,10^ the data indicates the m6A hypomethylation levels of *ADNP* and *GDF11* may be related to the mRNA levels, then further influence the impaired BMSC function of patients with T2DM. This may be because they are associated with the osteogenic differentiation and proliferation of BMSCs around implants, the promotion of implant osseointegration and regulation of alveolar bone remodelling after implantation surgery, and they may have anti-inflammatory, antioxidant, and protective roles. Therefore, we may increase the level of *ADNP* and *GDF11* m6A methylation modification in DM-BMSCs and, subsequently, restoring the impaired BMSC function in T2DM patients, then influence the implant success rate of patients with T2DM.

Furthermore, the differentially expressed m6A methylated genes were analysed using GO and KEGG methods. Among the GO terms enriched in hypomethylated genes of DM-BMSCs, biological processes and pathways were associated with primary metabolic processes, cellular macromolecule metabolic processes, and protein metabolic process pathways. Previous studies have shown that IR sequelae include chronic inflammation, oxidative stress status imbalance, and the occurrence of metabolic syndrome.^63^ Aguilar-Recarte et al. reported that metformin lowers glucose through by inhibiting mitochondrial respiratory chain complex 1 and activating AMP-activated protein kinase (AMPK), which has been recognised as a potential IR-related pathway.^64^ Similarly, Entezari et al. also showed that AMPK signalling improves insulin sensitivity and prevents oxidative stress and cell death in cells. Entezari et al. also showed that AMPK signalling improves insulin sensitivity and prevents oxidative stress and cell death in β cells.^65^ These results suggest that metabolic processes play an crucial role in the occurrence, development, and treatment of T2DM. In addition, mitochondrial dysfunction and the oxidative stress in osteoblasts at the titanium-bone interface (TBI) are both important factors in diabetes-induced poor bone repair and implant destabilization, and thus might be therapeutic targets. Adiponectin, a cytokine, could also improve the osteointegration of implants by rescuing mitochondrial impaired through the AMPK pathway under diabetic conditions, both in vivo and in vitro.^5,66^ Selenomethionine, a naturally occurring amino acid-containing selenium, could attenuate H_2_O_2_-induced suppression of the osteogenic differentiation of BMSCs through an antioxidant effect that was modulated by PTEN/PI3K/AKT pathway, which could be a promising antioxidant candidate by which to reduce oxidative stress during dental implant osteointegration process.^67^ Our study, in combination with previous studies, showed that *ADNP* and *GDF11* may affect the metabolic process and antioxidant functions of DM-BMSCs, which could thus be a crucial target by which to address implant stability and bone integration in patients with T2DM.

In conclusion, this study has identified the differential m6A sites and m6A-methylated genes of BMSCs when comparing T2DM and non-diabetic groups. The results reveal the potential link between the RNA m6A-methylated modifications and impaired BMSC function, as a result of T2DM. Furthermore, our study indicated that *ADNP* and *GDF11* m6A methylation levels may be closely related to impaired BMSC function in T2DM patients and could thus be utilised as targets in the future to enhance function and improve the success of implantation in patients with T2DM.

## Materials and methods

### Subject enrolment and ethics statement

With informed patient permission, the Ethics Committee of Beijing Stomatological Hospital, Capital Medical University allowed this study (approval no. CMUSH-IRB-KJ-PJ-2018-08). All subjects recruited and the glycaemic control criteria for T2DM in this investigation were also in accordance with our previous study.^4^ Five patients who met the glycaemic control standard, were ready for implant surgery, but whose implant failed during the healing period, were included in the study (T2DM group). The ratio of healthy subjects (control group) to the T2DM group was 1:1. All basic information, including age (T2DM group: 54.2 ± 8.1 years old; control group: 54.6 ± 7.7 years old), sex (male), general health status, implant system, and implant location, were matched to minimise the impact of external factors. The other specific patient inclusion criteria were the same as previously described.^4^

### Implant insert, bone chip extraction, and cell culture

The same surgeon performed all of the implantation surgeries in this study. The implants were placed as described in our previous study.^68^ The collection and resuspension of bone chips, and BMSC culture and identification were also carried out as previously described.^4,69^

### m6A single nucleotide array analysis

Total RNA from 10 BMSC samples, including five T2DM samples and five control group samples, was extracted using Trizol (CWBIO, Taizhou, China). RNA concentrations were measured using a NanoDrop ND-1000 (Thermo Fisher Science, Walsham, Massachusetts, USA). Arraystar standard protocols were followed for sample preparation and microarray hybridisation. To summarise, total RNA was divided into two fractions. One fraction denoted as “MazF-digested” was treated with RNA endoribonuclease MazF (Takara, Shiga, Japan) to cleave the unmodified m6A sites; the other fraction denoted as “MazF-undigested” was not treated with MazF at either the modified or unmodified sites. The Arraystar Super RNA Labeling Kit (Arraystar, Rockville, MD, USA) was used to label the “MazF-digested” and “MazF-undigested” RNAs with Cy5 and Cy3 as cRNAs separately. Then, The Arraystar Human m6A Single Nucleotide Array (8 × 15 K, Arraystar) was used to combine and hybridise the “MazF-digested” and “MazF-undigested” cRNAs. After washing the slides, the arrays were scanned in two-colour channels by using an Agilent Scanner G2505C (Agilent, Santa Clara, CA, USA).

The acquired array images were analysed by Agilent Feature Extraction software (version 11.0.1.1). The average for the log_2_-scaled spike-in RNA intensities was used to normalise the raw intensities of the MazF-digested (Cy5-labelled) and MazF-undigested (Cy3-labelled) samples. Then, probe signals with present (P) or marginal (M) QC flags were retained for “m6A site methylation stoichiometry” and “m6A site abundance” analyses. The “m6A site methylation stoichiometry” was computed based on the percentage of modification at each site, “m6A site abundance” was computed based on the m6A site methylation amount. The fold change (FC) and P-value thresholds were used to compile the differentially m6A-methylated sites between DM-BMSCs and N-BMSCs. The m6A-methylation pattern was visualised using hierarchical clustering among samples.

### Differential m6A-methylated site and differential m6A site abundance analysis

To compare the differential m6A modifications between the T2DM and control groups, FC was determined for each probe. The default thresholds were an |FC| ≥ 1.5 and P-value < 0.05.

#### Differential “m6A site methylation stoichiometry” profiling

The differential “m6A site methylation stoichiometry” for an m6A site is the percentage modified for the specified site (% modified), and this is calculated based on the normalised intensities of the MazF-digested RNA (Cy5-channel), the MazF-undigested RNA (Cy3-channel), and the correction factor (ratio of MazF-undigested sample amount to MazF-digested sample amount):

1 $${{{\mathrm{\% \,modified}}}} = \frac{{{{{\mathrm{modified}}}}\;{{{\mathrm{site}}}}}}{{{{{\mathrm{total}}}}\;{{{\mathrm{site}}}}}} = \frac{{{{{\mathrm{Digested}}}}}}{{{{{\mathrm{Undigested}}}}}} = \frac{{{{{\mathrm{Cy}}}}5\;{{{\mathrm{normalised}}}}\;{{{\mathrm{intensity}}}}}}{{{{{\mathrm{Cy}}}}3\;{{{\mathrm{normalised}}}}\;{{{\mathrm{intensity}}}}}} \ast {{{\mathrm{Correction}}}}\;{{{\mathrm{factor}}}}$$

The raw intensities for the MazF-digested (Cy5-labelled) and MazF-undigested (Cy3-labelled) were normalised using the average for the log_2_-scaled spike-in RNA intensities:

2 $$\begin{array}{l}{{{{\log}}}}_2\left( {{\rm{Digested}}_{{\rm{Cy}}5\;{\rm{normalized}}\;{\rm{intensity}}}} \right) = \\ {{{{\log}}}}_2\left( {{\rm{Digested}}_{{\rm{Cy}}5\;{\rm{raw}}}} \right) - {{{\mathrm{Average}}}}\left[ {{{{{\log}}}}_2\left( {{\rm{Digested}}_{{\rm{spike}} - {\rm{in}}\_{\rm{Cy5}}\;{\rm{raw}}}} \right)} \right]\end{array}$$

3 $$\begin{array}{l}{{{{\log}}}}_2\left( {{\rm{Undigested}}_{{\rm{Cy}}3\;{\rm{normalized}}\;{\rm{intensity}}}} \right) = {{{{\log}}}}_2\left( {{\rm{Undigested}}_{{\rm{Cy}}3\;{\rm{raw}}}} \right)\\ - {{{\mathrm{Average}}}}\left[ {{{{{\log}}}}_2\left( {{\rm{Undigested}}_{{\rm{spike}} - {\rm{in}}\_{\rm{Cy}}3\;{\rm{raw}}}} \right)} \right]\end{array}$$

The correction factor was the ratio of the RNA sample amount used for MazF-undigestion (undigested sample amount) to the RNA sample amount used for MazF-digestion (digested sample amount):

4 $${{{\mathrm{Correction}}}}\;{{{\mathrm{factor}}}} = \frac{{{{{\mathrm{Undigested}}}}\;{{{\mathrm{sample}}}}\;{{{\mathrm{amount}}}}}}{{{{{\mathrm{Digested}}}}\;{{{\mathrm{sample}}}}\;{{{\mathrm{amount}}}}}}$$

##### Methylation stoichiometry of single-m6A sites

Most m6A modifications occur in m6A motifs with a core ACA sequence, and these are collectively referred to as m6ACA sites.^70^ Most m6ACA sites consist of a single ACA sequence that can be profiled for m6A methylation at single-nucleotide resolution.

##### Single-m6ACA site

An ACA site with the closest neighbouring ACA at least 40 nt away was defined as a quantifiable single ACA site. An m6ACA site is interrogated by hybridisation with an array probe across the (ACA) sequence in its middle (all of these probe types were marked with “single”). If the site is unmethylated, MazF cleaves the ACA to prevent or greatly reduce probe binding, thus providing a way to quantify methylation levels. If there is another ACA site in close proximity, it can interfere with, or even prevent, the accurate detection and quantification of the interrogated site. However, if the neighbouring ACA is > 40 nt away from the interrogated site, it does not affect the probe signal. Thus, m6A modification in single ACA sites can be quantitatively profiled at a single-nucleotide resolution. We collected all quantifiable single ACA sites based on transcript sequences from the latest Refseq database.

#### Differential “m6A site abundance” profiling

The differential “m6A site abundance” for an m6A site is the sites m6A methylation amount, which is based on the MazF-digested (Cy5-channel) normalised intensity. The quantity was expressed as a value relative to the spike-in reference in a designated sample.

5 $${{{\mathrm{m}}}}6{{{\mathrm{A}}}}\;{{{\mathrm{quantity}}}} = {\rm{Digested}}_{{\rm{Cy}}5\;{\rm{normalized}}\;{\rm{intensity}}}$$

which was calculated by normalising the log_2_ scaled raw intensities of MazF-digested (Cy5-labelled) with the average of the log_2_-scaled spike-in RNA intensities:

6 $$\begin{array}{l}{{{{\log}}}}_2\left( {{\rm{Digested}}_{{\rm{Cy}}5\;{\rm{normalized}}\;{\rm{intensity}}}} \right) = \\ {{{{\log}}}}_2\left( {{\rm{Digested}}_{{\rm{Cy}}5\;{\rm{raw}}}} \right) - {{{\mathrm{Average}}}}\left[ {{{{{\log}}}}_2\left( {{\rm{Digested}}_{{\rm{spike}} - {\rm{in}}\_{\rm{Cy}}5\;{\rm{raw}}}} \right)} \right]\end{array}$$

### MazF-PCR

The m6A methylation levels of the candidate genes were detected by MazF-PCR. The RNA from the 10 BMSC samples was divided into two fractions. One fraction denoted as “MazF-digested” was treated with RNA endoribonuclease MazF to cleave the unmethylated ACA site, while m6A-methylated sites are left unaffected; the other fraction denoted as “MazF-undigested” was untreated with MazF for both m6A-methylated and unmethylated sites. Then, cDNA was synthesised from 500 ng aliquots of MazF-treated RNA and untreated RNA using 5 × First-Strand Buffer (Invitrogen, Carlsbad, CA, USA), 0.1 mol·L^−1^ DTT (Cat No.707265 ML, Invitrogen), RNase Inhibitor (Epicentre, Madison, Wisconsin, USA), and SuperScriptTM III Reverse Transcriptase (Invitrogen). Real-time PCR was performed using the 2× PCR master mix (Arraystar) and a ViiA 7 Real-time PCR System. Primers for specific genes are listed in Table S6. We identified the m6A methylation modification site in the candidate mRNA using MazF ability to distinguish between 5′-ACA-3′ and 5′-(m6A)CA-3′. For real-time RT-PCR, 1 μL of each sample was added, and MazF- served as a control. The MazF correction formula was as follows:

7 $$\% \,{{{\mathrm{MazF}}}} - = \left( {2^{--{\rm{CtMazF}} + }} \right)/\left( {2^{--{\rm{CtMazF}} - }} \right) \times 100\%$$

### RT-PCR and real-time RT-PCR

As previously described, RNA extraction, cDNA synthesis, and real-time RT-PCR were carried out. Table S6 contains primers for the candidate genes.

### PPI network

The differentially m6A-methylated genes were entered into the STRING database, which contains extensive information on PPIs, to obtain the interaction relationships between the differentially m6A-methylated genes.^20^ The data were imported into Cytoscape 3.5.1, and a PPI network constructed and then analysed using a network analyser. A protein–protein interaction network diagram was created using the differentially m6A-methylated genes that had interactions with combined scores greater than 0.7. The hub genes were discovered using the cytoHubba plug-in.

### GO analysis and KEGG pathway enrichment analysis

The Gene Ontology (GO) project (http://www.geneontology.org) provides a controlled vocabulary for describing gene and gene product attributes in any organism. Biological process (BP), cellular component (CC), and molecular function (MF) are the three domains covered by the ontology. The statistical significance of the differences between the DE list and the GO annotation list was assessed using Fisher’s exact test in Bioconductor’s topGO. The significance of the GO term enrichment for DE genes is indicated by the P-value generated by topGO. Genes are mapped to KEGG pathways in pathway analysis. The ingenuity pathway analysis was used to perform KEGG enrichment analysis, which linked differentially expressed m6A methylation genes to biological pathways, and the significance of the pathway correlated to the conditions is represented by the P-value. For the differential m6A methylation modification genes in the pathway, the statistical significance thresholds were established as |FC| ≥ 1.5 and P-value < 0.05. According to the P-values and degree of enrichment, the top 10 hyper- and hypo-GO terms and pathways were chosen.

### Statistical analysis

The mean ± standard deviation (SD) was used to present experimental data. SPSS 23.0 was used to conduct all statistical analyses. To determine statistical significance, the student’s t-test or one-way ANOVA were used, with *P*<0.05 considered significant.

## Supplementary information

Supplementary Table S1

Supplementary Table S2

Supplementary Table S3

Supplementary Table S4

Supplementary Table S5

Supplementary Table S6

## Data Availability

The data supporting the conclusions of this article are provided in the supplementary data.
