# Supplementary material for: Specific RNA m6A modification sites in bone marrow mesenchymal stem cells from the jawbone marrow of type 2 diabetes patients with dental implant failure
Source: Int J Oral Sci. 2023 Jan 12;15:6. doi: 10.1038/s41368-022-00202-3 (PMC9834262; doi:10.1038/s41368-022-00202-3)
Supplement: Supplementary file 1 — Supplementary Table S1 [file 41368_2022_202_MOESM1_ESM.docx]

**Table S1. List of the hypermethylated genes in DM-BMSCs (based on “m6A site methylation stoichiometry”).**

| **Gene symbol** | **Fold change** | **Regulation in T2DM** | **m6A site Locus** | **m6A location** | **m6A transcript location** | **p‐value** |
| --- | --- | --- | --- | --- | --- | --- |
| ZNF12 | 3.04744856 | hyper | chr7:6729964-6729965_- | 3'UTR | 3161 | 0.00258154 |
| MAFK | 2.95064029 | hyper | chr7:1580299-1580300_+ | 3'UTR | 970 | 0.01441724 |
| NCOA7 | 2.2091228 | hyper | chr6:126210869-126210870_+ | CDS | 2038 | 0.04512547 |
| EPB41L3 | 2.20725869 | hyper | chr18:5393329-5393330_- | 3'UTR | 3826 | 0.03207188 |
| TIFA | 2.16363162 | hyper | chr4:113199276-113199277_- | CDS | 577 | 0.03771636 |
| DHRS4-AS1 | 2.1355496 | hyper | chr14:24409346-24409347_- | 1444 | 1444 | 0.00188908 |
| SPAG5 | 2.00208495 | hyper | chr17:26919722-26919723_- | CDS | 630 | 0.0355107 |
| BIRC6 | 1.99118773 | hyper | chr2:32640997-32640998_+ | CDS | 2772 | 0.00682852 |
| ZBTB37 | 1.90622068 | hyper | chr1:173855263-173855264_+ | 3'UTR | 1844 | 0.0182366 |
| PCED1A | 1.86875751 | hyper | chr20:2820949-2820950_- | 5'UTR | 398 | 0.0129361 |
| FAM83H | 1.84778475 | hyper | chr8:144808148-144808149_- | CDS | 3585 | 0.03394408 |
| ZFP64 | 1.80534614 | hyper | chr20:50769204-50769205_- | CDS | 1668 | 0.00070784 |
| PWWP2B | 1.79518272 | hyper | chr10:134219028-134219029_+ | CDS | 1053 | 0.01034893 |
| PPP1R18 | 1.7870125 | hyper | chr6:30653253-30653254_- | CDS | 772 | 0.03480916 |
| SMIM13 | 1.76375268 | hyper | chr6:11135867-11135868_+ | 3'UTR | 1589 | 0.00842416 |
| MFHAS1 | 1.75675289 | hyper | chr8:8748649-8748650_- | CDS | 2481 | 0.02894083 |
| CCDC59 | 1.7527968 | hyper | chr12:82747008-82747009_- | CDS | 690 | 0.02571495 |
| PRSS56 | 1.73047083 | hyper | chr2:233389949-233389950_+ | CDS | 1681 | 0.02076222 |
| KANSL1L | 1.73029635 | hyper | chr2:211018508-211018509_- | CDS | 1087 | 0.030367 |
| AFDN | 1.72987257 | hyper | chr6:168352087-168352088_+ | CDS | 4123 | 0.01355532 |
| RPUSD4 | 1.72120852 | hyper | chr11:126073184-126073185_- | 3'UTR | 1273 | 0.00902203 |
| HOXB9 | 1.71595625 | hyper | chr17:46703161-46703162_- | CDS | 673 | 0.04665331 |
| MAGI1 | 1.68704976 | hyper | chr3:66024214-66024215_- | 5'UTR | 294 | 0.00760923 |
| NAA60 | 1.68601689 | hyper | chr16:3536011-3536012_+ | 3'UTR | 1683 | 0.04435208 |
| ARL4D | 1.68420813 | hyper | chr17:41477470-41477471_+ | CDS | 543 | 0.01460334 |
| MAPT | 1.65932969 | hyper | chr17:44104710-44104711_+ | 3'UTR | 5826 | 0.03405813 |
| PIP5K1C | 1.64851331 | hyper | chr19:3631323-3631324_- | 3'UTR | 3936 | 0.03298627 |
| CELSR1 | 1.63839288 | hyper | chr22:46930035-46930036_- | CDS | 3031 | 0.01559798 |
| ATXN1L | 1.62263505 | hyper | chr16:71884569-71884570_+ | CDS | 1219 | 0.02511974 |
| BCDIN3D-AS1 | 1.60993205 | hyper | chr12:50232663-50232664_+ | 814 | 814 | 0.01556273 |
| MID1IP1 | 1.59293377 | hyper | chrX:38664572-38664573_+ | CDS | 693 | 0.04103089 |
| ABCA3 | 1.59058073 | hyper | chr16:2326642-2326643_- | 3'UTR | 5840 | 0.04267016 |
| PRRC2C | 1.58903138 | hyper | chr1:171561028-171561029_+ | 3'UTR | 8762 | 0.02592807 |
| NUAK1 | 1.58854921 | hyper | chr12:106461669-106461670_- | CDS | 2275 | 0.0020841 |
| SOGA1 | 1.58838683 | hyper | chr20:35414202-35414203_- | 3'UTR | 6010 | 0.01567046 |
| LRRC14 | 1.58187511 | hyper | chr8:145746821-145746822_+ | CDS | 1602 | 0.0416844 |
| ZBTB18 | 1.57923446 | hyper | chr1:244217647-244217648_+ | CDS | 613 | 0.02448651 |
| EEF1AKMT3 | 1.56994137 | hyper | chr12:58174309-58174310_+ | CDS | 686 | 0.01272259 |
| ZNF609 | 1.54860775 | hyper | chr15:64967656-64967657_+ | CDS | 2603 | 0.02527573 |
| KIAA0513 | 1.53297719 | hyper | chr16:85126471-85126472_+ | 3'UTR | 6049 | 0.00572882 |
| KHDC4 | 1.52777336 | hyper | chr1:155883844-155883845_- | 3'UTR | 1951 | 0.03977887 |
| YTHDC1 | 1.52263912 | hyper | chr4:69179853-69179854_- | CDS | 2510 | 0.04219296 |
| KLF12 | 1.51560114 | hyper | chr13:74420373-74420374_- | CDS | 481 | 0.0321142 |
| DIDO1 | 1.51085808 | hyper | chr20:61512232-61512233_- | CDS | 5386 | 0.03074194 |
