# Supplementary material for: Specific RNA m6A modification sites in bone marrow mesenchymal stem cells from the jawbone marrow of type 2 diabetes patients with dental implant failure
Source: Int J Oral Sci. 2023 Jan 12;15:6. doi: 10.1038/s41368-022-00202-3 (PMC9834262; doi:10.1038/s41368-022-00202-3)
Supplement: Supplementary file 2 — Supplementary Table S2 [file 41368_2022_202_MOESM2_ESM.docx]

**Table S2. List of the hypomethylated genes in DM-BMSCs (based on “m6A site methylation stoichiometry”).**

| **Gene symbol** | **Fold change** | **Regulation in T2DM** | **m6A site Locus** | **m6A location** | **m6A transcript location** | **p‐value** |
| --- | --- | --- | --- | --- | --- | --- |
| TM4SF1 | 0.18160196 | hypo | chr3:149087138-149087139_- | 3'UTR | 1224 | 0.02658702 |
| PRPSAP1 | 0.2731169 | hypo | chr17:74307525-74307526_- | 3'UTR | 1700 | 0.03421391 |
| OTUD5 | 0.29253401 | hypo | chrX:48779710-48779711_- | 3'UTR | 2516 | 0.04494957 |
| GTPBP4 | 0.30070226 | hypo | chr10:1063269-1063270_+ | 3'UTR | 2078 | 0.00420607 |
| TAF3 | 0.30169769 | hypo | chr10:8006865-8006866_+ | CDS | 1598 | 0.00233906 |
| CDYL | 0.30272493 | hypo | chr6:4892247-4892248_+ | CDS | 663 | 0.00064297 |
| C1orf50 | 0.30655121 | hypo | chr1:43240958-43240959_+ | CDS | 560 | 0.01679283 |
| PCYT1A | 0.32642993 | hypo | chr3:195961423-195961424_- | 3'UTR | 5360 | 0.03256938 |
| CDV3 | 0.33977841 | hypo | chr3:133307484-133307485_+ | 3'UTR | 1826 | 0.00216971 |
| CHCHD1 | 0.34138653 | hypo | chr10:75543010-75543011_+ | 3'UTR | 450 | 0.04167162 |
| CTBP2 | 0.34262781 | hypo | chr10:126677977-126677978_- | 3'UTR | 3191 | 0.04994272 |
| CA12 | 0.34866656 | hypo | chr15:63617492-63617493_- | 3'UTR | 2181 | 0.01156644 |
| PTTG1IP | 0.3518102 | hypo | chr21:46269800-46269801_- | 3'UTR | 2524 | 0.01823071 |
| CCDC14 | 0.35273937 | hypo | chr3:123633712-123633713_- | CDS | 2563 | 0.01673741 |
| ZNF611 | 0.35481187 | hypo | chr19:53208172-53208173_- | 3'UTR | 2309 | 0.00088275 |
| CORO1C | 0.35727543 | hypo | chr12:109041245-109041246_- | CDS | 1565 | 0.03061886 |
| SPATA13 | 0.35796773 | hypo | chr13:24878789-24878790_+ | 3'UTR | 6040 | 0.03888662 |
| POLL | 0.35996196 | hypo | chr10:103339350-103339351_- | CDS | 2068 | 0.0228243 |
| MON2 | 0.362087 | hypo | chr12:62986604-62986605_+ | 3'UTR | 5620 | 0.01577232 |
| CYBC1 | 0.36380855 | hypo | chr17:80400951-80400952_- | 3'UTR | 1881 | 0.00742702 |
| SMCHD1 | 0.36630939 | hypo | chr18:2802711-2802712_+ | 3'UTR | 6368 | 0.01903077 |
| SRSF5 | 0.36708689 | hypo | chr14:70238573-70238574_+ | 3'UTR | 1398 | 0.00718781 |
| MYNN | 0.36717967 | hypo | chr3:169496792-169496793_+ | CDS | 534 | 0.0024862 |
| PARP9 | 0.3702948 | hypo | chr3:122247317-122247318_- | CDS | 2679 | 0.00351319 |
| TMEM248 | 0.3726874 | hypo | chr7:66420633-66420634_+ | 3'UTR | 1333 | 0.0026531 |
| NAB1 | 0.37345513 | hypo | chr2:191555162-191555163_+ | 3'UTR | 2139 | 0.00758735 |
| NIPBL | 0.37552376 | hypo | chr5:36985840-36985841_+ | CDS | 3057 | 0.02635656 |
| PAPOLA | 0.37657812 | hypo | chr14:97031389-97031390_+ | 3'UTR | 2457 | 0.01969666 |
| MINDY3 | 0.37753159 | hypo | chr10:15820956-15820957_- | 3'UTR | 1605 | 0.00702921 |
| ENC1 | 0.39073442 | hypo | chr5:73930697-73930698_- | CDS | 2743 | 0.01682202 |
| CRKL | 0.39144692 | hypo | chr22:21307461-21307462_+ | 3'UTR | 4749 | 0.03820363 |
| SIRT7 | 0.39233578 | hypo | chr17:79870272-79870273_- | 3'UTR | 1272 | 0.00629188 |
| BAG5 | 0.39569905 | hypo | chr14:104026688-104026689_- | CDS | 1246 | 0.00569083 |
| SLC16A4 | 0.39771055 | hypo | chr1:110921788-110921789_- | CDS | 909 | 0.00744992 |
| ZNF106 | 0.39874495 | hypo | chr15:42740468-42740469_- | CDS | 3228 | 0.00229061 |
| SLC16A3 | 0.40326692 | hypo | chr17:80197161-80197162_+ | 3'UTR | 1873 | 0.02296871 |
| FAM104A | 0.40477658 | hypo | chr17:71203792-71203793_- | 3'UTR | 2586 | 0.04783981 |
| ZFP62 | 0.40554175 | hypo | chr5:180277703-180277704_- | CDS | 857 | 0.02271765 |
| LACTB | 0.40776187 | hypo | chr15:63433674-63433675_+ | CDS | 1386 | 0.04098108 |
| RAB9A | 0.40924098 | hypo | chrX:13727023-13727024_+ | CDS | 352 | 0.01283143 |
| DDX19A | 0.41135847 | hypo | chr16:70406532-70406533_+ | 3'UTR | 2369 | 0.04558889 |
| ABHD13 | 0.41219101 | hypo | chr13:108882501-108882502_+ | CDS | 1200 | 0.01066045 |
| ERC1 | 0.41253042 | hypo | chr12:1600189-1600190_+ | 3'UTR | 4415 | 0.00268688 |
| STX18 | 0.41834869 | hypo | chr4:4421505-4421506_- | 3'UTR | 1364 | 0.00389677 |
| PDGFC | 0.41969248 | hypo | chr4:157683026-157683027_- | 3'UTR | 2743 | 0.02017345 |
| UACA | 0.4198829 | hypo | chr15:70961480-70961481_- | CDS | 1727 | 0.01660524 |
| PHKA1 | 0.42147514 | hypo | chrX:71800678-71800679_- | 3'UTR | 4145 | 0.00232665 |
| C9orf3 | 0.42246363 | hypo | chr9:97849259-97849260_+ | 3'UTR | 2895 | 0.01106853 |
| HSP90AA1 | 0.4224995 | hypo | chr14:102547623-102547624_- | 3'UTR | 3334 | 0.01283575 |
| SETD2 | 0.42416812 | hypo | chr3:47162150-47162151_- | CDS | 4027 | 0.01637563 |
| SREK1 | 0.42503565 | hypo | chr5:65474598-65474599_+ | CDS | 1936 | 0.0116144 |
| MPPE1 | 0.42547054 | hypo | chr18:11884451-11884452_- | CDS | 1978 | 0.00616998 |
| NEO1 | 0.42611067 | hypo | chr15:73595450-73595451_+ | 3'UTR | 5012 | 0.01549368 |
| C2CD2 | 0.42686481 | hypo | chr21:43305367-43305368_- | 3'UTR | 6197 | 0.03559964 |
| MYL6 | 0.42932168 | hypo | chr12:56553872-56553873_+ | CDS | 430 | 0.0224011 |
| ADNP | 0.43010069 | hypo | chr20:49507916-49507917_- | 3'UTR | 3726 | 0.01530922 |
| RRAGC | 0.43142998 | hypo | chr1:39304703-39304704_- | 3'UTR | 1897 | 0.02086609 |
| PDHB | 0.43305688 | hypo | chr3:58413774-58413775_- | CDS | 1088 | 0.02580479 |
| C6orf62 | 0.43524505 | hypo | chr6:24706187-24706188_- | 3'UTR | 1373 | 0.00083619 |
| DFFA | 0.43596093 | hypo | chr1:10522855-10522856_- | 3'UTR | 1139 | 0.02188533 |
| KPNA2 | 0.43604104 | hypo | chr17:66039242-66039243_+ | CDS | 955 | 0.01674721 |
| ST3GAL5 | 0.43708539 | hypo | chr2:86066650-86066651_- | 3'UTR | 2001 | 0.01915748 |
| HSP90AA1 | 0.43724608 | hypo | chr14:102552596-102552597_- | CDS | 829 | 0.01076392 |
| FBXO42 | 0.43786974 | hypo | chr1:16577199-16577200_- | CDS | 2372 | 0.02399498 |
| RCN2 | 0.43933611 | hypo | chr15:77241447-77241448_+ | CDS | 1059 | 0.04305002 |
| CDK9 | 0.44084505 | hypo | chr9:130552912-130552913_+ | 3'UTR | 2332 | 0.00037279 |
| SSR2 | 0.44104638 | hypo | chr1:155979115-155979116_- | 3'UTR | 846 | 0.01238151 |
| PPP1CB | 0.44149276 | hypo | chr2:29023636-29023637_+ | 3'UTR | 2611 | 0.01732617 |
| POP4 | 0.44292053 | hypo | chr19:30106474-30106475_+ | 3'UTR | 906 | 0.02490614 |
| HSP90AA1 | 0.44299645 | hypo | chr14:102552453-102552454_- | CDS | 880 | 0.00963362 |
| LAP3 | 0.44537941 | hypo | chr4:17609252-17609253_+ | 3'UTR | 1762 | 0.00319883 |
| CCDC47 | 0.44695472 | hypo | chr17:61829773-61829774_- | CDS | 1444 | 0.00714204 |
| HMGN3 | 0.44699259 | hypo | chr6:79911270-79911271_- | 3'UTR | 704 | 0.00375125 |
| YTHDF3 | 0.44843607 | hypo | chr8:64099644-64099645_+ | CDS | 1238 | 0.01275251 |
| UACA | 0.44847662 | hypo | chr15:70961651-70961652_- | CDS | 1556 | 0.02511271 |
| CUL2 | 0.44935918 | hypo | chr10:35299040-35299041_- | 3'UTR | 2709 | 0.00763396 |
| PRPF38A | 0.44943648 | hypo | chr1:52883512-52883513_+ | 3'UTR | 2237 | 0.00353733 |
| NFE2L2 | 0.4505963 | hypo | chr2:178096059-178096060_- | CDS | 1825 | 0.01111782 |
| APP | 0.45398861 | hypo | chr21:27264151-27264152_- | CDS | 2242 | 0.02069564 |
| CNPY2 | 0.45402112 | hypo | chr12:56709712-56709713_- | 5'UTR | 419 | 0.03641593 |
| FAM104A | 0.45619757 | hypo | chr17:71205634-71205635_- | 3'UTR | 744 | 0.00254902 |
| KBTBD2 | 0.45628389 | hypo | chr7:32908898-32908899_- | 3'UTR | 2588 | 0.00731946 |
| USP47 | 0.45651822 | hypo | chr11:11977806-11977807_+ | 3'UTR | 4711 | 0.01345406 |
| MRFAP1 | 0.45763387 | hypo | chr4:6644121-6644122_+ | 3'UTR | 1833 | 0.0272829 |
| TBC1D9 | 0.45765855 | hypo | chr4:141543415-141543416_- | CDS | 4005 | 0.02088737 |
| TCFL5 | 0.45909703 | hypo | chr20:61473295-61473296_- | 3'UTR | 1626 | 0.01377314 |
| TMEM51 | 0.45932099 | hypo | chr1:15546710-15546711_+ | 3'UTR | 1648 | 0.03332103 |
| NCBP3 | 0.45938174 | hypo | chr17:3715661-3715662_- | 3'UTR | 2561 | 0.03841993 |
| NADK2 | 0.45957576 | hypo | chr5:36195078-36195079_- | 3'UTR | 1622 | 0.01785252 |
| CAPN7 | 0.45989432 | hypo | chr3:15292805-15292806_+ | 3'UTR | 2750 | 0.00815532 |
| TRMT10C | 0.46017201 | hypo | chr3:101284764-101284765_+ | CDS | 1319 | 0.00342679 |
| NIP7 | 0.46130319 | hypo | chr16:69375514-69375515_+ | CDS | 820 | 0.01752807 |
| UTP3 | 0.46293135 | hypo | chr4:71555226-71555227_+ | CDS | 1031 | 0.03980143 |
| RND3 | 0.463975 | hypo | chr2:151326672-151326673_- | CDS | 840 | 0.01564608 |
| OSTC | 0.46405262 | hypo | chr4:109588836-109588837_+ | 3'UTR | 1010 | 0.03752135 |
| NRIP1 | 0.46418199 | hypo | chr21:16339107-16339108_- | CDS | 2003 | 0.00077433 |
| H2AFY2 | 0.46419655 | hypo | chr10:71871688-71871689_+ | 3'UTR | 1827 | 0.00289181 |
| TBC1D8 | 0.46591766 | hypo | chr2:101624420-101624421_- | CDS | 3478 | 0.0178824 |
| C1orf50 | 0.46668097 | hypo | chr1:43240994-43240995_+ | CDS | 596 | 0.00994474 |
| GNE | 0.46714179 | hypo | chr9:36217279-36217280_- | 3'UTR | 2455 | 0.01212164 |
| NECAP2 | 0.46753575 | hypo | chr1:16785596-16785597_+ | 3'UTR | 1017 | 0.00712764 |
| ERO1A | 0.46761921 | hypo | chr14:53110255-53110256_- | 3'UTR | 1888 | 0.04515373 |
| ATG5 | 0.46819644 | hypo | chr6:106634288-106634289_- | 3'UTR | 1306 | 0.04415538 |
| IP6K2 | 0.46849735 | hypo | chr3:48725802-48725803_- | CDS | 1432 | 0.04390797 |
| CAPZA2 | 0.46905089 | hypo | chr7:116557926-116557927_+ | 3'UTR | 891 | 0.01330545 |
| RAB5A | 0.46921429 | hypo | chr3:20025541-20025542_+ | 3'UTR | 1409 | 0.00583589 |
| LSG1 | 0.46991081 | hypo | chr3:194362326-194362327_- | 3'UTR | 2761 | 0.03065351 |
| TMEM30A | 0.47027092 | hypo | chr6:75965233-75965234_- | 3'UTR | 1947 | 0.00963115 |
| ZDHHC16 | 0.47033896 | hypo | chr10:99216820-99216821_+ | 3'UTR | 1534 | 0.00400094 |
| CEP120 | 0.47081201 | hypo | chr5:122682209-122682210_- | 3'UTR | 3079 | 0.02643078 |
| N4BP2L2 | 0.47102254 | hypo | chr13:33016803-33016804_- | CDS | 1977 | 0.01659853 |
| PDIA6 | 0.47189202 | hypo | chr2:10924206-10924207_- | 3'UTR | 1642 | 0.01410467 |
| PHF5A | 0.47192392 | hypo | chr22:41855952-41855953_- | 3'UTR | 832 | 0.00995653 |
| FRYL | 0.47305124 | hypo | chr4:48501436-48501437_- | 3'UTR | 9648 | 0.00972499 |
| ZNF184 | 0.47305744 | hypo | chr6:27419276-27419277_- | CDS | 2345 | 0.01869807 |
| SCAF11 | 0.47314175 | hypo | chr12:46318836-46318837_- | CDS | 3866 | 0.01485306 |
| PLOD2 | 0.47458877 | hypo | chr3:145788449-145788450_- | 3'UTR | 2836 | 0.00433865 |
| ELAC2 | 0.47520268 | hypo | chr17:12895848-12895849_- | 3'UTR | 2763 | 0.02086201 |
| TAF7 | 0.47643678 | hypo | chr5:140698664-140698665_- | CDS | 1686 | 0.04564683 |
| RCAN1 | 0.47824906 | hypo | chr21:35890113-35890114_- | 3'UTR | 1128 | 0.00084156 |
| ITPRIPL2 | 0.47830653 | hypo | chr16:19128945-19128946_+ | 3'UTR | 3692 | 0.00700385 |
| CHML | 0.47894303 | hypo | chr1:241797666-241797667_- | CDS | 1565 | 0.02847601 |
| AFF4 | 0.47975847 | hypo | chr5:132232626-132232627_- | CDS | 2074 | 0.00678553 |
| PPP2R5E | 0.48061531 | hypo | chr14:63842534-63842535_- | 3'UTR | 2201 | 0.01863995 |
| SNU13 | 0.48094142 | hypo | chr22:42070218-42070219_- | 3'UTR | 1217 | 0.0120625 |
| TAF12 | 0.48120458 | hypo | chr1:28930026-28930027_- | 3'UTR | 694 | 0.00310157 |
| ARHGAP17 | 0.48162254 | hypo | chr16:24931198-24931199_- | 3'UTR | 3014 | 0.02606955 |
| DNAAF5 | 0.48164582 | hypo | chr7:825777-825778_+ | 3'UTR | 3075 | 0.01965355 |
| LPCAT1 | 0.48252741 | hypo | chr5:1461709-1461710_- | 3'UTR | 3776 | 0.01600211 |
| YWHAH | 0.48288694 | hypo | chr22:32352631-32352632_+ | CDS | 834 | 0.00825199 |
| CCT5 | 0.48318773 | hypo | chr5:10258557-10258558_+ | CDS | 1203 | 0.02108016 |
| CNBP | 0.48378792 | hypo | chr3:128889959-128889960_- | CDS | 589 | 0.01247574 |
| PDZD8 | 0.48387023 | hypo | chr10:119044163-119044164_- | CDS | 2319 | 0.01223969 |
| TSPYL1 | 0.48400231 | hypo | chr6:116600054-116600055_- | CDS | 1225 | 0.00122696 |
| ZBTB5 | 0.48525593 | hypo | chr9:37438676-37438677_- | 3'UTR | 4071 | 0.023848 |
| ARL6IP5 | 0.48593867 | hypo | chr3:69153787-69153788_+ | CDS | 678 | 0.00970562 |
| RND3 | 0.48633663 | hypo | chr2:151326476-151326477_- | 3'UTR | 1036 | 0.03726064 |
| CCN2 | 0.48642385 | hypo | chr6:132270386-132270387_- | 3'UTR | 1266 | 0.01442248 |
| KAT5 | 0.48788143 | hypo | chr11:65486656-65486657_+ | 3'UTR | 1911 | 0.00096032 |
| PSME4 | 0.4879074 | hypo | chr2:54092390-54092391_- | 3'UTR | 5911 | 0.01123485 |
| GDF11 | 0.48827471 | hypo | chr12:56143316-56143317_+ | CDS | 911 | 0.04115872 |
| NIP7 | 0.48854824 | hypo | chr16:69375573-69375574_+ | 3'UTR | 879 | 0.00676522 |
| BPTF | 0.49211196 | hypo | chr17:65850337-65850338_+ | CDS | 956 | 0.04164115 |
| EAPP | 0.49237468 | hypo | chr14:34985463-34985464_- | 3'UTR | 1018 | 0.0166732 |
| FDFT1 | 0.49265816 | hypo | chr8:11695938-11695939_+ | CDS | 1296 | 0.00861863 |
| C1QBP | 0.49328566 | hypo | chr17:5336283-5336284_- | 3'UTR | 977 | 0.0071271 |
| RBMXL1 | 0.49432504 | hypo | chr1:89449128-89449129_- | CDS | 996 | 0.0355984 |
| TBC1D8 | 0.49494366 | hypo | chr2:101623981-101623982_- | 3'UTR | 3917 | 0.00520336 |
| TCF3 | 0.49529477 | hypo | chr19:1611280-1611281_- | 3'UTR | 2085 | 0.0107955 |
| RNASEH1 | 0.49583118 | hypo | chr2:3595530-3595531_- | CDS | 874 | 0.04470595 |
| RND3 | 0.4958917 | hypo | chr2:151326634-151326635_- | CDS | 878 | 0.00472175 |
| FBXO11 | 0.49609568 | hypo | chr2:48035024-48035025_- | 3'UTR | 3088 | 0.00135997 |
| MYL12B | 0.49673197 | hypo | chr18:3277991-3277992_+ | 3'UTR | 715 | 0.01161175 |
| HECTD1 | 0.49707897 | hypo | chr14:31576304-31576305_- | CDS | 7571 | 0.0316184 |
| SHISA5 | 0.4973113 | hypo | chr3:48509466-48509467_- | 3'UTR | 1771 | 0.00421979 |
| SP3 | 0.49750298 | hypo | chr2:174774817-174774818_- | CDS | 2727 | 0.01281129 |
| U2SURP | 0.49838724 | hypo | chr3:142775172-142775173_+ | CDS | 3069 | 0.00726223 |
| DNAJB9 | 0.49852955 | hypo | chr7:108213350-108213351_+ | CDS | 594 | 0.03241039 |
| APC | 0.49874638 | hypo | chr5:112179399-112179400_+ | CDS | 8167 | 0.00866462 |
| SHQ1 | 0.49984317 | hypo | chr3:72799633-72799634_- | CDS | 1641 | 0.0491708 |
| GPATCH8 | 0.50154866 | hypo | chr17:42477702-42477703_- | CDS | 1777 | 0.02728662 |
| METAP2 | 0.50163244 | hypo | chr12:95907824-95907825_+ | 3'UTR | 1715 | 0.01328509 |
| SLC30A1 | 0.50170355 | hypo | chr1:211748832-211748833_- | CDS | 1565 | 0.02516873 |
| HAUS7 | 0.50266389 | hypo | chrX:152713171-152713172_- | 3'UTR | 1314 | 0.0164156 |
| NMRAL1 | 0.50281892 | hypo | chr16:4513748-4513749_- | CDS | 1007 | 0.00529042 |
| INHBA | 0.50425023 | hypo | chr7:41729533-41729534_- | CDS | 1241 | 0.03430091 |
| G2E3 | 0.50483182 | hypo | chr14:31085609-31085610_+ | CDS | 2109 | 0.00262891 |
| KDELR2 | 0.5048528 | hypo | chr7:6501872-6501873_- | 3'UTR | 1632 | 0.02391672 |
| RPL41 | 0.50495815 | hypo | chr12:56511522-56511523_+ | 3'UTR | 490 | 0.03500692 |
| DNAJA1 | 0.50501263 | hypo | chr9:33038915-33038916_+ | 3'UTR | 1399 | 0.00344057 |
| GFOD2 | 0.50512087 | hypo | chr16:67709067-67709068_- | CDS | 1388 | 0.04200411 |
| RILPL1 | 0.5052236 | hypo | chr12:123956932-123956933_- | 3'UTR | 1599 | 0.01276995 |
| SEMA3A | 0.50583958 | hypo | chr7:83590660-83590661_- | 3'UTR | 2656 | 0.02765966 |
| YTHDF2 | 0.50599875 | hypo | chr1:29070176-29070177_+ | CDS | 1657 | 0.01375708 |
| NETO2 | 0.50614347 | hypo | chr16:47117495-47117496_- | CDS | 1626 | 0.00132241 |
| MLEC | 0.50616232 | hypo | chr12:121134209-121134210_+ | CDS | 913 | 0.02725761 |
| ALDH1B1 | 0.50698376 | hypo | chr9:38398128-38398129_+ | 3'UTR | 2536 | 0.03655604 |
| CAMLG | 0.50749294 | hypo | chr5:134076914-134076915_+ | CDS | 475 | 0.01495831 |
| CCDC88A | 0.50778696 | hypo | chr2:55561523-55561524_- | CDS | 3274 | 0.01535595 |
| GPR161 | 0.50818363 | hypo | chr1:168054618-168054619_- | 3'UTR | 1967 | 0.00490274 |
| CPSF2 | 0.50842615 | hypo | chr14:92628069-92628070_+ | CDS | 2598 | 0.02998529 |
| IFNGR1 | 0.5084629 | hypo | chr6:137519124-137519125_- | 3'UTR | 1615 | 0.01119806 |
| RGS2 | 0.50851727 | hypo | chr1:192780914-192780915_+ | 3'UTR | 857 | 0.03027827 |
| URI1 | 0.50870403 | hypo | chr19:30500008-30500009_+ | CDS | 1092 | 0.00326924 |
| SLC25A37 | 0.50886193 | hypo | chr8:23423789-23423790_+ | CDS | 587 | 0.0019015 |
| CCDC107 | 0.50922403 | hypo | chr9:35660828-35660829_+ | CDS | 586 | 0.00331757 |
| CDR2 | 0.50978904 | hypo | chr16:22357719-22357720_- | 3'UTR | 2240 | 0.00032268 |
| NFYA | 0.51061961 | hypo | chr6:41065491-41065492_+ | 3'UTR | 1564 | 0.04520327 |
| USP34 | 0.5108555 | hypo | chr2:61415600-61415601_- | CDS | 10298 | 0.01697235 |
| MPHOSPH8 | 0.51130861 | hypo | chr13:20221312-20221313_+ | CDS | 1200 | 0.01881162 |
| CTNNB1 | 0.511313 | hypo | chr3:41266506-41266507_+ | CDS | 571 | 0.02046737 |
| RBBP6 | 0.51138746 | hypo | chr16:24583172-24583173_+ | CDS | 5825 | 0.01175461 |
| DDHD1 | 0.51141591 | hypo | chr14:53513471-53513472_- | 3'UTR | 2946 | 0.0059473 |
| MRPL19 | 0.51221291 | hypo | chr2:75882497-75882498_+ | 3'UTR | 990 | 0.00251561 |
| DLGAP4 | 0.51222465 | hypo | chr20:35155498-35155499_+ | 3'UTR | 3527 | 0.0374709 |
| ARFGAP1 | 0.51239662 | hypo | chr20:61919933-61919934_+ | 3'UTR | 2093 | 0.02883586 |
| RBM17 | 0.51257302 | hypo | chr10:6157509-6157510_+ | CDS | 1422 | 0.04982097 |
| HSPA2 | 0.51290682 | hypo | chr14:65008930-65008931_+ | CDS | 1745 | 0.01545147 |
| LRRC59 | 0.51328398 | hypo | chr17:48460279-48460280_- | 3'UTR | 1228 | 0.04531245 |
| CLIP1 | 0.51383019 | hypo | chr12:122825799-122825800_- | CDS | 2105 | 0.00606008 |
| ZDHHC3 | 0.51391396 | hypo | chr3:44968085-44968086_- | 3'UTR | 1352 | 0.04521057 |
| HSP90AA1 | 0.51412009 | hypo | chr14:102551670-102551671_- | CDS | 1337 | 0.00924987 |
| TOMM7 | 0.51419494 | hypo | chr7:22852706-22852707_- | 3'UTR | 322 | 0.01721276 |
| ADD3 | 0.51429433 | hypo | chr10:111885622-111885623_+ | CDS | 1833 | 0.01592599 |
| HNRNPU | 0.51481779 | hypo | chr1:245021531-245021532_- | CDS | 1492 | 0.00237868 |
| NUP50 | 0.51555668 | hypo | chr22:45581481-45581482_+ | 3'UTR | 2734 | 0.01408348 |
| HEXIM1 | 0.51609443 | hypo | chr17:43227099-43227100_+ | CDS | 2416 | 0.01460427 |
| FJX1 | 0.51609761 | hypo | chr11:35641919-35641920_+ | 3'UTR | 2185 | 0.02634394 |
| COLEC12 | 0.51647846 | hypo | chr18:319992-319993_- | 3'UTR | 2495 | 0.01347029 |
| SPG7 | 0.51723917 | hypo | chr16:89623422-89623423_+ | CDS | 2339 | 0.00302687 |
| RNF169 | 0.51726251 | hypo | chr11:74553367-74553368_+ | 3'UTR | 7732 | 0.01962011 |
| TBC1D15 | 0.51765286 | hypo | chr12:72317011-72317012_+ | 3'UTR | 2178 | 0.00301587 |
| HSP90AA1 | 0.51815376 | hypo | chr14:102548756-102548757_- | CDS | 2490 | 0.00434527 |
| DYNLL1 | 0.51841229 | hypo | chr12:120936053-120936054_+ | 3'UTR | 491 | 0.02557126 |
| FRYL | 0.5185942 | hypo | chr4:48501291-48501292_- | 3'UTR | 9793 | 0.01513653 |
| TIMM17A | 0.51928838 | hypo | chr1:201938835-201938836_+ | 3'UTR | 705 | 0.02446299 |
| NUCKS1 | 0.5193511 | hypo | chr1:205687035-205687036_- | 3'UTR | 1374 | 0.01761686 |
| COA6 | 0.51949918 | hypo | chr1:234519597-234519598_+ | 3'UTR | 447 | 0.00658488 |
| PRPF18 | 0.51979793 | hypo | chr10:13672363-13672364_+ | 3'UTR | 1174 | 0.00199888 |
| IL13RA1 | 0.51986893 | hypo | chrX:117925929-117925930_+ | 3'UTR | 1439 | 0.04875076 |
| GALNT10 | 0.52015362 | hypo | chr5:153800159-153800160_+ | 3'UTR | 5571 | 0.02249151 |
| NMT1 | 0.52021532 | hypo | chr17:43186168-43186169_+ | 3'UTR | 4683 | 0.04449617 |
| CTPS1 | 0.52066488 | hypo | chr1:41477744-41477745_+ | 3'UTR | 2347 | 0.04698836 |
| RHOA | 0.52079911 | hypo | chr3:49400055-49400056_- | CDS | 439 | 0.03470555 |
| HARS | 0.52222655 | hypo | chr5:140053786-140053787_- | 3'UTR | 1650 | 0.02499125 |
| RPL37A | 0.52311106 | hypo | chr2:217364725-217364726_+ | CDS | 264 | 0.01942611 |
| NIF3L1 | 0.52335704 | hypo | chr2:201768375-201768376_+ | CDS | 1199 | 0.0224675 |
| MTMR3 | 0.52367273 | hypo | chr22:30416306-30416307_+ | CDS | 2986 | 0.03560473 |
| GAS2L3 | 0.52513063 | hypo | chr12:101017826-101017827_+ | CDS | 1519 | 0.01046064 |
| PVR | 0.5252364 | hypo | chr19:45166499-45166500_+ | 3'UTR | 2974 | 0.01001466 |
| MANF | 0.5252554 | hypo | chr3:51426617-51426618_+ | 3'UTR | 698 | 0.01998835 |
| TRAM2 | 0.52529564 | hypo | chr6:52362541-52362542_- | 3'UTR | 6714 | 0.03514114 |
| DSP | 0.52581338 | hypo | chr6:7584136-7584137_+ | CDS | 6982 | 0.02850858 |
| DNAJB1 | 0.52597663 | hypo | chr19:14626789-14626790_- | CDS | 1194 | 0.01018583 |
| SLC35G2 | 0.52663243 | hypo | chr3:136573591-136573592_+ | CDS | 503 | 0.01368531 |
| NME4 | 0.52693356 | hypo | chr16:450564-450565_+ | 3'UTR | 817 | 0.02893893 |
| FBXL20 | 0.52703458 | hypo | chr17:37417712-37417713_- | CDS | 1564 | 0.01835485 |
| PRSS23 | 0.52714558 | hypo | chr11:86519532-86519533_+ | CDS | 970 | 0.0103208 |
| DDX6 | 0.52758524 | hypo | chr11:118656964-118656965_- | 5'UTR | 356 | 0.03603269 |
| S100PBP | 0.5276347 | hypo | chr1:33292100-33292101_+ | CDS | 691 | 0.00359334 |
| E4F1 | 0.52807078 | hypo | chr16:2285490-2285491_+ | CDS | 2398 | 0.03700252 |
| MSANTD3 | 0.52808875 | hypo | chr9:103213302-103213303_+ | 3'UTR | 1165 | 0.01241232 |
| CDC16 | 0.52832095 | hypo | chr13:115037770-115037771_+ | CDS | 1955 | 0.00284253 |
| SEC61A1 | 0.52841624 | hypo | chr3:127789110-127789111_+ | 3'UTR | 2220 | 0.04788322 |
| TUT7 | 0.52851706 | hypo | chr9:88967683-88967684_- | CDS | 678 | 0.02463906 |
| PSMA2 | 0.52905259 | hypo | chr7:42957042-42957043_- | 3'UTR | 882 | 0.00690733 |
| PIGO | 0.52956696 | hypo | chr9:35089000-35089001_- | 3'UTR | 3753 | 0.00585462 |
| GLT8D1 | 0.52968553 | hypo | chr3:52728691-52728692_- | 3'UTR | 1515 | 0.00961284 |
| METAP2 | 0.53178382 | hypo | chr12:95905695-95905696_+ | CDS | 1123 | 0.02344724 |
| UTP15 | 0.53197029 | hypo | chr5:72875934-72875935_+ | 3'UTR | 1827 | 0.02857525 |
| TFDP1 | 0.53200638 | hypo | chr13:114295053-114295054_+ | 3'UTR | 1916 | 0.01201074 |
| LDHB | 0.53265832 | hypo | chr12:21788503-21788504_- | CDS | 1087 | 0.03695999 |
| EEF2 | 0.53360482 | hypo | chr19:3976229-3976230_- | 3'UTR | 2981 | 0.00821521 |
| KLHL42 | 0.53380155 | hypo | chr12:27950821-27950822_+ | CDS | 1317 | 0.00682258 |
| ZADH2 | 0.53380261 | hypo | chr18:72912845-72912846_- | 3'UTR | 1948 | 0.00872161 |
| TCEAL8 | 0.53438725 | hypo | chrX:102508626-102508627_- | CDS | 434 | 0.00820857 |
| CRKL | 0.53464281 | hypo | chr22:21307375-21307376_+ | 3'UTR | 4663 | 0.00621282 |
| SSH2 | 0.53475848 | hypo | chr17:27959198-27959199_- | CDS | 2971 | 0.03541011 |
| RPS15 | 0.5349956 | hypo | chr19:1438881-1438882_+ | CDS | 141 | 0.01142986 |
| NUP133 | 0.53535222 | hypo | chr1:229600423-229600424_- | CDS | 2589 | 0.00509223 |
| LRP10 | 0.5355528 | hypo | chr14:23347056-23347057_+ | 3'UTR | 3049 | 0.00650555 |
| MAP1B | 0.53560162 | hypo | chr5:71491105-71491106_+ | CDS | 2187 | 0.0132311 |
| EIF4E2 | 0.53564202 | hypo | chr2:233445809-233445810_+ | 3'UTR | 999 | 0.01010817 |
| TCP1 | 0.53586114 | hypo | chr6:160200053-160200054_- | 3'UTR | 1928 | 0.00314089 |
| SMARCA4 | 0.53611448 | hypo | chr19:11172608-11172609_+ | 3'UTR | 5230 | 0.03035767 |
| CD2BP2 | 0.53613682 | hypo | chr16:30364988-30364989_- | CDS | 683 | 0.00963436 |
| ACTB | 0.53625618 | hypo | chr7:5567318-5567319_- | 3'UTR | 1379 | 0.00874561 |
| SETD1A | 0.53648216 | hypo | chr16:30991422-30991423_+ | CDS | 5001 | 0.02736154 |
| SPPL3 | 0.5375615 | hypo | chr12:121202468-121202469_- | 3'UTR | 1972 | 0.01752767 |
| COX7A2L | 0.53779129 | hypo | chr2:42578365-42578366_- | CDS | 419 | 0.01743128 |
| RGS10 | 0.53801524 | hypo | chr10:121259631-121259632_- | CDS | 619 | 0.03000872 |
| VOPP1 | 0.53812703 | hypo | chr7:55539115-55539116_- | 3'UTR | 2146 | 0.01096425 |
| MRPS14 | 0.53813341 | hypo | chr1:174983647-174983648_- | 3'UTR | 590 | 0.00133004 |
| CHCHD1 | 0.53889143 | hypo | chr10:75542951-75542952_+ | 3'UTR | 391 | 0.02357615 |
| MATR3 | 0.53918121 | hypo | chr5:138643495-138643496_+ | CDS | 730 | 0.00740868 |
| MRPL46 | 0.53944524 | hypo | chr15:89003016-89003017_- | CDS | 677 | 0.01650776 |
| EGLN1 | 0.53967002 | hypo | chr1:231556885-231556886_- | CDS | 3904 | 0.02333651 |
| SPPL3 | 0.53967269 | hypo | chr12:121202439-121202440_- | 3'UTR | 2001 | 0.01449853 |
| MAD2L1BP | 0.53969502 | hypo | chr6:43608246-43608247_+ | CDS | 1105 | 0.01525548 |
| NONO | 0.54085813 | hypo | chrX:70519906-70519907_+ | CDS | 1935 | 0.0409648 |
| RAB9A | 0.54086452 | hypo | chrX:13727049-13727050_+ | CDS | 378 | 0.0238535 |
| BOD1L1 | 0.54095709 | hypo | chr4:13600613-13600614_- | CDS | 8026 | 0.03510264 |
| B3GALNT2 | 0.54115585 | hypo | chr1:235613520-235613521_- | CDS | 1731 | 0.03657917 |
| ACO2 | 0.54162581 | hypo | chr22:41924961-41924962_+ | 3'UTR | 2709 | 0.04041798 |
| GALNT7 | 0.54229913 | hypo | chr4:174169492-174169493_+ | CDS | 571 | 0.02528123 |
| FLVCR2 | 0.54248261 | hypo | chr14:76113446-76113447_+ | 3'UTR | 2588 | 0.01540963 |
| TUBA1B | 0.5427952 | hypo | chr12:49521676-49521677_- | 3'UTR | 1640 | 0.02600781 |
| BMPR2 | 0.5428288 | hypo | chr2:203420951-203420952_+ | CDS | 3711 | 0.03160869 |
| COA3 | 0.5428627 | hypo | chr17:40949901-40949902_- | 3'UTR | 520 | 0.02862294 |
| REST | 0.54287153 | hypo | chr4:57797792-57797793_+ | CDS | 2904 | 0.03015351 |
| HRH1 | 0.5430269 | hypo | chr3:11301719-11301720_+ | CDS | 1058 | 0.01480103 |
| DERL2 | 0.54322696 | hypo | chr17:5377765-5377766_- | 3'UTR | 1026 | 0.00526378 |
| CTTNBP2NL | 0.54372563 | hypo | chr1:112999053-112999054_+ | CDS | 1167 | 0.00902506 |
| LAMC1 | 0.54374817 | hypo | chr1:183111722-183111723_+ | CDS | 4884 | 0.01448815 |
| CHST3 | 0.54383958 | hypo | chr10:73768693-73768694_+ | 3'UTR | 2311 | 0.01261401 |
| USP15 | 0.54403912 | hypo | chr12:62798181-62798182_+ | 3'UTR | 3047 | 0.00166969 |
| CISD1 | 0.54464799 | hypo | chr10:60047414-60047415_+ | 3'UTR | 443 | 0.01943596 |
| IKBIP | 0.5449409 | hypo | chr12:99020109-99020110_- | CDS | 1081 | 0.0494234 |
| KLHL42 | 0.54506685 | hypo | chr12:27950881-27950882_+ | CDS | 1377 | 0.00124764 |
| PSMD7 | 0.54517031 | hypo | chr16:74334092-74334093_+ | CDS | 294 | 0.01128139 |
| HAUS6 | 0.54523262 | hypo | chr9:19058304-19058305_- | CDS | 2750 | 0.01850746 |
| CLU | 0.54600376 | hypo | chr8:27466538-27466539_- | CDS | 347 | 0.01266808 |
| USP14 | 0.5464572 | hypo | chr18:211506-211507_+ | 3'UTR | 1849 | 0.02977229 |
| KLF6 | 0.54685996 | hypo | chr10:3819652-3819653_- | 3'UTR | 3124 | 0.03341001 |
| PIGM | 0.54696444 | hypo | chr1:160000042-160000043_- | 3'UTR | 1740 | 0.00934574 |
| NFYA | 0.54768203 | hypo | chr6:41066691-41066692_+ | 3'UTR | 2764 | 0.00360323 |
| LTN1 | 0.54795809 | hypo | chr21:30339397-30339398_- | CDS | 1486 | 0.00100599 |
| PRDX1 | 0.54801039 | hypo | chr1:45980625-45980626_- | CDS | 425 | 0.04023683 |
| HPS6 | 0.54813185 | hypo | chr10:103827623-103827624_+ | 3'UTR | 2500 | 0.0284955 |
| ZNF304 | 0.54814118 | hypo | chr19:57868195-57868196_+ | CDS | 1349 | 0.02127628 |
| SPG7 | 0.54816675 | hypo | chr16:89623783-89623784_+ | 3'UTR | 2700 | 0.00491635 |
| ATP5MF | 0.54923436 | hypo | chr7:99055943-99055944_- | 3'UTR | 350 | 0.03160681 |
| BLMH | 0.54936347 | hypo | chr17:28575980-28575981_- | 3'UTR | 1546 | 0.0187015 |
| ATG2B | 0.55000695 | hypo | chr14:96751982-96751983_- | 3'UTR | 6770 | 0.0017616 |
| ARL6IP5 | 0.55042419 | hypo | chr3:69153697-69153698_+ | CDS | 588 | 0.02657248 |
| SLC35C1 | 0.55057147 | hypo | chr11:45832857-45832858_+ | CDS | 1407 | 0.03824168 |
| GBP2 | 0.5508046 | hypo | chr1:89573915-89573916_- | CDS | 2031 | 0.00708805 |
| YWHAH | 0.55082257 | hypo | chr22:32352663-32352664_+ | CDS | 866 | 0.00302499 |
| EPN2 | 0.55090166 | hypo | chr17:19186378-19186379_+ | 5'UTR | 394 | 0.01449998 |
| C1QBP | 0.55098958 | hypo | chr17:5336261-5336262_- | 3'UTR | 999 | 0.00343234 |
| RABGEF1 | 0.5523858 | hypo | chr7:66274118-66274119_+ | CDS | 1477 | 0.01298699 |
| RRAGA | 0.5533293 | hypo | chr9:19049892-19049893_+ | CDS | 521 | 0.0141955 |
| IKZF5 | 0.5533307 | hypo | chr10:124753521-124753522_- | CDS | 1343 | 0.03581254 |
| SNW1 | 0.55345163 | hypo | chr14:78184363-78184364_- | 3'UTR | 1704 | 0.01178663 |
| ATF1 | 0.5534742 | hypo | chr12:51213778-51213779_+ | 3'UTR | 1340 | 0.0214385 |
| ATL3 | 0.55365352 | hypo | chr11:63398877-63398878_- | CDS | 1567 | 0.01334971 |
| EXT2 | 0.55381573 | hypo | chr11:44266178-44266179_+ | 3'UTR | 2653 | 0.0399972 |
| LAMC1 | 0.55403674 | hypo | chr1:183111957-183111958_+ | 3'UTR | 5119 | 0.0171745 |
| INTS10 | 0.55437372 | hypo | chr8:19709335-19709336_+ | 3'UTR | 2274 | 0.00136898 |
| CLASP1 | 0.55444941 | hypo | chr2:122097700-122097701_- | 3'UTR | 5742 | 0.03069785 |
| PLOD1 | 0.5553337 | hypo | chr1:12035094-12035095_+ | 3'UTR | 2526 | 0.03577131 |
| DNAAF5 | 0.55608182 | hypo | chr7:825362-825363_+ | 3'UTR | 2660 | 0.00283411 |
| VIM | 0.55629346 | hypo | chr10:17278339-17278340_+ | CDS | 1782 | 0.00914143 |
| POLR3B | 0.55635717 | hypo | chr12:106903496-106903497_+ | 3'UTR | 3793 | 0.02891437 |
| PIP4K2B | 0.55644505 | hypo | chr17:36924071-36924072_- | 3'UTR | 3603 | 0.00780003 |
| CANT1 | 0.5565083 | hypo | chr17:76989580-76989581_- | 3'UTR | 1556 | 0.00459297 |
| SRRD | 0.55652956 | hypo | chr22:26887743-26887744_+ | 3'UTR | 1132 | 0.02938541 |
| ZNF148 | 0.55654169 | hypo | chr3:124951564-124951565_- | CDS | 2490 | 0.00598864 |
| RAB32 | 0.55663579 | hypo | chr6:146875682-146875683_+ | CDS | 799 | 0.0441347 |
| SERPINB6 | 0.55704345 | hypo | chr6:2948740-2948741_- | CDS | 1124 | 0.01059258 |
| NAA50 | 0.5574273 | hypo | chr3:113439630-113439631_- | 3'UTR | 1835 | 0.03443837 |
| DDX19A | 0.55749914 | hypo | chr16:70406805-70406806_+ | 3'UTR | 2642 | 0.04256543 |
| EBLN3P | 0.55801224 | hypo | chr9:37087638-37087639_+ | 1615 | 1615 | 0.00495997 |
| AVL9 | 0.55813009 | hypo | chr7:32598997-32598998_+ | CDS | 1420 | 0.02995385 |
| VTA1 | 0.55860125 | hypo | chr6:142539824-142539825_+ | 3'UTR | 993 | 0.00154462 |
| SOD1 | 0.5586992 | hypo | chr21:33039602-33039603_+ | CDS | 419 | 0.01019431 |
| CACUL1 | 0.55887375 | hypo | chr10:120445601-120445602_- | 3'UTR | 1355 | 0.01348694 |
| CTSC | 0.55899606 | hypo | chr11:88027285-88027286_- | CDS | 1394 | 0.00844924 |
| MAP3K20 | 0.55909652 | hypo | chr2:174131353-174131354_+ | CDS | 2478 | 0.03459853 |
| ENG | 0.55927778 | hypo | chr9:130577481-130577482_- | 3'UTR | 2873 | 0.0028852 |
| PLEKHO1 | 0.5593084 | hypo | chr1:150131814-150131815_+ | 3'UTR | 1668 | 0.03353421 |
| DUSP3 | 0.55935807 | hypo | chr17:41845774-41845775_- | 3'UTR | 1812 | 0.04350152 |
| CITED2 | 0.55943533 | hypo | chr6:139694146-139694147_- | 3'UTR | 1024 | 0.00526789 |
| UBE2Q1 | 0.55951445 | hypo | chr1:154522776-154522777_- | 3'UTR | 1496 | 0.00945571 |
| PXMP2 | 0.55973038 | hypo | chr12:133281287-133281288_+ | 3'UTR | 667 | 0.00593379 |
| PFN2 | 0.56004425 | hypo | chr3:149683234-149683235_- | 3'UTR | 1561 | 0.02419802 |
| BLCAP | 0.56028515 | hypo | chr20:36145958-36145959_- | 3'UTR | 1952 | 0.02211594 |
| UVRAG | 0.56108348 | hypo | chr11:75852357-75852358_+ | CDS | 2241 | 0.03670551 |
| SLC31A2 | 0.5612588 | hypo | chr9:115925488-115925489_+ | 3'UTR | 840 | 0.00850956 |
| CAMLG | 0.56141151 | hypo | chr5:134077080-134077081_+ | CDS | 641 | 0.01852719 |
| DYNLRB1 | 0.56143816 | hypo | chr20:33114139-33114140_+ | CDS | 145 | 0.0347178 |
| KIAA0930 | 0.5617757 | hypo | chr22:45590765-45590766_- | 3'UTR | 3643 | 0.02262599 |
| USP47 | 0.56190861 | hypo | chr11:11964431-11964432_+ | CDS | 3422 | 0.04135669 |
| CENPX | 0.56191829 | hypo | chr17:79976956-79976957_- | 3'UTR | 417 | 0.04521081 |
| EBPL | 0.56234161 | hypo | chr13:50235086-50235087_- | 3'UTR | 700 | 0.02196501 |
| MRPL44 | 0.56306704 | hypo | chr2:224824510-224824511_+ | CDS | 508 | 0.03735933 |
| RNASEH1 | 0.56314631 | hypo | chr2:3593389-3593390_- | CDS | 956 | 0.00980541 |
| TMEM184C | 0.56349955 | hypo | chr4:148555585-148555586_+ | CDS | 1886 | 0.04326357 |
| ARL6IP5 | 0.56352894 | hypo | chr3:69153656-69153657_+ | CDS | 547 | 0.02111915 |
| MAGEF1 | 0.56369127 | hypo | chr3:184429207-184429208_- | CDS | 628 | 0.02151282 |
| CSE1L | 0.5639261 | hypo | chr20:47713275-47713276_+ | 3'UTR | 3405 | 0.02287799 |
| S1PR3 | 0.5640505 | hypo | chr9:91617429-91617430_+ | 3'UTR | 1748 | 0.01374512 |
| ZMPSTE24 | 0.56422128 | hypo | chr1:40758365-40758366_+ | 3'UTR | 1674 | 0.00318664 |
| DBN1 | 0.56448947 | hypo | chr5:176883964-176883965_- | 3'UTR | 2590 | 0.02130397 |
| AZI2 | 0.56459278 | hypo | chr3:28365500-28365501_- | 3'UTR | 1742 | 0.00949807 |
| CCDC127 | 0.56484653 | hypo | chr5:205831-205832_- | CDS | 462 | 0.02766418 |
| SPTLC2 | 0.56496159 | hypo | chr14:77978519-77978520_- | 3'UTR | 1983 | 0.00206793 |
| ZC3H11A | 0.56497917 | hypo | chr1:203822945-203822946_+ | 3'UTR | 4553 | 0.02513529 |
| EEF1E1 | 0.56528335 | hypo | chr6:8080147-8080148_- | CDS | 526 | 0.00199182 |
| DNAJA1 | 0.56551883 | hypo | chr9:33038861-33038862_+ | CDS | 1345 | 0.01309652 |
| MRPL36 | 0.5660898 | hypo | chr5:1798846-1798847_- | CDS | 265 | 0.00893369 |
| EDF1 | 0.56616376 | hypo | chr9:139756734-139756735_- | CDS | 497 | 0.04862203 |
| DDX23 | 0.56655983 | hypo | chr12:49223781-49223782_- | 3'UTR | 3023 | 0.00315391 |
| FAM126A | 0.56667362 | hypo | chr7:22985549-22985550_- | CDS | 1478 | 0.00909561 |
| PSMA2 | 0.56685989 | hypo | chr7:42957172-42957173_- | CDS | 752 | 0.04376446 |
| UBC | 0.56705282 | hypo | chr12:125397571-125397572_- | CDS | 1203 | 0.0249098 |
| WLS | 0.56718534 | hypo | chr1:68591768-68591769_- | 3'UTR | 1739 | 0.0040567 |
| SRA1 | 0.56720333 | hypo | chr5:139929854-139929855_- | 3'UTR | 1060 | 0.00153095 |
| PLOD1 | 0.5672455 | hypo | chr1:12035318-12035319_+ | 3'UTR | 2750 | 0.04097012 |
| XRCC5 | 0.56781468 | hypo | chr2:216981523-216981524_+ | CDS | 366 | 0.00463194 |
| LAS1L | 0.56813324 | hypo | chrX:64737982-64737983_- | CDS | 1901 | 0.00156245 |
| GUK1 | 0.56864814 | hypo | chr1:228336615-228336616_+ | 3'UTR | 863 | 0.00286931 |
| PPP2R3A | 0.56930192 | hypo | chr3:135722079-135722080_+ | CDS | 2301 | 0.0397308 |
| RAC1 | 0.56933788 | hypo | chr7:6443271-6443272_+ | 3'UTR | 2071 | 0.00442454 |
| RBPJ | 0.56941005 | hypo | chr4:26432701-26432702_+ | 3'UTR | 1758 | 0.01665656 |
| BTBD6 | 0.56941178 | hypo | chr14:105716452-105716453_+ | CDS | 1009 | 0.01100357 |
| TWISTNB | 0.56998109 | hypo | chr7:19735419-19735420_- | 3'UTR | 3556 | 0.02026917 |
| PAG1 | 0.5708917 | hypo | chr8:81887914-81887915_- | 3'UTR | 2873 | 0.01556684 |
| SACS | 0.57090819 | hypo | chr13:23909153-23909154_- | CDS | 9475 | 0.04070193 |
| SPG7 | 0.57097385 | hypo | chr16:89623758-89623759_+ | 3'UTR | 2675 | 0.00705308 |
| SSR4 | 0.57098369 | hypo | chrX:153063544-153063545_+ | CDS | 544 | 0.02593778 |
| HSP90AB1 | 0.57100366 | hypo | chr6:44220887-44220888_+ | CDS | 2016 | 0.02930116 |
| ZKSCAN8 | 0.57205622 | hypo | chr6:28121226-28121227_+ | CDS | 1380 | 0.00792738 |
| BID | 0.57238689 | hypo | chr22:18218200-18218201_- | 3'UTR | 1223 | 0.03118718 |
| MYO10 | 0.57248054 | hypo | chr5:16666562-16666563_- | 3'UTR | 6882 | 0.01574959 |
| EPDR1 | 0.57256137 | hypo | chr7:37990232-37990233_+ | 3'UTR | 1288 | 0.00041389 |
| IDI1 | 0.57311223 | hypo | chr10:1086661-1086662_- | 3'UTR | 1682 | 0.02892466 |
| CHAMP1 | 0.57318367 | hypo | chr13:115091181-115091182_+ | CDS | 2348 | 0.0011439 |
| EXOG | 0.573514 | hypo | chr3:38565635-38565636_+ | CDS | 917 | 0.04348076 |
| SLC25A36 | 0.57351589 | hypo | chr3:140695398-140695399_+ | 3'UTR | 1274 | 0.00895713 |
| NDUFV3 | 0.57377211 | hypo | chr21:44324304-44324305_+ | CDS | 1207 | 0.00330721 |
| CALR | 0.57383586 | hypo | chr19:13054372-13054373_+ | CDS | 1062 | 0.02060423 |
| NDUFS5 | 0.5739456 | hypo | chr1:39494409-39494410_+ | CDS | 119 | 0.01216311 |
| RNF169 | 0.57426012 | hypo | chr11:74553326-74553327_+ | 3'UTR | 7691 | 0.00200813 |
| LSM1 | 0.57426286 | hypo | chr8:38021128-38021129_- | 3'UTR | 870 | 0.04127864 |
| ACLY | 0.57430575 | hypo | chr17:40024028-40024029_- | 3'UTR | 3502 | 0.03633284 |
| VTA1 | 0.57460469 | hypo | chr6:142539894-142539895_+ | 3'UTR | 1063 | 0.01145859 |
| ZNF644 | 0.57462565 | hypo | chr1:91406302-91406303_- | CDS | 890 | 0.00202272 |
| CTSB | 0.57517412 | hypo | chr8:11702320-11702321_- | 3'UTR | 1507 | 0.01482424 |
| AKAP13 | 0.57521894 | hypo | chr15:86292338-86292339_+ | 3'UTR | 13078 | 0.01892239 |
| NUCKS1 | 0.57550004 | hypo | chr1:205689704-205689705_- | CDS | 576 | 0.04353818 |
| CAV1 | 0.57597757 | hypo | chr7:116199344-116199345_+ | 3'UTR | 818 | 0.00642968 |
| MLLT3 | 0.5760176 | hypo | chr9:20414204-20414205_- | CDS | 925 | 0.02583191 |
| EXT2 | 0.57610178 | hypo | chr11:44129566-44129567_+ | CDS | 459 | 0.02268949 |
| SLC20A2 | 0.57676178 | hypo | chr8:42330106-42330107_- | 5'UTR | 126 | 0.01238702 |
| TIMM22 | 0.57706744 | hypo | chr17:904430-904431_+ | 3'UTR | 937 | 0.01628009 |
| LAMTOR2 | 0.57732736 | hypo | chr1:156025184-156025185_+ | CDS | 363 | 0.01804144 |
| RAB8A | 0.57744046 | hypo | chr19:16244177-16244178_+ | 3'UTR | 1700 | 0.00019775 |
| DDB1 | 0.57751372 | hypo | chr11:61067418-61067419_- | 3'UTR | 3855 | 0.04766851 |
| AHSA1 | 0.57760707 | hypo | chr14:77935558-77935559_+ | CDS | 1080 | 0.0077709 |
| RAB8B | 0.57777536 | hypo | chr15:63556033-63556034_+ | 3'UTR | 935 | 0.00331994 |
| RAB8A | 0.57779252 | hypo | chr19:16244060-16244061_+ | 3'UTR | 1583 | 0.04792855 |
| SPOP | 0.57811766 | hypo | chr17:47677408-47677409_- | 3'UTR | 1752 | 0.00442721 |
| ZBTB1 | 0.57812807 | hypo | chr14:64989583-64989584_+ | CDS | 1752 | 0.04038188 |
| NXT1 | 0.57840177 | hypo | chr20:23335211-23335212_+ | 3'UTR | 920 | 0.00130458 |
| ZNF266 | 0.57875684 | hypo | chr19:9523752-9523753_- | 3'UTR | 3087 | 0.02726478 |
| CDC37 | 0.5788619 | hypo | chr19:10505785-10505786_- | CDS | 752 | 0.02085943 |
| GCC1 | 0.57888033 | hypo | chr7:127224772-127224773_- | CDS | 881 | 0.00014464 |
| NSDHL | 0.57891521 | hypo | chrX:152037663-152037664_+ | 3'UTR | 1319 | 0.04814679 |
| RPL35A | 0.57904514 | hypo | chr3:197680902-197680903_+ | CDS | 257 | 0.04339981 |
| CAPZA1 | 0.57922005 | hypo | chr1:113213229-113213230_+ | 3'UTR | 1728 | 0.01064205 |
| DNAJB4 | 0.57953764 | hypo | chr1:78482871-78482872_+ | 3'UTR | 2237 | 0.02913948 |
| PRRC2C | 0.57977729 | hypo | chr1:171501723-171501724_+ | CDS | 1756 | 0.00801994 |
| TOR1B | 0.5799995 | hypo | chr9:132571818-132571819_+ | CDS | 1037 | 0.01553131 |
| LPIN3 | 0.58002311 | hypo | chr20:39989043-39989044_+ | 3'UTR | 4184 | 0.02560184 |
| MSMO1 | 0.580143 | hypo | chr4:166263035-166263036_+ | CDS | 949 | 0.01758907 |
| PTRH2 | 0.58084981 | hypo | chr17:57775021-57775022_- | CDS | 545 | 0.00140862 |
| RGS2 | 0.58088443 | hypo | chr1:192780835-192780836_+ | 3'UTR | 778 | 0.0123437 |
| RB1CC1 | 0.58091786 | hypo | chr8:53570070-53570071_- | CDS | 2844 | 0.00542252 |
| MRPS21 | 0.58091895 | hypo | chr1:150280510-150280511_+ | CDS | 201 | 0.01331011 |
| CTSB | 0.58103972 | hypo | chr8:11702513-11702514_- | 3'UTR | 1314 | 0.02737822 |
| GNA12 | 0.5810581 | hypo | chr7:2768443-2768444_- | 3'UTR | 3680 | 0.0190102 |
| TM2D2 | 0.58131572 | hypo | chr8:38848982-38848983_- | CDS | 595 | 0.01949685 |
| SLAIN2 | 0.58132844 | hypo | chr4:48424103-48424104_+ | 3'UTR | 1899 | 0.03002119 |
| COPS2 | 0.58136225 | hypo | chr15:49419966-49419967_- | 3'UTR | 1611 | 0.02704813 |
| TRAPPC12 | 0.58160933 | hypo | chr2:3392028-3392029_+ | CDS | 840 | 0.03584169 |
| MXD4 | 0.58167679 | hypo | chr4:2252421-2252422_- | CDS | 510 | 0.02065327 |
| SEC61A1 | 0.58281495 | hypo | chr3:127789027-127789028_+ | 3'UTR | 2137 | 0.01209525 |
| EPOR | 0.58287987 | hypo | chr19:11488657-11488658_- | 3'UTR | 1635 | 0.00108375 |
| HSP90B1 | 0.58306594 | hypo | chr12:104340699-104340700_+ | CDS | 2424 | 0.00832071 |
| FBXO31 | 0.5832668 | hypo | chr16:87364667-87364668_- | 3'UTR | 1894 | 0.00773574 |
| TCF20 | 0.5834871 | hypo | chr22:42606164-42606165_- | CDS | 5283 | 0.02773348 |
| BOD1L1 | 0.58367227 | hypo | chr4:13616250-13616251_- | CDS | 859 | 0.00085211 |
| LSM10 | 0.58383618 | hypo | chr1:36859599-36859600_- | CDS | 347 | 0.00700238 |
| C16orf72 | 0.58403799 | hypo | chr16:9210680-9210681_+ | CDS | 1104 | 0.03241526 |
| ARL6IP5 | 0.58427966 | hypo | chr3:69153873-69153874_+ | 3'UTR | 764 | 0.02238644 |
| DCTN2 | 0.58445733 | hypo | chr12:57924361-57924362_- | 3'UTR | 1584 | 0.02891365 |
| HSBP1 | 0.58448331 | hypo | chr16:83845245-83845246_+ | 3'UTR | 627 | 0.04652422 |
| HIST1H3B | 0.58465517 | hypo | chr6:26031917-26031918_- | CDS | 370 | 0.02797242 |
| SNW1 | 0.5846668 | hypo | chr14:78184514-78184515_- | CDS | 1553 | 0.02369515 |
| RIPK1 | 0.58474372 | hypo | chr6:3106102-3106103_+ | CDS | 1672 | 0.02616012 |
| CCDC93 | 0.58485196 | hypo | chr2:118677412-118677413_- | 3'UTR | 2569 | 0.00841496 |
| VOPP1 | 0.58503377 | hypo | chr7:55539059-55539060_- | 3'UTR | 2202 | 0.02043521 |
| TPM2 | 0.58520715 | hypo | chr9:35689773-35689774_- | CDS | 279 | 0.03250005 |
| TRIP12 | 0.58527286 | hypo | chr2:230632130-230632131_- | 3'UTR | 6296 | 0.02643332 |
| MCFD2 | 0.58542125 | hypo | chr2:47132420-47132421_- | 3'UTR | 759 | 0.01496185 |
| GPX8 | 0.58581112 | hypo | chr5:54460416-54460417_+ | 3'UTR | 1075 | 0.02997153 |
| RANBP2 | 0.58582114 | hypo | chr2:109399208-109399209_+ | CDS | 9385 | 0.01945666 |
| UTP3 | 0.58588835 | hypo | chr4:71554994-71554995_+ | CDS | 799 | 0.03804569 |
| TUBB | 0.58614629 | hypo | chr6:30691446-30691447_+ | CDS | 913 | 0.04050952 |
| PRPF4 | 0.5864615 | hypo | chr9:116054701-116054702_+ | 3'UTR | 2440 | 0.03151109 |
| COPS6 | 0.58675319 | hypo | chr7:99689436-99689437_+ | 3'UTR | 1039 | 0.01527246 |
| SRPK1 | 0.58744153 | hypo | chr6:35801458-35801459_- | 3'UTR | 3713 | 0.02190317 |
| TMEM115 | 0.5874654 | hypo | chr3:50392592-50392593_- | 3'UTR | 1681 | 0.04797727 |
| LRRC59 | 0.58756157 | hypo | chr17:48458991-48458992_- | 3'UTR | 2516 | 0.04334959 |
| CAPN7 | 0.58780676 | hypo | chr3:15292700-15292701_+ | CDS | 2645 | 0.00747876 |
| SGSH | 0.58796846 | hypo | chr17:78184009-78184010_- | 3'UTR | 1836 | 0.03633322 |
| HIST1H1C | 0.58802624 | hypo | chr6:26056196-26056197_- | CDS | 502 | 0.02792759 |
| TM2D2 | 0.58803551 | hypo | chr8:38848425-38848426_- | 3'UTR | 1152 | 0.00925726 |
| GALNT11 | 0.58837338 | hypo | chr7:151819008-151819009_+ | 3'UTR | 2322 | 0.03329483 |
| ZNF79 | 0.58842254 | hypo | chr9:130207482-130207483_+ | 3'UTR | 1917 | 0.00111684 |
| ERLIN1 | 0.58842339 | hypo | chr10:101911830-101911831_- | 3'UTR | 1210 | 0.02698029 |
| XXYLT1 | 0.58861822 | hypo | chr3:194790064-194790065_- | 3'UTR | 1668 | 0.01198583 |
| ZC3H13 | 0.58871405 | hypo | chr13:46549888-46549889_- | CDS | 2086 | 0.01323341 |
| TPM1 | 0.58891322 | hypo | chr15:63336283-63336284_+ | CDS | 363 | 0.01122824 |
| PRPSAP1 | 0.58897063 | hypo | chr17:74307641-74307642_- | CDS | 1584 | 0.00243779 |
| GUCD1 | 0.5890044 | hypo | chr22:24936424-24936425_- | 3'UTR | 3587 | 0.00942799 |
| MED19 | 0.58903043 | hypo | chr11:57471427-57471428_- | 3'UTR | 1286 | 0.04522654 |
| KCTD1 | 0.58907851 | hypo | chr18:24035586-24035587_- | 3'UTR | 2717 | 0.01672235 |
| DNAJC2 | 0.5894096 | hypo | chr7:102953015-102953016_- | CDS | 2116 | 0.01098219 |
| NPLOC4 | 0.58943134 | hypo | chr17:79524869-79524870_- | 3'UTR | 3464 | 0.01986198 |
| WIPI2 | 0.58968018 | hypo | chr7:5270540-5270541_+ | CDS | 1543 | 0.01467135 |
| RPS24 | 0.58983447 | hypo | chr10:79800432-79800433_+ | 3'UTR | 592 | 0.00627876 |
| ATMIN | 0.58988934 | hypo | chr16:81078461-81078462_+ | CDS | 2376 | 0.00726315 |
| KANK2 | 0.58996053 | hypo | chr19:11276626-11276627_- | 3'UTR | 3265 | 0.02691954 |
| TOMM5 | 0.59030915 | hypo | chr9:37588763-37588764_- | 3'UTR | 552 | 0.01933252 |
| SEC61A1 | 0.59035388 | hypo | chr3:127789069-127789070_+ | 3'UTR | 2179 | 0.01374296 |
| ZNF133 | 0.59043556 | hypo | chr20:18297474-18297475_+ | 3'UTR | 2372 | 0.00887088 |
| NFKBIE | 0.59133681 | hypo | chr6:44226524-44226525_- | 3'UTR | 1958 | 0.0162209 |
| GLT8D1 | 0.59135713 | hypo | chr3:52728793-52728794_- | 3'UTR | 1413 | 0.00028906 |
| RUSC2 | 0.59143008 | hypo | chr9:35546697-35546698_+ | CDS | 611 | 0.03456199 |
| PSME4 | 0.59147169 | hypo | chr2:54092159-54092160_- | 3'UTR | 6142 | 0.0371686 |
| HMOX2 | 0.59165022 | hypo | chr16:4557843-4557844_+ | CDS | 523 | 0.03856607 |
| AZI2 | 0.59165405 | hypo | chr3:28365674-28365675_- | CDS | 1568 | 0.01858638 |
| FDFT1 | 0.59201544 | hypo | chr8:11696111-11696112_+ | CDS | 1469 | 0.00926899 |
| AHNAK2 | 0.59250064 | hypo | chr14:105403776-105403777_- | 3'UTR | 18130 | 0.00122169 |
| TRAM2 | 0.59252988 | hypo | chr6:52362482-52362483_- | 3'UTR | 6773 | 0.02041052 |
| TAF11 | 0.59254051 | hypo | chr6:34846408-34846409_- | CDS | 707 | 0.01745869 |
| SMAD2 | 0.59265615 | hypo | chr18:45368158-45368159_- | 3'UTR | 1842 | 0.03642127 |
| GALNT2 | 0.59274388 | hypo | chr1:230415795-230415796_+ | 3'UTR | 2393 | 0.01831104 |
| POLR1B | 0.59293814 | hypo | chr2:113332869-113332870_+ | CDS | 3551 | 0.0359113 |
| FSTL1 | 0.59294764 | hypo | chr3:120113822-120113823_- | 3'UTR | 2998 | 0.01206045 |
| SREK1 | 0.59306088 | hypo | chr5:65466675-65466676_+ | CDS | 1543 | 0.00906394 |
| MAP3K7 | 0.59344183 | hypo | chr6:91226307-91226308_- | CDS | 1921 | 0.01258719 |
| CAMSAP2 | 0.59347271 | hypo | chr1:200827211-200827212_+ | 3'UTR | 4731 | 0.00060897 |
| CSDE1 | 0.5934993 | hypo | chr1:115267915-115267916_- | CDS | 2338 | 0.04252717 |
| TMEM9B | 0.59370661 | hypo | chr11:8969101-8969102_- | 3'UTR | 1821 | 0.02781705 |
| CCM2 | 0.59371134 | hypo | chr7:45115659-45115660_+ | 3'UTR | 1472 | 0.03710777 |
| EIF3A | 0.59396052 | hypo | chr10:120817508-120817509_- | CDS | 2143 | 0.03317502 |
| TMEM203 | 0.59421093 | hypo | chr9:140098689-140098690_- | 3'UTR | 1410 | 0.02617748 |
| ETF1 | 0.59442732 | hypo | chr5:137843285-137843286_- | 3'UTR | 2243 | 0.01387453 |
| HDLBP | 0.5947261 | hypo | chr2:242192834-242192835_- | CDS | 1493 | 0.02442862 |
| MORF4L2 | 0.59491147 | hypo | chrX:102931919-102931920_- | CDS | 441 | 0.02141749 |
| PQLC1 | 0.59496417 | hypo | chr18:77663231-77663232_- | 3'UTR | 1731 | 0.04254495 |
| BORCS8 | 0.59516615 | hypo | chr19:19287919-19287920_- | 3'UTR | 1225 | 0.01724678 |
| EFTUD2 | 0.59530942 | hypo | chr17:42928512-42928513_- | 3'UTR | 3128 | 0.01308158 |
| PCF11 | 0.59546284 | hypo | chr11:82895790-82895791_+ | CDS | 4867 | 0.03960758 |
| FOXK2 | 0.59580087 | hypo | chr17:80559915-80559916_+ | 3'UTR | 2694 | 0.03730886 |
| BSDC1 | 0.59591364 | hypo | chr1:32834138-32834139_- | CDS | 1266 | 0.01210284 |
| RPL3 | 0.59611007 | hypo | chr22:39713589-39713590_- | CDS | 266 | 0.03421331 |
| TIMM23 | 0.59620966 | hypo | chr10:51592443-51592444_- | 3'UTR | 861 | 0.01719574 |
| TOMM6 | 0.59627387 | hypo | chr6:41757305-41757306_+ | 3'UTR | 525 | 0.03570923 |
| ANKIB1 | 0.59673031 | hypo | chr7:92028215-92028216_+ | CDS | 3598 | 0.01488461 |
| SMIM26 | 0.59690494 | hypo | chr20:18548221-18548222_+ | CDS | 158 | 0.00707177 |
| PREP | 0.59726754 | hypo | chr6:105726017-105726018_- | CDS | 2366 | 0.02022828 |
| GLT8D1 | 0.59760054 | hypo | chr3:52728983-52728984_- | CDS | 1223 | 0.00847217 |
| SACS | 0.59767053 | hypo | chr13:23904845-23904846_- | CDS | 13783 | 0.0092536 |
| AFG3L2 | 0.59797664 | hypo | chr18:12329651-12329652_- | CDS | 2499 | 0.02979252 |
| NRDC | 0.59822105 | hypo | chr1:52344126-52344127_- | CDS | 482 | 0.00776994 |
| GUK1 | 0.59869722 | hypo | chr1:228336539-228336540_+ | CDS | 787 | 0.01903595 |
| TMEM43 | 0.59875027 | hypo | chr3:14183786-14183787_+ | 3'UTR | 1948 | 0.01030688 |
| PPP1R7 | 0.59909563 | hypo | chr2:242092972-242092973_+ | CDS | 972 | 0.04327105 |
| CALU | 0.5991139 | hypo | chr7:128407704-128407705_+ | CDS | 990 | 0.03440779 |
| PELO | 0.59951239 | hypo | chr5:52097695-52097696_+ | 3'UTR | 2164 | 0.03442367 |
| MCFD2 | 0.60020895 | hypo | chr2:47132543-47132544_- | 3'UTR | 636 | 0.04391504 |
| MAVS | 0.60041875 | hypo | chr20:3854521-3854522_+ | 3'UTR | 9522 | 0.00705153 |
| ZNF394 | 0.60055084 | hypo | chr7:99091733-99091734_- | CDS | 1291 | 0.04201465 |
| ZBED6 | 0.60081077 | hypo | chr1:203769433-203769434_+ | CDS | 2783 | 0.00696351 |
| HPS6 | 0.60084075 | hypo | chr10:103827578-103827579_+ | 3'UTR | 2455 | 0.00587896 |
| HSBP1 | 0.60151829 | hypo | chr16:83841652-83841653_+ | 5'UTR | 145 | 0.00107776 |
| C11orf68 | 0.6016984 | hypo | chr11:65684416-65684417_- | 3'UTR | 1423 | 0.03674832 |
| SERBP1 | 0.60239939 | hypo | chr1:67878874-67878875_- | 3'UTR | 1381 | 0.00970304 |
| TP53BP1 | 0.60255993 | hypo | chr15:43748382-43748383_- | CDS | 2550 | 0.03361339 |
| SPTLC2 | 0.60272315 | hypo | chr14:77978637-77978638_- | CDS | 1865 | 0.01780611 |
| POLR1D | 0.60277706 | hypo | chr13:28239912-28239913_+ | CDS | 396 | 0.00130382 |
| NCBP2-AS2 | 0.60293565 | hypo | chr3:196670132-196670133_+ | 3'UTR | 639 | 0.00948746 |
| MARK3 | 0.60299052 | hypo | chr14:103969322-103969323_+ | CDS | 2686 | 0.01476367 |
| ATP5IF1 | 0.60329996 | hypo | chr1:28564541-28564542_+ | 3'UTR | 466 | 0.0116383 |
| TCTN3 | 0.60345497 | hypo | chr10:97423457-97423458_- | 3'UTR | 2433 | 0.04021147 |
| FOXO1 | 0.6035274 | hypo | chr13:41134395-41134396_- | CDS | 1660 | 0.0042241 |
| SPG7 | 0.60370367 | hypo | chr16:89623917-89623918_+ | 3'UTR | 2834 | 0.01015484 |
| VCP | 0.60390187 | hypo | chr9:35059158-35059159_- | CDS | 2450 | 0.03555636 |
| WDR92 | 0.60400226 | hypo | chr2:68357968-68357969_- | 3'UTR | 1558 | 0.00414172 |
| CRKL | 0.60406023 | hypo | chr22:21307518-21307519_+ | 3'UTR | 4806 | 0.03426116 |
| JPT2 | 0.60408728 | hypo | chr16:1749354-1749355_+ | 3'UTR | 964 | 0.02553215 |
| NECAP2 | 0.60425021 | hypo | chr1:16785693-16785694_+ | 3'UTR | 1114 | 0.01542959 |
| COL4A1 | 0.60429912 | hypo | chr13:110802521-110802522_- | 3'UTR | 5327 | 0.01122751 |
| PCYOX1 | 0.60429997 | hypo | chr2:70504581-70504582_+ | 3'UTR | 1603 | 0.00069444 |
| TRIP12 | 0.6044211 | hypo | chr2:230725135-230725136_- | CDS | 388 | 0.03540358 |
| RPLP0 | 0.60460374 | hypo | chr12:120637169-120637170_- | CDS | 349 | 0.03317721 |
| POLR3H | 0.60503334 | hypo | chr22:41924947-41924948_- | 3'UTR | 1035 | 0.02012902 |
| MFSD11 | 0.605035 | hypo | chr17:74774359-74774360_+ | CDS | 2127 | 0.01977995 |
| TRIP6 | 0.60507079 | hypo | chr7:100470992-100470993_+ | 3'UTR | 1668 | 0.01019467 |
| RALB | 0.60512585 | hypo | chr2:121050873-121050874_+ | 3'UTR | 848 | 0.03956061 |
| MCFD2 | 0.6051981 | hypo | chr2:47132721-47132722_- | CDS | 458 | 0.0105944 |
| ATXN2 | 0.60522208 | hypo | chr12:111890307-111890308_- | 3'UTR | 4411 | 0.0178611 |
| ANXA2 | 0.60533779 | hypo | chr15:60639747-60639748_- | 3'UTR | 1227 | 0.015608 |
| RNF8 | 0.60542328 | hypo | chr6:37358652-37358653_+ | 3'UTR | 1758 | 0.00113172 |
| STXBP1 | 0.60610426 | hypo | chr9:130435517-130435518_+ | CDS | 1284 | 0.0082529 |
| IL27RA | 0.60627259 | hypo | chr19:14163195-14163196_+ | 3'UTR | 2137 | 0.01409258 |
| ERP29 | 0.60630414 | hypo | chr12:112460406-112460407_+ | CDS | 854 | 0.01487115 |
| KDM3B | 0.60643733 | hypo | chr5:137726994-137726995_+ | CDS | 1873 | 0.03320199 |
| SLC39A6 | 0.60654482 | hypo | chr18:33706431-33706432_- | CDS | 828 | 0.00336539 |
| ASXL1 | 0.6065884 | hypo | chr20:31023090-31023091_+ | CDS | 3007 | 0.0173776 |
| PA2G4 | 0.6067373 | hypo | chr12:56507506-56507507_+ | 3'UTR | 2438 | 0.04294862 |
| EEF2 | 0.60675578 | hypo | chr19:3976344-3976345_- | 3'UTR | 2866 | 0.03916883 |
| ENTPD4 | 0.60733993 | hypo | chr8:23294546-23294547_- | CDS | 1545 | 0.00213196 |
| INF2 | 0.60799087 | hypo | chr14:105170593-105170594_+ | 3'UTR | 1151 | 0.00535006 |
| ETF1 | 0.60817246 | hypo | chr5:137843839-137843840_- | 3'UTR | 1689 | 0.04239223 |
| ANKRD11 | 0.60820717 | hypo | chr16:89351882-89351883_- | CDS | 1524 | 0.01513346 |
| YY1AP1 | 0.60864786 | hypo | chr1:155629529-155629530_- | CDS | 2776 | 0.01648951 |
| USP4 | 0.60917075 | hypo | chr3:49315790-49315791_- | CDS | 2905 | 0.02180859 |
| LTBR | 0.60942894 | hypo | chr12:6500235-6500236_+ | 3'UTR | 1766 | 0.03627876 |
| ZNF384 | 0.60992967 | hypo | chr12:6776092-6776093_- | 3'UTR | 2790 | 0.00423062 |
| HMOX2 | 0.60995092 | hypo | chr16:4558086-4558087_+ | CDS | 766 | 0.02633954 |
| ZMYND8 | 0.61010251 | hypo | chr20:45839392-45839393_- | 3'UTR | 3798 | 0.00857406 |
| FAM32A | 0.61030021 | hypo | chr19:16302361-16302362_+ | 3'UTR | 976 | 0.0190678 |
| RTRAF | 0.61048342 | hypo | chr14:52471234-52471235_+ | CDS | 865 | 0.00877644 |
| PRSS23 | 0.61063223 | hypo | chr11:86519085-86519086_+ | CDS | 523 | 0.00394088 |
| IRS1 | 0.61101038 | hypo | chr2:227662928-227662929_- | CDS | 577 | 0.01790401 |
| RNMT | 0.61113373 | hypo | chr18:13731676-13731677_+ | CDS | 401 | 0.00947568 |
| RSPRY1 | 0.61119538 | hypo | chr16:57238540-57238541_+ | 5'UTR | 293 | 0.03778953 |
| TSSC4 | 0.61125346 | hypo | chr11:2424537-2424538_+ | CDS | 1165 | 0.01540198 |
| PWWP2A | 0.61149243 | hypo | chr5:159519971-159519972_- | CDS | 1741 | 0.04531372 |
| PLIN3 | 0.61228016 | hypo | chr19:4839276-4839277_- | CDS | 1408 | 0.02882999 |
| DHX16 | 0.61273253 | hypo | chr6:30638880-30638881_- | CDS | 542 | 0.01934305 |
| LRP10 | 0.61283377 | hypo | chr14:23348800-23348801_+ | 3'UTR | 4793 | 0.02274395 |
| ATG5 | 0.61293989 | hypo | chr6:106634393-106634394_- | 3'UTR | 1201 | 0.00552622 |
| ABHD15 | 0.61307921 | hypo | chr17:27888004-27888005_- | 3'UTR | 3038 | 0.00464394 |
| UNC45A | 0.61314364 | hypo | chr15:91496896-91496897_+ | CDS | 2825 | 0.01530015 |
| SPOP | 0.61363981 | hypo | chr17:47677435-47677436_- | 3'UTR | 1725 | 0.00541937 |
| SCAF8 | 0.61376875 | hypo | chr6:155153407-155153408_+ | CDS | 3217 | 0.02514827 |
| KIF3C | 0.61382332 | hypo | chr2:26203419-26203420_- | CDS | 2023 | 0.04764546 |
| LYN | 0.61382592 | hypo | chr8:56922948-56922949_+ | 3'UTR | 2100 | 0.00408204 |
| CAMK2G | 0.61413888 | hypo | chr10:75572443-75572444_- | 3'UTR | 3632 | 0.00454811 |
| EEF2 | 0.61432727 | hypo | chr19:3976396-3976397_- | 3'UTR | 2814 | 0.00413364 |
| TUG1 | 0.6145725 | hypo | chr22:31374552-31374553_+ | 6824 | 6824 | 0.04525089 |
| SDHB | 0.61484013 | hypo | chr1:17355164-17355165_- | CDS | 503 | 0.03154921 |
| HARS2 | 0.61491259 | hypo | chr5:140078625-140078626_+ | 3'UTR | 2232 | 0.00342958 |
| QSOX1 | 0.61533041 | hypo | chr1:180166053-180166054_+ | CDS | 2200 | 0.04034309 |
| C19orf48 | 0.61551117 | hypo | chr19:51301813-51301814_- | 5'UTR | 788 | 0.04429089 |
| UBL4A | 0.61553599 | hypo | chrX:153712636-153712637_- | 3'UTR | 1800 | 0.02394501 |
| THAP11 | 0.61569435 | hypo | chr16:67877775-67877776_+ | 3'UTR | 1563 | 0.03569682 |
| DLGAP4 | 0.61579174 | hypo | chr20:35155390-35155391_+ | CDS | 3419 | 0.02721523 |
| PPP2R5E | 0.61582887 | hypo | chr14:63842564-63842565_- | 3'UTR | 2171 | 0.00115746 |
| CD59 | 0.61596507 | hypo | chr11:33731764-33731765_- | CDS | 575 | 0.01034928 |
| SAR1A | 0.61613029 | hypo | chr10:71912149-71912150_- | 3'UTR | 796 | 0.01699257 |
| PDCD6 | 0.61633762 | hypo | chr5:314596-314597_+ | CDS | 642 | 0.01958124 |
| ARPC5 | 0.61642524 | hypo | chr1:183604729-183604730_- | CDS | 346 | 0.03184468 |
| FAM168A | 0.61656847 | hypo | chr11:73116272-73116273_- | 3'UTR | 2527 | 0.02681675 |
| MAP3K7 | 0.61676978 | hypo | chr6:91225879-91225880_- | 3'UTR | 2349 | 0.0345476 |
| NDST1 | 0.61678519 | hypo | chr5:149935540-149935541_+ | 3'UTR | 5797 | 0.00518167 |
| CCDC80 | 0.61694016 | hypo | chr3:112359401-112359402_- | 5'UTR | 588 | 0.02598666 |
| PSMA5 | 0.61697417 | hypo | chr1:109944641-109944642_- | CDS | 836 | 0.04847125 |
| PLK2 | 0.61697595 | hypo | chr5:57750161-57750162_- | 3'UTR | 2485 | 0.00352859 |
| C9orf78 | 0.61705063 | hypo | chr9:132589934-132589935_- | 3'UTR | 1446 | 0.02377566 |
| GEMIN6 | 0.61705944 | hypo | chr2:39009051-39009052_+ | 3'UTR | 637 | 0.03579042 |
| NPEPPS | 0.61706653 | hypo | chr17:45699405-45699406_+ | 3'UTR | 3102 | 0.00931131 |
| UBAC1 | 0.61725595 | hypo | chr9:138825262-138825263_- | CDS | 1418 | 0.01197639 |
| POLR1C | 0.61731847 | hypo | chr6:43489145-43489146_+ | 3'UTR | 1234 | 0.02310375 |
| ARHGAP29 | 0.61748696 | hypo | chr1:94639581-94639582_- | CDS | 3997 | 0.03804995 |
| TKT | 0.6175233 | hypo | chr3:53269079-53269080_- | CDS | 743 | 0.01720812 |
| FZD6 | 0.61753525 | hypo | chr8:104342176-104342177_+ | CDS | 2125 | 0.03684489 |
| DYNLL1 | 0.61760959 | hypo | chr12:120936114-120936115_+ | 3'UTR | 552 | 0.00649853 |
| LATS1 | 0.61805232 | hypo | chr6:150005243-150005244_- | CDS | 1528 | 0.04198889 |
| SHOC2 | 0.61805508 | hypo | chr10:112773007-112773008_+ | 3'UTR | 3529 | 0.03371553 |
| ZEB1 | 0.61808833 | hypo | chr10:31809279-31809280_+ | CDS | 1082 | 0.02519746 |
| MRFAP1L1 | 0.61841328 | hypo | chr4:6709625-6709626_- | 3'UTR | 1393 | 0.01104787 |
| IFNAR1 | 0.61843778 | hypo | chr21:34727975-34727976_+ | 3'UTR | 1941 | 0.04903326 |
| VPS33B | 0.61851582 | hypo | chr15:91542061-91542062_- | 3'UTR | 2368 | 0.00081953 |
| GLB1 | 0.61874532 | hypo | chr3:33038651-33038652_- | CDS | 2063 | 0.02907524 |
| C3orf38 | 0.61890975 | hypo | chr3:88205676-88205677_+ | CDS | 1191 | 0.00231862 |
| ARPC3 | 0.61901982 | hypo | chr12:110883309-110883310_- | CDS | 203 | 0.00658668 |
| NCL | 0.61902255 | hypo | chr2:232325228-232325229_- | CDS | 998 | 0.01674927 |
| MYO1E | 0.61911174 | hypo | chr15:59429434-59429435_- | 3'UTR | 3842 | 0.02101939 |
| PKM | 0.61919745 | hypo | chr15:72491470-72491471_- | 3'UTR | 2974 | 0.01523782 |
| LAMC1 | 0.61924479 | hypo | chr1:183105544-183105545_+ | CDS | 4395 | 0.00213285 |
| SUPT4H1 | 0.6193014 | hypo | chr17:56423531-56423532_- | 3'UTR | 531 | 0.01373469 |
| BCAR1 | 0.61957101 | hypo | chr16:75263897-75263898_- | CDS | 2547 | 0.0465433 |
| MAP3K7 | 0.61961385 | hypo | chr6:91226270-91226271_- | CDS | 1958 | 0.01330171 |
| FAM171A1 | 0.61968686 | hypo | chr10:15254250-15254251_- | 3'UTR | 3342 | 0.02917934 |
| DCAF5 | 0.61983775 | hypo | chr14:69520713-69520714_- | CDS | 2907 | 0.0070826 |
| C1orf43 | 0.61994539 | hypo | chr1:154179950-154179951_- | CDS | 1129 | 0.02514706 |
| SNIP1 | 0.62000028 | hypo | chr1:38006180-38006181_- | CDS | 617 | 0.04446586 |
| ANKRD17 | 0.62064203 | hypo | chr4:73964165-73964166_- | CDS | 4774 | 0.04747932 |
| KIAA0930 | 0.62098816 | hypo | chr22:45592518-45592519_- | 3'UTR | 1890 | 0.00210925 |
| YIF1A | 0.62133158 | hypo | chr11:66055337-66055338_- | CDS | 450 | 0.02279695 |
| CCT4 | 0.62158228 | hypo | chr2:62100200-62100201_- | CDS | 1224 | 0.04359631 |
| REEP5 | 0.62175899 | hypo | chr5:112222773-112222774_- | CDS | 494 | 0.03079591 |
| SLC39A6 | 0.62181976 | hypo | chr18:33706491-33706492_- | CDS | 768 | 0.00654826 |
| STK16 | 0.62195508 | hypo | chr2:220113524-220113525_+ | 3'UTR | 1333 | 0.04194199 |
| AGFG1 | 0.62204163 | hypo | chr2:228419221-228419222_+ | 3'UTR | 2015 | 0.01878436 |
| TRAPPC5 | 0.62223256 | hypo | chr19:7747539-7747540_+ | CDS | 641 | 0.0300836 |
| CSNK1D | 0.62268938 | hypo | chr17:80202627-80202628_- | 3'UTR | 1645 | 0.0131619 |
| MFSD5 | 0.62312674 | hypo | chr12:53648101-53648102_+ | 3'UTR | 1950 | 0.0042557 |
| IL1R1 | 0.6231958 | hypo | chr2:102793035-102793036_+ | CDS | 1854 | 0.02424652 |
| ARPC5L | 0.6235722 | hypo | chr9:127639332-127639333_+ | 3'UTR | 1839 | 0.00936171 |
| NOSIP | 0.62376959 | hypo | chr19:50063271-50063272_- | CDS | 153 | 0.01265511 |
| SCARB2 | 0.62407775 | hypo | chr4:77080049-77080050_- | 3'UTR | 4608 | 0.00358906 |
| PDE8A | 0.62416858 | hypo | chr15:85681271-85681272_+ | 3'UTR | 2816 | 0.00889606 |
| MTRNR2L2 | 0.62421464 | hypo | chr5:79946223-79946224_- | 5'UTR | 630 | 0.0067625 |
| CDK13 | 0.62432642 | hypo | chr7:40027346-40027347_+ | CDS | 1746 | 0.04070237 |
| DNAJC13 | 0.62437494 | hypo | chr3:132257140-132257141_+ | 3'UTR | 6811 | 0.01910721 |
| RBM25 | 0.62464603 | hypo | chr14:73570091-73570092_+ | CDS | 1246 | 0.00923858 |
| PLEC | 0.6248288 | hypo | chr8:144998388-144998389_- | CDS | 6288 | 0.02849493 |
| GAPDH | 0.62484767 | hypo | chr12:6646527-6646528_+ | CDS | 572 | 0.01772635 |
| HSP90AA1 | 0.62494634 | hypo | chr14:102551237-102551238_- | CDS | 1471 | 0.02611542 |
| FJX1 | 0.62495857 | hypo | chr11:35642096-35642097_+ | 3'UTR | 2362 | 0.03534503 |
| TTC3 | 0.62517939 | hypo | chr21:38573924-38573925_+ | 3'UTR | 6319 | 0.0296413 |
| PRDM4 | 0.62526216 | hypo | chr12:108128051-108128052_- | CDS | 2777 | 0.00755674 |
| RPP25L | 0.62535069 | hypo | chr9:34611012-34611013_- | CDS | 366 | 0.00232786 |
| PELO | 0.62552606 | hypo | chr5:52097318-52097319_+ | CDS | 1787 | 0.02926117 |
| RAP2A | 0.62556852 | hypo | chr13:98116594-98116595_+ | CDS | 700 | 0.00426188 |
| BAG3 | 0.62560354 | hypo | chr10:121436550-121436551_+ | CDS | 1790 | 0.00802345 |
| COQ6 | 0.62584798 | hypo | chr14:74429742-74429743_+ | 3'UTR | 1528 | 0.00361197 |
| YTHDF2 | 0.62597177 | hypo | chr1:29069282-29069283_+ | CDS | 763 | 0.00950009 |
| MAP1B | 0.62615835 | hypo | chr5:71491291-71491292_+ | CDS | 2373 | 0.04315463 |
| UXT | 0.62669662 | hypo | chrX:47511219-47511220_- | 3'UTR | 747 | 0.00402952 |
| VCAN | 0.62690847 | hypo | chr5:82876982-82876983_+ | 3'UTR | 11276 | 0.03001617 |
| B4GALT7 | 0.6272128 | hypo | chr5:177036808-177036809_+ | 3'UTR | 1189 | 0.04370572 |
| RBBP6 | 0.62744277 | hypo | chr16:24583365-24583366_+ | CDS | 6018 | 0.0491357 |
| TRRAP | 0.62748802 | hypo | chr7:98610670-98610671_+ | 3'UTR | 12481 | 0.01876876 |
| TRMT10C | 0.62780835 | hypo | chr3:101284143-101284144_+ | CDS | 698 | 0.04566424 |
| AK1 | 0.62807505 | hypo | chr9:130630119-130630120_- | 3'UTR | 904 | 0.00633132 |
| P3H1 | 0.6282164 | hypo | chr1:43212078-43212079_- | 3'UTR | 3031 | 0.02127936 |
| LMF2 | 0.62840468 | hypo | chr22:50941752-50941753_- | 3'UTR | 2221 | 0.01218283 |
| CETN2 | 0.62853759 | hypo | chrX:151995982-151995983_- | 3'UTR | 967 | 0.00409775 |
| GOLGA5 | 0.628926 | hypo | chr14:93275650-93275651_+ | CDS | 1034 | 0.04096164 |
| ASCC1 | 0.62902625 | hypo | chr10:73857001-73857002_- | 3'UTR | 1712 | 0.01047322 |
| EXT1 | 0.6291577 | hypo | chr8:119123288-119123289_- | 5'UTR | 769 | 0.00820485 |
| TUBB6 | 0.62921922 | hypo | chr18:12326450-12326451_+ | 3'UTR | 1896 | 0.03870928 |
| RILPL1 | 0.62943545 | hypo | chr12:123956956-123956957_- | 3'UTR | 1575 | 0.02482314 |
| BDNF | 0.62945706 | hypo | chr11:27679749-27679750_- | CDS | 748 | 0.02284881 |
| TGOLN2 | 0.62945887 | hypo | chr2:85554366-85554367_- | CDS | 821 | 0.02029684 |
| NCBP2-AS2 | 0.62952449 | hypo | chr3:196670237-196670238_+ | 3'UTR | 744 | 0.00315625 |
| PURB | 0.62961637 | hypo | chr7:44923631-44923632_- | 3'UTR | 1352 | 0.0463332 |
| SHOC2 | 0.62986403 | hypo | chr10:112724321-112724322_+ | CDS | 554 | 0.02483496 |
| ING5 | 0.6299729 | hypo | chr2:242664589-242664590_+ | 3'UTR | 913 | 0.04551519 |
| ELOA | 0.62999548 | hypo | chr1:24078002-24078003_+ | CDS | 1045 | 0.02542474 |
| SEL1L | 0.63001278 | hypo | chr14:81943260-81943261_- | 3'UTR | 2556 | 0.02955648 |
| CUL4A | 0.63004298 | hypo | chr13:113917997-113917998_+ | 3'UTR | 2504 | 0.03886832 |
| SNRNP25 | 0.63043291 | hypo | chr16:107312-107313_+ | 3'UTR | 729 | 0.04738253 |
| SYNCRIP | 0.63058738 | hypo | chr6:86322453-86322454_- | 3'UTR | 1812 | 0.04293967 |
| TRIAP1 | 0.63075116 | hypo | chr12:120882451-120882452_- | 3'UTR | 465 | 0.00879807 |
| RND3 | 0.6309061 | hypo | chr2:151326528-151326529_- | CDS | 984 | 0.00034241 |
| STARD13 | 0.63111595 | hypo | chr13:33703696-33703697_- | CDS | 1242 | 0.01621981 |
| NUDT19 | 0.63124552 | hypo | chr19:33202817-33202818_+ | CDS | 1082 | 0.00102998 |
| ENG | 0.63127466 | hypo | chr9:130577713-130577714_- | 3'UTR | 2641 | 0.00384161 |
| MRPL41 | 0.6312783 | hypo | chr9:140446867-140446868_+ | CDS | 464 | 0.04175878 |
| THRAP3 | 0.63138288 | hypo | chr1:36752719-36752720_+ | CDS | 1112 | 0.03377421 |
| DAXX | 0.63168957 | hypo | chr6:33287560-33287561_- | CDS | 1673 | 0.00427358 |
| HILPDA | 0.63172374 | hypo | chr7:128097591-128097592_+ | 3'UTR | 417 | 0.00165915 |
| MYC | 0.63209268 | hypo | chr8:128752929-128752930_+ | CDS | 2250 | 0.01350251 |
| MAT2A | 0.63228615 | hypo | chr2:85769724-85769725_+ | CDS | 1115 | 0.01784383 |
| TNFRSF1A | 0.6323361 | hypo | chr12:6437997-6437998_- | 3'UTR | 2150 | 0.01261912 |
| CENPX | 0.63243574 | hypo | chr17:79976876-79976877_- | 3'UTR | 497 | 0.00718353 |
| EDC3 | 0.63254575 | hypo | chr15:74925129-74925130_- | CDS | 1541 | 0.00832313 |
| CLEC16A | 0.63262847 | hypo | chr16:11274595-11274596_+ | 3'UTR | 5364 | 0.03174319 |
| MARCH2 | 0.63324271 | hypo | chr19:8503564-8503565_+ | 3'UTR | 1045 | 0.00333267 |
| CCM2 | 0.63339845 | hypo | chr7:45115832-45115833_+ | 3'UTR | 1645 | 0.03826756 |
| SEM1 | 0.63345395 | hypo | chr7:96339083-96339084_- | 5'UTR | 119 | 0.00947465 |
| PRRC2C | 0.63358746 | hypo | chr1:171560974-171560975_+ | CDS | 8708 | 0.00508605 |
| THUMPD1 | 0.63386824 | hypo | chr16:20745577-20745578_- | 3'UTR | 3910 | 0.00564316 |
| PTPN12 | 0.63392282 | hypo | chr7:77256143-77256144_+ | CDS | 1399 | 0.03730754 |
| PIGV | 0.6343403 | hypo | chr1:27121554-27121555_+ | CDS | 1392 | 0.01200812 |
| PPIL2 | 0.63435509 | hypo | chr22:22052044-22052045_+ | 3'UTR | 2552 | 0.00901675 |
| TGOLN2 | 0.63445934 | hypo | chr2:85554450-85554451_- | CDS | 737 | 0.0316784 |
| KAZALD1 | 0.63459091 | hypo | chr10:102827631-102827632_+ | 3'UTR | 2545 | 0.00301108 |
| YWHAH | 0.63478706 | hypo | chr22:32352720-32352721_+ | CDS | 923 | 0.0056585 |
| E4F1 | 0.63509815 | hypo | chr16:2285605-2285606_+ | 3'UTR | 2513 | 0.00558335 |
| BTBD6 | 0.63575675 | hypo | chr14:105716362-105716363_+ | CDS | 919 | 0.0462068 |
| SRSF4 | 0.63593338 | hypo | chr1:29475158-29475159_- | CDS | 1395 | 0.00646024 |
| ZNF324 | 0.63599216 | hypo | chr19:58984098-58984099_+ | 3'UTR | 2384 | 0.00207255 |
| OXLD1 | 0.63605492 | hypo | chr17:79632360-79632361_- | CDS | 373 | 0.04354887 |
| JUN | 0.63605627 | hypo | chr1:59247754-59247755_- | CDS | 2030 | 0.04169748 |
| YDJC | 0.63607792 | hypo | chr22:21982586-21982587_- | 3'UTR | 1128 | 0.02390068 |
| ADAMTS1 | 0.63611152 | hypo | chr21:28210513-28210514_- | CDS | 2742 | 0.01388328 |
| NCL | 0.63634114 | hypo | chr2:232326432-232326433_- | CDS | 571 | 0.00075848 |
| SPTBN1 | 0.63694992 | hypo | chr2:54876855-54876856_+ | CDS | 5555 | 0.03327773 |
| KHDRBS1 | 0.63696064 | hypo | chr1:32509279-32509280_+ | 3'UTR | 2514 | 0.00142484 |
| ITGA5 | 0.63748124 | hypo | chr12:54789599-54789600_- | 3'UTR | 3694 | 0.01292104 |
| COX6C | 0.63749331 | hypo | chr8:100904260-100904261_- | 5'UTR | 395 | 0.0092416 |
| LPCAT1 | 0.63781207 | hypo | chr5:1462455-1462456_- | 3'UTR | 3030 | 0.00488206 |
| S100A10 | 0.63811197 | hypo | chr1:151955530-151955531_- | 3'UTR | 909 | 0.000851 |
| YLPM1 | 0.63828513 | hypo | chr14:75248259-75248260_+ | CDS | 1637 | 0.02815558 |
| CLIP1 | 0.63830436 | hypo | chr12:122757460-122757461_- | CDS | 4432 | 0.00490306 |
| GOSR1 | 0.63840702 | hypo | chr17:28849400-28849401_+ | 3'UTR | 783 | 0.00127081 |
| PRRC2C | 0.63883261 | hypo | chr1:171510018-171510019_+ | CDS | 3673 | 0.04479474 |
| MTCH1 | 0.63888885 | hypo | chr6:36936613-36936614_- | 3'UTR | 1612 | 0.0437504 |
| TAF7 | 0.63926286 | hypo | chr5:140698193-140698194_- | 3'UTR | 2157 | 0.03098527 |
| MFAP1 | 0.63964346 | hypo | chr15:44097260-44097261_- | 3'UTR | 1534 | 0.00892931 |
| CAPN2 | 0.63968821 | hypo | chr1:223963314-223963315_+ | 3'UTR | 3082 | 0.01806568 |
| PTPMT1 | 0.64005826 | hypo | chr11:47593175-47593176_+ | CDS | 793 | 0.02110972 |
| DHX36 | 0.64013334 | hypo | chr3:153993924-153993925_- | 3'UTR | 3132 | 0.01262351 |
| UXS1 | 0.640594 | hypo | chr2:106710590-106710591_- | CDS | 1266 | 0.01143631 |
| CLTB | 0.64061733 | hypo | chr5:175824696-175824697_- | CDS | 580 | 0.0364607 |
| TRIAP1 | 0.64074945 | hypo | chr12:120882598-120882599_- | 3'UTR | 318 | 0.00019352 |
| DAP3 | 0.64080355 | hypo | chr1:155708152-155708153_+ | 3'UTR | 1404 | 0.01519429 |
| POLR3E | 0.64083657 | hypo | chr16:22343481-22343482_+ | CDS | 2246 | 0.00609709 |
| BTG1 | 0.6409751 | hypo | chr12:92537940-92537941_- | CDS | 792 | 0.01958422 |
| NSL1 | 0.64098316 | hypo | chr1:212911837-212911838_- | CDS | 771 | 0.00311137 |
| OGFR | 0.64114568 | hypo | chr20:61439626-61439627_+ | CDS | 328 | 0.01509216 |
| TUBG1 | 0.64133816 | hypo | chr17:40767069-40767070_+ | 3'UTR | 1764 | 0.04700048 |
| ACTR8 | 0.64141943 | hypo | chr3:53902711-53902712_- | 3'UTR | 1959 | 0.01313618 |
| KMT2A | 0.6417793 | hypo | chr11:118373391-118373392_+ | CDS | 6807 | 0.03400081 |
| RPS6 | 0.64187944 | hypo | chr9:19376362-19376363_- | CDS | 719 | 0.0493115 |
| MLXIP | 0.64272508 | hypo | chr12:122627460-122627461_+ | 3'UTR | 3987 | 0.00918085 |
| GSPT1 | 0.64281141 | hypo | chr16:11966733-11966734_- | 3'UTR | 2403 | 0.03654238 |
| TSPO | 0.64298324 | hypo | chr22:43558956-43558957_+ | CDS | 544 | 0.0245054 |
| CKAP4 | 0.64322315 | hypo | chr12:106633906-106633907_- | CDS | 787 | 0.03871985 |
| GMPPB | 0.64327884 | hypo | chr3:49759054-49759055_- | 3'UTR | 1541 | 0.0076811 |
| FJX1 | 0.64330774 | hypo | chr11:35642049-35642050_+ | 3'UTR | 2315 | 0.01071337 |
| ZC3H13 | 0.64331379 | hypo | chr13:46549437-46549438_- | CDS | 2537 | 0.00785798 |
| ATG5 | 0.64334658 | hypo | chr6:106634191-106634192_- | 3'UTR | 1403 | 0.0043562 |
| ADSL | 0.64335374 | hypo | chr22:40746031-40746032_+ | CDS | 408 | 0.00449369 |
| LRRFIP2 | 0.6433728 | hypo | chr3:37094897-37094898_- | 3'UTR | 2708 | 0.00780912 |
| CCDC86 | 0.64343488 | hypo | chr11:60617975-60617976_+ | 3'UTR | 1429 | 0.00482921 |
| PIK3R1 | 0.64350283 | hypo | chr5:67593905-67593906_+ | 3'UTR | 3231 | 0.03740385 |
| PAFAH1B2 | 0.64355623 | hypo | chr11:117038316-117038317_+ | CDS | 733 | 0.00147229 |
| RMRP | 0.64366937 | hypo | chr9:35657896-35657897_- | 118 | 118 | 0.02756784 |
| ZMAT2 | 0.64375743 | hypo | chr5:140080434-140080435_+ | CDS | 150 | 0.0423505 |
| WDR26 | 0.64401605 | hypo | chr1:224577324-224577325_- | 3'UTR | 2391 | 0.00300889 |
| COG8 | 0.64410746 | hypo | chr16:69368680-69368681_- | CDS | 1226 | 0.00372271 |
| FNDC3A | 0.64418335 | hypo | chr13:49781320-49781321_+ | CDS | 3691 | 0.01514453 |
| MYC | 0.64446536 | hypo | chr8:128752866-128752867_+ | CDS | 2187 | 0.01055006 |
| CLPTM1L | 0.64448017 | hypo | chr5:1318402-1318403_- | 3'UTR | 1955 | 0.04806499 |
| HYAL2 | 0.64460658 | hypo | chr3:50355478-50355479_- | 3'UTR | 1746 | 0.01497786 |
| YKT6 | 0.64478231 | hypo | chr7:44250696-44250697_+ | CDS | 691 | 0.03175601 |
| ZFYVE9 | 0.64485052 | hypo | chr1:52704281-52704282_+ | CDS | 1644 | 0.03607775 |
| OGFOD1 | 0.64508851 | hypo | chr16:56510053-56510054_+ | CDS | 1666 | 0.01475984 |
| EEF2 | 0.64541354 | hypo | chr19:3976160-3976161_- | 3'UTR | 3050 | 0.04563102 |
| AFF4 | 0.64548833 | hypo | chr5:132232385-132232386_- | CDS | 2315 | 0.02451811 |
| CKAP4 | 0.64554317 | hypo | chr12:106632937-106632938_- | CDS | 1756 | 0.04563092 |
| TSHZ1 | 0.6457112 | hypo | chr18:73000391-73000392_+ | CDS | 3478 | 0.00029249 |
| LRWD1 | 0.64574091 | hypo | chr7:102113522-102113523_+ | 3'UTR | 2067 | 0.01601674 |
| DSE | 0.64597492 | hypo | chr6:116757927-116757928_+ | CDS | 2547 | 0.04350118 |
| PSMB5 | 0.64611337 | hypo | chr14:23495240-23495241_- | 3'UTR | 1112 | 0.01760198 |
| HTRA1 | 0.64617515 | hypo | chr10:124273807-124273808_+ | CDS | 1503 | 0.02897563 |
| GLG1 | 0.64647552 | hypo | chr16:74486331-74486332_- | 3'UTR | 4322 | 0.0055355 |
| EIF5B | 0.64686077 | hypo | chr2:99953957-99953958_+ | 5'UTR | 124 | 0.01046041 |
| RPS4X | 0.64767407 | hypo | chrX:71492503-71492504_- | 3'UTR | 904 | 0.01213936 |
| CHMP7 | 0.64778181 | hypo | chr8:23119216-23119217_+ | 3'UTR | 3114 | 0.00403377 |
| FAM32A | 0.64778224 | hypo | chr19:16302478-16302479_+ | 3'UTR | 1093 | 0.03605419 |
| ABCF1 | 0.64823626 | hypo | chr6:30545212-30545213_+ | CDS | 195 | 0.04727457 |
| SMG1 | 0.64846886 | hypo | chr16:18820850-18820851_- | 3'UTR | 11388 | 0.03450702 |
| AHNAK2 | 0.64848187 | hypo | chr14:105420641-105420642_- | CDS | 1265 | 0.00395695 |
| RPL19 | 0.6488689 | hypo | chr17:37358653-37358654_+ | CDS | 258 | 0.02444271 |
| ITPR2 | 0.64897708 | hypo | chr12:26492366-26492367_- | CDS | 8481 | 0.00035099 |
| EIF4G1 | 0.64944064 | hypo | chr3:184040966-184040967_+ | CDS | 2260 | 0.00177383 |
| ANKRD11 | 0.65014379 | hypo | chr16:89349803-89349804_- | CDS | 3603 | 0.04376127 |
| EIF5B | 0.65017423 | hypo | chr2:99978144-99978145_+ | CDS | 964 | 0.02750301 |
| NDUFAF8 | 0.65026713 | hypo | chr17:79214936-79214937_+ | 3'UTR | 398 | 0.03561282 |
| PANX1 | 0.65054404 | hypo | chr11:93914375-93914376_+ | 3'UTR | 2006 | 0.04635013 |
| NCBP2 | 0.65064694 | hypo | chr3:196663733-196663734_- | 3'UTR | 708 | 0.04515913 |
| SPOP | 0.65090802 | hypo | chr17:47677592-47677593_- | 3'UTR | 1568 | 0.01364879 |
| PSEN1 | 0.65102469 | hypo | chr14:73686195-73686196_+ | 3'UTR | 1814 | 0.02723719 |
| ATMIN | 0.65106911 | hypo | chr16:81077649-81077650_+ | CDS | 1564 | 0.04834215 |
| TCF19 | 0.65107654 | hypo | chr6:31130999-31131000_+ | 3'UTR | 2067 | 0.03730994 |
| UTP3 | 0.65150662 | hypo | chr4:71555372-71555373_+ | CDS | 1177 | 0.04277966 |
| ANKRD11 | 0.65166491 | hypo | chr16:89353318-89353319_- | 1777 | 1777 | 0.02028375 |
| CHMP1B | 0.65187238 | hypo | chr18:11852201-11852202_+ | 3'UTR | 813 | 0.02298604 |
| RRAGA | 0.65189285 | hypo | chr9:19049856-19049857_+ | CDS | 485 | 0.01756563 |
| PAF1 | 0.65199099 | hypo | chr19:39876602-39876603_- | 3'UTR | 1865 | 0.02773287 |
| SIAH2 | 0.65217584 | hypo | chr3:150460197-150460198_- | CDS | 1003 | 0.00133975 |
| NETO2 | 0.65256638 | hypo | chr16:47117450-47117451_- | CDS | 1671 | 0.01573239 |
| CHCHD3 | 0.65289272 | hypo | chr7:132470183-132470184_- | 3'UTR | 1118 | 0.01719953 |
| TBC1D20 | 0.6529091 | hypo | chr20:418682-418683_- | 3'UTR | 1906 | 0.0325474 |
| ETFDH | 0.65335351 | hypo | chr4:159629540-159629541_+ | CDS | 2071 | 0.00576467 |
| TRAF2 | 0.65340443 | hypo | chr9:139821032-139821033_+ | 3'UTR | 2242 | 0.02597547 |
| MRPS33 | 0.65373963 | hypo | chr7:140706055-140706056_- | 3'UTR | 592 | 0.02473391 |
| TMEM60 | 0.65381959 | hypo | chr7:77423154-77423155_- | 3'UTR | 766 | 0.03038794 |
| RRAGA | 0.65413891 | hypo | chr9:19049794-19049795_+ | CDS | 423 | 0.03670725 |
| SCO1 | 0.65459797 | hypo | chr17:10584354-10584355_- | 3'UTR | 1047 | 0.02504269 |
| NR2F6 | 0.65461352 | hypo | chr19:17342905-17342906_- | 3'UTR | 1591 | 0.02495307 |
| HMGB2 | 0.65466459 | hypo | chr4:174254094-174254095_- | CDS | 467 | 0.04608646 |
| CHD2 | 0.65471759 | hypo | chr15:93570773-93570774_+ | 3'UTR | 8900 | 0.00818388 |
| NECAP2 | 0.65516692 | hypo | chr1:16786439-16786440_+ | 3'UTR | 1860 | 0.0141379 |
| NAB2 | 0.65529251 | hypo | chr12:57488847-57488848_+ | 3'UTR | 2299 | 0.00683023 |
| GRPEL1 | 0.65534319 | hypo | chr4:7062455-7062456_- | 3'UTR | 964 | 0.03180808 |
| RAB13 | 0.65556738 | hypo | chr1:153954528-153954529_- | 3'UTR | 798 | 0.03822069 |
| NUCKS1 | 0.65559607 | hypo | chr1:205689662-205689663_- | CDS | 618 | 0.00778061 |
| SYTL2 | 0.65561304 | hypo | chr11:85406161-85406162_- | 3'UTR | 7372 | 0.00696981 |
| GALNT2 | 0.65573941 | hypo | chr1:230415560-230415561_+ | 3'UTR | 2158 | 0.02612995 |
| EIF3L | 0.65616918 | hypo | chr22:38273789-38273790_+ | CDS | 1264 | 0.03760477 |
| NCOA1 | 0.65676343 | hypo | chr2:24930476-24930477_+ | CDS | 2881 | 0.01979716 |
| SMAD2 | 0.65710682 | hypo | chr18:45422964-45422965_- | CDS | 562 | 0.00528462 |
| TDP1 | 0.6571379 | hypo | chr14:90429801-90429802_+ | CDS | 419 | 0.02574879 |
| DCUN1D3 | 0.65716517 | hypo | chr16:20871475-20871476_- | CDS | 906 | 0.00299238 |
| SPIRE1 | 0.65794718 | hypo | chr18:12449126-12449127_- | 3'UTR | 2827 | 0.02502595 |
| UBXN1 | 0.65798378 | hypo | chr11:62445502-62445503_- | CDS | 571 | 0.03137052 |
| POP5 | 0.65813727 | hypo | chr12:121016996-121016997_- | 3'UTR | 647 | 0.01044326 |
| AFG3L2 | 0.65817878 | hypo | chr18:12329618-12329619_- | CDS | 2532 | 0.01984577 |
| EBLN3P | 0.65817883 | hypo | chr9:37086809-37086810_+ | 786 | 786 | 0.02523671 |
| ATMIN | 0.65818703 | hypo | chr16:81078501-81078502_+ | CDS | 2416 | 0.01664711 |
| MCL1 | 0.65832073 | hypo | chr1:150550827-150550828_- | CDS | 1035 | 0.04494716 |
| ATP1A1 | 0.65863902 | hypo | chr1:116941605-116941606_+ | CDS | 2686 | 0.02792597 |
| CUEDC1 | 0.65873715 | hypo | chr17:55940549-55940550_- | 3'UTR | 1674 | 0.01016869 |
| ZBTB4 | 0.65885073 | hypo | chr17:7363216-7363217_- | 3'UTR | 5409 | 0.02369213 |
| TIMM8A | 0.65892419 | hypo | chrX:100601634-100601635_- | CDS | 223 | 0.04647463 |
| MED6 | 0.65914333 | hypo | chr14:71051521-71051522_- | 3'UTR | 801 | 0.02203907 |
| SUMO3 | 0.65954681 | hypo | chr21:46226766-46226767_- | 3'UTR | 571 | 0.0211907 |
| DYNC1H1 | 0.65958332 | hypo | chr14:102452858-102452859_+ | CDS | 2460 | 0.01674548 |
| CNOT2 | 0.6597732 | hypo | chr12:70748602-70748603_+ | 3'UTR | 3112 | 0.03622144 |
| EMP2 | 0.65993482 | hypo | chr16:10625780-10625781_- | 3'UTR | 1712 | 0.0227872 |
| MRPS7 | 0.66005778 | hypo | chr17:73261869-73261870_+ | CDS | 827 | 0.03800226 |
| DDX5 | 0.66006779 | hypo | chr17:62496401-62496402_- | CDS | 1885 | 0.02356812 |
| FASN | 0.66009633 | hypo | chr17:80054229-80054230_- | CDS | 208 | 0.03942578 |
| CDS2 | 0.66055774 | hypo | chr20:5170960-5170961_+ | 3'UTR | 1750 | 0.01019397 |
| OSBP | 0.6606513 | hypo | chr11:59344082-59344083_- | CDS | 2870 | 0.01464528 |
| CCDC47 | 0.66073508 | hypo | chr17:61824142-61824143_- | 3'UTR | 1885 | 0.01755778 |
| TOMM5 | 0.66075871 | hypo | chr9:37588786-37588787_- | 3'UTR | 529 | 0.01693777 |
| DCAF5 | 0.66088646 | hypo | chr14:69522098-69522099_- | CDS | 1522 | 0.04263635 |
| GLG1 | 0.66099502 | hypo | chr16:74486805-74486806_- | 3'UTR | 3848 | 0.00460883 |
| VSIR | 0.6610193 | hypo | chr10:73510125-73510126_- | 3'UTR | 1900 | 0.03390988 |
| PHACTR2 | 0.66102272 | hypo | chr6:144086682-144086683_+ | CDS | 1178 | 0.01982153 |
| BAG3 | 0.66108589 | hypo | chr10:121436663-121436664_+ | CDS | 1903 | 0.02966094 |
| TCEAL8 | 0.66122819 | hypo | chrX:102508716-102508717_- | CDS | 344 | 0.00477927 |
| SSRP1 | 0.66146844 | hypo | chr11:57095827-57095828_- | CDS | 1820 | 0.01314597 |
| MAT2A | 0.66150781 | hypo | chr2:85771693-85771694_+ | 3'UTR | 2296 | 0.01567155 |
| CREG1 | 0.66254457 | hypo | chr1:167510632-167510633_- | 3'UTR | 1642 | 0.0071761 |
| NRP2 | 0.66290227 | hypo | chr2:206547866-206547867_+ | 5'UTR | 643 | 0.00035822 |
| SUSD1 | 0.66291108 | hypo | chr9:114803415-114803416_- | 3'UTR | 2661 | 0.01792201 |
| CTNNB1 | 0.66292206 | hypo | chr3:41280694-41280695_+ | CDS | 2475 | 0.01949041 |
| SDF2 | 0.6629916 | hypo | chr17:26976088-26976089_- | CDS | 849 | 0.01325743 |
| MRPL50 | 0.66309647 | hypo | chr9:104152730-104152731_- | 3'UTR | 538 | 0.02166136 |
| TFRC | 0.66337793 | hypo | chr3:195778785-195778786_- | 3'UTR | 2451 | 0.01332022 |
| RHOB | 0.66340313 | hypo | chr2:20648449-20648450_+ | 3'UTR | 1618 | 0.02453109 |
| C12orf43 | 0.66346474 | hypo | chr12:121441159-121441160_- | 3'UTR | 1612 | 0.01109953 |
| PIGO | 0.66356934 | hypo | chr9:35088955-35088956_- | 3'UTR | 3798 | 0.01409711 |
| SEC23A | 0.66372584 | hypo | chr14:39502123-39502124_- | 3'UTR | 3134 | 0.01541024 |
| SEC61A1 | 0.66387974 | hypo | chr3:127789484-127789485_+ | 3'UTR | 2594 | 0.03543404 |
| ABL1 | 0.66389766 | hypo | chr9:133761705-133761706_+ | 3'UTR | 4524 | 0.01152664 |
| MED11 | 0.66439563 | hypo | chr17:4636604-4636605_+ | 3'UTR | 538 | 0.03420028 |
| ATP1B1 | 0.6644529 | hypo | chr1:169100686-169100687_+ | CDS | 926 | 0.03774072 |
| COMMD5 | 0.66471791 | hypo | chr8:146076010-146076011_- | 3'UTR | 844 | 0.01426806 |
| ZBTB38 | 0.66491883 | hypo | chr3:141164198-141164199_+ | CDS | 3950 | 0.0075529 |
| RPP38 | 0.66494298 | hypo | chr10:15145941-15145942_+ | CDS | 868 | 0.01896246 |
| TTPAL | 0.66518977 | hypo | chr20:43118372-43118373_+ | 3'UTR | 1340 | 0.00291749 |
| ELOA | 0.66532329 | hypo | chr1:24078059-24078060_+ | CDS | 1102 | 0.01472207 |
| GON4L | 0.66539286 | hypo | chr1:155735322-155735323_- | CDS | 4115 | 0.02372211 |
| ADGRE5 | 0.66547701 | hypo | chr19:14519243-14519244_+ | 3'UTR | 3198 | 0.04598547 |
| GNA12 | 0.66556272 | hypo | chr7:2770857-2770858_- | CDS | 1266 | 0.00834483 |
| PELO | 0.6656125 | hypo | chr5:52097577-52097578_+ | CDS | 2046 | 0.02061778 |
| RSF1 | 0.66567215 | hypo | chr11:77378168-77378169_- | CDS | 4238 | 0.04171027 |
| CLPB | 0.66569383 | hypo | chr11:72004564-72004565_- | CDS | 2002 | 0.03586876 |
| LPAR6 | 0.66572689 | hypo | chr13:48985454-48985455_- | 3'UTR | 2198 | 0.03120741 |
| DHRS7B | 0.66573316 | hypo | chr17:21094664-21094665_+ | 3'UTR | 1220 | 0.01530432 |
| ERP29 | 0.66585719 | hypo | chr12:112460361-112460362_+ | CDS | 809 | 0.03049443 |
| AP2A2 | 0.66603751 | hypo | chr11:1010879-1010880_+ | 3'UTR | 3290 | 0.0192925 |
| SH2D4A | 0.6661366 | hypo | chr8:19177159-19177160_+ | CDS | 409 | 0.0061512 |
| PPP2R5E | 0.66631271 | hypo | chr14:63842504-63842505_- | 3'UTR | 2231 | 0.01078656 |
| ASB8 | 0.66663427 | hypo | chr12:48543261-48543262_- | CDS | 923 | 0.0396006 |
