# Supplementary material for: Specific RNA m6A modification sites in bone marrow mesenchymal stem cells from the jawbone marrow of type 2 diabetes patients with dental implant failure
Source: Int J Oral Sci. 2023 Jan 12;15:6. doi: 10.1038/s41368-022-00202-3 (PMC9834262; doi:10.1038/s41368-022-00202-3)
Supplement: Supplementary file 3 — Supplementary Table S3 [file 41368_2022_202_MOESM3_ESM.docx]

**Table S3. List of the hypermethylated genes in DM-BMSCs (based on “m6A site abundance”).**

| **Gene symbol** | **Fold change** | **Regulation in T2DM** | **m6A site Locus** | **m6A location** | **m6A transcript location** | **p‐value** |
| --- | --- | --- | --- | --- | --- | --- |
| LRIG3 | 3.57000284 | hyper | chr12:59266448-59266449_- | CDS | 3567 | 0.04042084 |
| BICD2 | 2.43134553 | hyper | chr9:95481643-95481644_- | CDS | 1339 | 0.00321641 |
| KLF12 | 2.38203889 | hyper | chr13:74420373-74420374_- | CDS | 481 | 0.0414019 |
| GCC1 | 2.36994885 | hyper | chr7:127224772-127224773_- | CDS | 881 | 0.00045115 |
| ZFYVE19 | 2.36408441 | hyper | chr15:41099880-41099881_+ | CDS | 607 | 0.00042119 |
| RPUSD4 | 2.31467914 | hyper | chr11:126073184-126073185_- | 3'UTR | 1273 | 0.00047447 |
| INF2 | 2.25598998 | hyper | chr14:105181031-105181032_+ | CDS | 3675 | 0.00094368 |
| SPAG5 | 2.23819644 | hyper | chr17:26919722-26919723_- | CDS | 630 | 0.02671955 |
| PALM | 2.2301235 | hyper | chr19:747431-747432_+ | 3'UTR | 1975 | 0.01573864 |
| HIVEP2 | 2.19072394 | hyper | chr6:143094997-143094998_- | CDS | 1595 | 0.02403242 |
| ENC1 | 2.12003962 | hyper | chr5:73931700-73931701_- | CDS | 1740 | 0.00712663 |
| ZBTB37 | 2.11046782 | hyper | chr1:173855263-173855264_+ | 3'UTR | 1844 | 0.0110097 |
| MGAT1 | 2.04541565 | hyper | chr5:180219603-180219604_- | CDS | 862 | 0.015066 |
| FOXK2 | 1.98070618 | hyper | chr17:80561336-80561337_+ | 3'UTR | 4115 | 0.00398341 |
| TIPARP | 1.90047889 | hyper | chr3:156396123-156396124_+ | CDS | 709 | 0.04426516 |
| HSPA1L | 1.89782001 | hyper | chr6:31779069-31779070_- | CDS | 862 | 0.00591342 |
| AJUBA | 1.82630264 | hyper | chr14:23451737-23451738_- | 5'UTR | 113 | 0.01609623 |
| FBXL4 | 1.81241789 | hyper | chr6:99374560-99374561_- | CDS | 694 | 0.03777674 |
| GLYR1 | 1.77548626 | hyper | chr16:4855048-4855049_- | 3'UTR | 1886 | 0.00030108 |
| BORCS8 | 1.77364741 | hyper | chr19:19287807-19287808_- | 3'UTR | 1337 | 0.01189891 |
| ZNF394 | 1.70231861 | hyper | chr7:99091974-99091975_- | CDS | 1050 | 0.00043548 |
| UBP1 | 1.69522896 | hyper | chr3:33431389-33431390_- | 3'UTR | 2204 | 0.00654778 |
| PRAG1 | 1.68132762 | hyper | chr8:8235178-8235179_- | CDS | 826 | 0.00712751 |
| FAM120B | 1.67237324 | hyper | chr6:170627013-170627014_+ | CDS | 677 | 0.00320248 |
| TAF5L | 1.67188359 | hyper | chr1:229729855-229729856_- | 3'UTR | 2123 | 0.0051993 |
| ZNF623 | 1.64564638 | hyper | chr8:144733324-144733325_+ | CDS | 1371 | 0.01806813 |
| SPEN | 1.64090294 | hyper | chr1:16266165-16266166_+ | 3'UTR | 11442 | 0.03422931 |
| ENDOD1 | 1.63658196 | hyper | chr11:94862306-94862307_+ | CDS | 1184 | 0.02567606 |
| SOGA1 | 1.62701795 | hyper | chr20:35414202-35414203_- | 3'UTR | 6010 | 0.00533334 |
| DOLPP1 | 1.61935145 | hyper | chr9:131851435-131851436_+ | 3'UTR | 894 | 0.04312937 |
| TRIAP1 | 1.61913243 | hyper | chr12:120884083-120884084_- | CDS | 103 | 0.01754829 |
| SKIDA1 | 1.59972679 | hyper | chr10:21804404-21804405_- | CDS | 4599 | 0.03122367 |
| ECHDC1 | 1.58792306 | hyper | chr6:127664550-127664551_- | 5'UTR | 203 | 0.01110182 |
| PDE4B | 1.57910563 | hyper | chr1:66838243-66838244_+ | CDS | 2517 | 0.03092313 |
| LMTK3 | 1.57335327 | hyper | chr19:49001714-49001715_- | CDS | 2697 | 0.02463836 |
| TMTC2 | 1.57101382 | hyper | chr12:83289942-83289943_+ | CDS | 1432 | 0.00209243 |
| OXLD1 | 1.57048357 | hyper | chr17:79633110-79633111_- | 3'UTR | 556 | 0.01682009 |
| TEX2 | 1.56759615 | hyper | chr17:62290388-62290389_- | CDS | 1369 | 0.04160269 |
| ZNF749 | 1.56582852 | hyper | chr19:57955091-57955092_+ | CDS | 829 | 0.01047298 |
| SMIM13 | 1.53828261 | hyper | chr6:11135867-11135868_+ | 3'UTR | 1589 | 0.02496182 |
| HOXB9 | 1.52468526 | hyper | chr17:46703161-46703162_- | CDS | 673 | 0.04951251 |
| TRERF1 | 1.52434912 | hyper | chr6:42196060-42196061_- | 3'UTR | 4193 | 0.02937745 |
| HOMER1 | 1.51492866 | hyper | chr5:78808715-78808716_- | 5'UTR | 943 | 0.00967291 |
| ZNF827 | 1.50366812 | hyper | chr4:146682324-146682325_- | 3'UTR | 4250 | 0.02120432 |
