# Supplementary material for: Specific RNA m6A modification sites in bone marrow mesenchymal stem cells from the jawbone marrow of type 2 diabetes patients with dental implant failure
Source: Int J Oral Sci. 2023 Jan 12;15:6. doi: 10.1038/s41368-022-00202-3 (PMC9834262; doi:10.1038/s41368-022-00202-3)
Supplement: Supplementary file 4 — Supplementary Table S4 [file 41368_2022_202_MOESM4_ESM.docx]

**Table S4. List of the hypomethylated genes in DM-BMSCs (based on “m6A site abundance”).**

| **Gene symbol** | **Fold change** | **Regulation in T2DM** | **m6A site Locus** | **m6A location** | **m6A transcript location** | **p‐value** |
| --- | --- | --- | --- | --- | --- | --- |
| LURAP1L | 0.14609692 | hypo | chr9:12821761-12821762_+ | 3'UTR | 1385 | 0.0220062 |
| TRIB3 | 0.18409242 | hypo | chr20:377308-377309_+ | CDS | 1592 | 0.03629275 |
| RCAN1 | 0.21916652 | hypo | chr21:35890113-35890114_- | 3'UTR | 1128 | 0.00968516 |
| PRPSAP1 | 0.24111758 | hypo | chr17:74307525-74307526_- | 3'UTR | 1700 | 0.03326727 |
| RRAGC | 0.24608375 | hypo | chr1:39304703-39304704_- | 3'UTR | 1897 | 0.02946882 |
| TAF3 | 0.24980615 | hypo | chr10:8006865-8006866_+ | CDS | 1598 | 0.00623559 |
| ZNF611 | 0.25755714 | hypo | chr19:53208172-53208173_- | 3'UTR | 2309 | 0.00361353 |
| RND3 | 0.25792018 | hypo | chr2:151326476-151326477_- | 3'UTR | 1036 | 0.01557677 |
| POLL | 0.26226213 | hypo | chr10:103339350-103339351_- | CDS | 2068 | 0.00779575 |
| MYNN | 0.27020712 | hypo | chr3:169496792-169496793_+ | CDS | 534 | 0.00413092 |
| GTPBP4 | 0.2793826 | hypo | chr10:1063269-1063270_+ | 3'UTR | 2078 | 0.0277608 |
| ZFP62 | 0.28361787 | hypo | chr5:180277703-180277704_- | CDS | 857 | 0.01082962 |
| PSD3 | 0.28603499 | hypo | chr8:18725537-18725538_- | CDS | 1382 | 0.04048746 |
| RPP25 | 0.29269512 | hypo | chr15:75248079-75248080_- | 3'UTR | 1695 | 0.03102241 |
| CDYL | 0.29933398 | hypo | chr6:4892247-4892248_+ | CDS | 663 | 0.00125593 |
| CDK12 | 0.30006635 | hypo | chr17:37687213-37687214_+ | CDS | 4678 | 0.03580269 |
| CA12 | 0.30596072 | hypo | chr15:63616587-63616588_- | 3'UTR | 3086 | 0.04449593 |
| DDIT3 | 0.30815819 | hypo | chr12:57910590-57910591_- | CDS | 771 | 0.03393714 |
| STC2 | 0.30934927 | hypo | chr5:172744696-172744697_- | 3'UTR | 2371 | 0.03096 |
| SIRT7 | 0.31051682 | hypo | chr17:79870272-79870273_- | 3'UTR | 1272 | 0.01795684 |
| NIPBL | 0.31566318 | hypo | chr5:36985840-36985841_+ | CDS | 3057 | 0.04115437 |
| MON2 | 0.31989062 | hypo | chr12:62986604-62986605_+ | 3'UTR | 5620 | 0.02340573 |
| ADNP | 0.32309126 | hypo | chr20:49507916-49507917_- | 3'UTR | 3726 | 0.00941851 |
| RND3 | 0.32446865 | hypo | chr2:151326634-151326635_- | CDS | 878 | 0.02384219 |
| RND3 | 0.32546484 | hypo | chr2:151326672-151326673_- | CDS | 840 | 0.02160277 |
| NFE2L2 | 0.32707769 | hypo | chr2:178096059-178096060_- | CDS | 1825 | 0.02943563 |
| TENT5A | 0.32712415 | hypo | chr6:82459757-82459758_- | CDS | 1300 | 0.04346513 |
| C1R | 0.32956997 | hypo | chr12:7187962-7187963_- | CDS | 1815 | 0.03496146 |
| MINDY3 | 0.33048696 | hypo | chr10:15820956-15820957_- | 3'UTR | 1605 | 0.01911655 |
| PSMA3-AS1 | 0.33186357 | hypo | chr14:58733299-58733300_- | 1265 | 1265 | 0.00702665 |
| CDV3 | 0.3324933 | hypo | chr3:133307484-133307485_+ | 3'UTR | 1826 | 0.00162961 |
| ANKRD11 | 0.33271291 | hypo | chr16:89349803-89349804_- | CDS | 3603 | 0.03147861 |
| ZBTB5 | 0.33637312 | hypo | chr9:37438676-37438677_- | 3'UTR | 4071 | 0.00675147 |
| MAGEF1 | 0.33697472 | hypo | chr3:184429207-184429208_- | CDS | 628 | 0.02730784 |
| ARHGEF3 | 0.33812358 | hypo | chr3:56763385-56763386_- | CDS | 1698 | 0.00682778 |
| GPATCH8 | 0.3395539 | hypo | chr17:42477702-42477703_- | CDS | 1777 | 0.01320967 |
| NDUFV3 | 0.34427655 | hypo | chr21:44324304-44324305_+ | CDS | 1207 | 0.00927737 |
| SEMA3F | 0.3449615 | hypo | chr3:50225958-50225959_+ | 3'UTR | 2970 | 0.01313772 |
| LONP1 | 0.34621971 | hypo | chr19:5692111-5692112_- | CDS | 2936 | 0.01183952 |
| SPATA13 | 0.34729061 | hypo | chr13:24878789-24878790_+ | 3'UTR | 6040 | 0.04809641 |
| PARP9 | 0.34782244 | hypo | chr3:122247317-122247318_- | CDS | 2679 | 0.02991503 |
| ZFP62 | 0.35307892 | hypo | chr5:180277115-180277116_- | CDS | 1445 | 0.00705071 |
| NEAT1 | 0.35866943 | hypo | chr11:65193479-65193480_+ | 3211 | 3211 | 0.04772611 |
| RBM17 | 0.36158285 | hypo | chr10:6157509-6157510_+ | CDS | 1422 | 0.03763728 |
| ARL4C | 0.36381158 | hypo | chr2:235404712-235404713_- | CDS | 980 | 0.00602447 |
| ERC1 | 0.36395884 | hypo | chr12:1600189-1600190_+ | 3'UTR | 4415 | 0.01947522 |
| C1orf50 | 0.36631135 | hypo | chr1:43240958-43240959_+ | CDS | 560 | 0.04083797 |
| FBXL20 | 0.36691827 | hypo | chr17:37417712-37417713_- | CDS | 1564 | 0.00420891 |
| NXT1 | 0.36797225 | hypo | chr20:23335211-23335212_+ | 3'UTR | 920 | 0.00915958 |
| SNW1 | 0.36920797 | hypo | chr14:78184363-78184364_- | 3'UTR | 1704 | 0.03378731 |
| BAG5 | 0.36986112 | hypo | chr14:104026688-104026689_- | CDS | 1246 | 0.00610645 |
| CRKL | 0.37020023 | hypo | chr22:21307461-21307462_+ | 3'UTR | 4749 | 0.03313328 |
| KAT5 | 0.37053216 | hypo | chr11:65486656-65486657_+ | 3'UTR | 1911 | 0.00374781 |
| SRSF5 | 0.3728605 | hypo | chr14:70238573-70238574_+ | 3'UTR | 1398 | 0.00795089 |
| CMTR2 | 0.37340116 | hypo | chr16:71318746-71318747_- | CDS | 1482 | 0.04482588 |
| SMURF1 | 0.37425281 | hypo | chr7:98626390-98626391_- | 3'UTR | 4429 | 0.0379138 |
| IFNGR1 | 0.3749973 | hypo | chr6:137519124-137519125_- | 3'UTR | 1615 | 0.04302948 |
| WDR53 | 0.37547352 | hypo | chr3:196287972-196287973_- | CDS | 976 | 0.02514808 |
| SPPL3 | 0.37603374 | hypo | chr12:121202439-121202440_- | 3'UTR | 2001 | 0.01646218 |
| RAB9A | 0.37739414 | hypo | chrX:13727023-13727024_+ | CDS | 352 | 0.03556644 |
| FBXO42 | 0.37794624 | hypo | chr1:16577199-16577200_- | CDS | 2372 | 0.02151046 |
| NCBP3 | 0.38045661 | hypo | chr17:3715661-3715662_- | 3'UTR | 2561 | 0.02966895 |
| TBC1D15 | 0.38063187 | hypo | chr12:72317011-72317012_+ | 3'UTR | 2178 | 0.01568142 |
| PCK2 | 0.38102986 | hypo | chr14:24573198-24573199_+ | 3'UTR | 2223 | 0.04226419 |
| MPPE1 | 0.38142798 | hypo | chr18:11884451-11884452_- | CDS | 1978 | 0.00790663 |
| PRPF18 | 0.38165996 | hypo | chr10:13672363-13672364_+ | 3'UTR | 1174 | 0.01068638 |
| VIPAS39 | 0.38188147 | hypo | chr14:77893565-77893566_- | 3'UTR | 2020 | 0.00954473 |
| USP31 | 0.38458598 | hypo | chr16:23080564-23080565_- | CDS | 2860 | 0.02839472 |
| CLASP1 | 0.38490208 | hypo | chr2:122097700-122097701_- | 3'UTR | 5742 | 0.0140759 |
| PSMD7 | 0.38512203 | hypo | chr16:74334092-74334093_+ | CDS | 294 | 0.01817172 |
| C6orf48 | 0.38993358 | hypo | chr6:31807329-31807330_+ | CDS | 537 | 0.03970847 |
| PRPF38A | 0.39346564 | hypo | chr1:52883512-52883513_+ | 3'UTR | 2237 | 0.00192259 |
| SIX5 | 0.39426707 | hypo | chr19:46268955-46268956_- | CDS | 2417 | 0.02501819 |
| SNHG29 | 0.39431749 | hypo | chr17:16344798-16344799_+ | 658 | 658 | 0.02352037 |
| ZNF484 | 0.39524456 | hypo | chr9:95608668-95608669_- | CDS | 2578 | 0.03132046 |
| NFE2L1 | 0.39601633 | hypo | chr17:46136713-46136714_+ | CDS | 2680 | 0.03826161 |
| PPP1R15B | 0.39605987 | hypo | chr1:204378810-204378811_- | CDS | 2134 | 0.02038183 |
| RAB32 | 0.39695604 | hypo | chr6:146875682-146875683_+ | CDS | 799 | 0.02778867 |
| SPIRE1 | 0.3970814 | hypo | chr18:12449126-12449127_- | 3'UTR | 2827 | 0.01767701 |
| HRH1 | 0.39814121 | hypo | chr3:11301719-11301720_+ | CDS | 1058 | 0.04458905 |
| SIN3B | 0.39988276 | hypo | chr19:16989466-16989467_+ | CDS | 3451 | 0.02683919 |
| BOD1L1 | 0.40013013 | hypo | chr4:13606592-13606593_- | CDS | 2047 | 0.03745039 |
| SLC3A2 | 0.40018633 | hypo | chr11:62655652-62655653_+ | CDS | 1750 | 0.02589092 |
| ARL4C | 0.40233185 | hypo | chr2:235403872-235403873_- | 3'UTR | 1820 | 0.01114736 |
| PTTG1IP | 0.40292087 | hypo | chr21:46269800-46269801_- | 3'UTR | 2524 | 0.04026248 |
| ZNF106 | 0.40396794 | hypo | chr15:42740468-42740469_- | CDS | 3228 | 0.00215125 |
| ERP29 | 0.40499257 | hypo | chr12:112460361-112460362_+ | CDS | 809 | 0.02275754 |
| ARL6IP5 | 0.40520588 | hypo | chr3:69134196-69134197_+ | 5'UTR | 109 | 0.03015294 |
| MBP | 0.40610341 | hypo | chr18:74728302-74728303_- | 3'UTR | 1324 | 0.02924846 |
| C6orf62 | 0.40660948 | hypo | chr6:24706187-24706188_- | 3'UTR | 1373 | 0.01953924 |
| YTHDC2 | 0.40707249 | hypo | chr5:112929197-112929198_+ | 3'UTR | 4553 | 0.02076592 |
| ABHD13 | 0.40736662 | hypo | chr13:108882501-108882502_+ | CDS | 1200 | 0.01981947 |
| H2AFY2 | 0.41038203 | hypo | chr10:71871688-71871689_+ | 3'UTR | 1827 | 0.02488137 |
| B3GALNT2 | 0.41070845 | hypo | chr1:235613520-235613521_- | CDS | 1731 | 0.00414687 |
| ZNF787 | 0.41096815 | hypo | chr19:56599127-56599128_- | 3'UTR | 1543 | 0.00508496 |
| NAB1 | 0.41102305 | hypo | chr2:191555162-191555163_+ | 3'UTR | 2139 | 0.00235874 |
| TRIAP1 | 0.41121347 | hypo | chr12:120882451-120882452_- | 3'UTR | 465 | 0.00480045 |
| HSPA13 | 0.41489249 | hypo | chr21:15746266-15746267_- | CDS | 1155 | 0.04679911 |
| CORO1C | 0.41604531 | hypo | chr12:109041245-109041246_- | CDS | 1565 | 0.02689488 |
| CCNL1 | 0.41622243 | hypo | chr3:156865954-156865955_- | 3'UTR | 2321 | 0.03491191 |
| TUSC1 | 0.41640698 | hypo | chr9:25677358-25677359_- | 3'UTR | 1497 | 0.00612897 |
| SREK1 | 0.41760498 | hypo | chr5:65474598-65474599_+ | CDS | 1936 | 0.00896341 |
| ZBED5 | 0.41931208 | hypo | chr11:10875719-10875720_- | CDS | 1219 | 0.03321641 |
| RAB5A | 0.42419258 | hypo | chr3:20025541-20025542_+ | 3'UTR | 1409 | 0.02312765 |
| KBTBD2 | 0.42454281 | hypo | chr7:32908898-32908899_- | 3'UTR | 2588 | 0.03036375 |
| FBXO11 | 0.42705307 | hypo | chr2:48035024-48035025_- | 3'UTR | 3088 | 0.00548296 |
| TCF20 | 0.42717114 | hypo | chr22:42606164-42606165_- | CDS | 5283 | 0.00616235 |
| HNRNPU | 0.42953751 | hypo | chr1:245021531-245021532_- | CDS | 1492 | 0.01069482 |
| CYBC1 | 0.43042748 | hypo | chr17:80400951-80400952_- | 3'UTR | 1881 | 0.00652107 |
| NFE2L1 | 0.43087076 | hypo | chr17:46136653-46136654_+ | CDS | 2620 | 0.03763618 |
| AVL9 | 0.43124154 | hypo | chr7:32598997-32598998_+ | CDS | 1420 | 0.02255396 |
| DNAJB9 | 0.43131507 | hypo | chr7:108213350-108213351_+ | CDS | 594 | 0.00064256 |
| ADSL | 0.43172914 | hypo | chr22:40746031-40746032_+ | CDS | 408 | 0.01232004 |
| ZBTB10 | 0.43326705 | hypo | chr8:81412359-81412360_+ | CDS | 2201 | 0.01652334 |
| IER3 | 0.43361783 | hypo | chr6:30711296-30711297_- | 3'UTR | 918 | 0.03671043 |
| POLR2D | 0.43847143 | hypo | chr2:128604551-128604552_- | 3'UTR | 1612 | 0.03805459 |
| F2R | 0.43869419 | hypo | chr5:76030388-76030389_+ | 3'UTR | 2603 | 0.01571421 |
| TRIP6 | 0.43933475 | hypo | chr7:100470992-100470993_+ | 3'UTR | 1668 | 0.02067046 |
| RBMXL1 | 0.43968268 | hypo | chr1:89449128-89449129_- | CDS | 996 | 0.02839175 |
| CHD2 | 0.43975923 | hypo | chr15:93570773-93570774_+ | 3'UTR | 8900 | 0.04611813 |
| NCOA1 | 0.44003877 | hypo | chr2:24930476-24930477_+ | CDS | 2881 | 0.03542228 |
| PRRC2B | 0.44021277 | hypo | chr9:134374410-134374411_+ | 3'UTR | 9894 | 0.04459649 |
| RBBP6 | 0.44405834 | hypo | chr16:24583172-24583173_+ | CDS | 5825 | 0.03301471 |
| STX18 | 0.44436928 | hypo | chr4:4421505-4421506_- | 3'UTR | 1364 | 0.0144825 |
| YTHDF2 | 0.44501176 | hypo | chr1:29070176-29070177_+ | CDS | 1657 | 0.04972127 |
| PXMP2 | 0.44578295 | hypo | chr12:133281287-133281288_+ | 3'UTR | 667 | 0.00169784 |
| CHML | 0.44583491 | hypo | chr1:241797666-241797667_- | CDS | 1565 | 0.04719344 |
| PLPBP | 0.44589921 | hypo | chr8:37636998-37636999_+ | 3'UTR | 2281 | 0.03265561 |
| DNAJC2 | 0.44615501 | hypo | chr7:102953015-102953016_- | CDS | 2116 | 0.0333651 |
| NFKBIE | 0.4464461 | hypo | chr6:44226524-44226525_- | 3'UTR | 1958 | 0.03476018 |
| HOXB6 | 0.44687026 | hypo | chr17:46675401-46675402_- | CDS | 435 | 0.04136383 |
| STC2 | 0.44929245 | hypo | chr5:172744194-172744195_- | 3'UTR | 2873 | 0.03523644 |
| IGF2BP2 | 0.44965488 | hypo | chr3:185361669-185361670_- | 3'UTR | 3544 | 0.02545596 |
| NMT1 | 0.45036994 | hypo | chr17:43186168-43186169_+ | 3'UTR | 4683 | 0.03301922 |
| SLC1A5 | 0.45162455 | hypo | chr19:47278626-47278627_- | 3'UTR | 2394 | 0.04298242 |
| POLR1B | 0.45186393 | hypo | chr2:113333277-113333278_+ | CDS | 3959 | 0.02114321 |
| PPP2R5E | 0.45233568 | hypo | chr14:63842534-63842535_- | 3'UTR | 2201 | 0.02377481 |
| ZNF623 | 0.45309111 | hypo | chr8:144733729-144733730_+ | 3'UTR | 1776 | 0.04257926 |
| CDK9 | 0.45428422 | hypo | chr9:130552912-130552913_+ | 3'UTR | 2332 | 0.00406531 |
| TRIAP1 | 0.45490482 | hypo | chr12:120882598-120882599_- | 3'UTR | 318 | 0.00514246 |
| GAS2L3 | 0.45509677 | hypo | chr12:101017826-101017827_+ | CDS | 1519 | 0.04109596 |
| NR2F1 | 0.45587286 | hypo | chr5:92929834-92929835_+ | 3'UTR | 3245 | 0.03082208 |
| RNF146 | 0.45616264 | hypo | chr6:127608209-127608210_+ | CDS | 587 | 0.00910683 |
| UVRAG | 0.45644651 | hypo | chr11:75852357-75852358_+ | CDS | 2241 | 0.03806435 |
| CRKL | 0.45830646 | hypo | chr22:21307375-21307376_+ | 3'UTR | 4663 | 0.01273163 |
| LAP3 | 0.45861189 | hypo | chr4:17609252-17609253_+ | 3'UTR | 1762 | 0.02682898 |
| NRBF2 | 0.45863827 | hypo | chr10:64913704-64913705_+ | CDS | 733 | 0.01400283 |
| CTPS1 | 0.45864543 | hypo | chr1:41477744-41477745_+ | 3'UTR | 2347 | 0.02730585 |
| C3orf38 | 0.4592634 | hypo | chr3:88205676-88205677_+ | CDS | 1191 | 0.01072609 |
| SLC35G2 | 0.46018868 | hypo | chr3:136573591-136573592_+ | CDS | 503 | 0.02223644 |
| ALKBH2 | 0.46035008 | hypo | chr12:109530586-109530587_- | CDS | 144 | 0.04324398 |
| EEF1E1 | 0.46038939 | hypo | chr6:8080147-8080148_- | CDS | 526 | 0.01989459 |
| KRI1 | 0.46205865 | hypo | chr19:10671920-10671921_- | CDS | 519 | 0.00499064 |
| SMG1 | 0.4625758 | hypo | chr16:18820850-18820851_- | 3'UTR | 11388 | 0.04366559 |
| KANSL3 | 0.46260522 | hypo | chr2:97260360-97260361_- | 3'UTR | 3770 | 0.03790291 |
| PPP1CB | 0.46272262 | hypo | chr2:29023636-29023637_+ | 3'UTR | 2611 | 0.01786576 |
| CTTNBP2NL | 0.46311408 | hypo | chr1:112999053-112999054_+ | CDS | 1167 | 0.01301186 |
| SNU13 | 0.4634929 | hypo | chr22:42070218-42070219_- | 3'UTR | 1217 | 0.01864686 |
| ARL4D | 0.46411093 | hypo | chr17:41477379-41477380_+ | CDS | 452 | 0.01947498 |
| H1F0 | 0.46460565 | hypo | chr22:38201639-38201640_+ | CDS | 526 | 0.04424621 |
| TM9SF2 | 0.46489855 | hypo | chr13:100215359-100215360_+ | 3'UTR | 2572 | 0.02632595 |
| RND3 | 0.46557589 | hypo | chr2:151326528-151326529_- | CDS | 984 | 0.02533896 |
| TAZ | 0.46566181 | hypo | chrX:153649432-153649433_+ | 3'UTR | 1295 | 0.01037003 |
| PCNX3 | 0.46592319 | hypo | chr11:65386100-65386101_+ | CDS | 1267 | 0.00816652 |
| MECP2 | 0.46616738 | hypo | chrX:153290676-153290677_- | 3'UTR | 6827 | 0.0330767 |
| CAPN7 | 0.46667876 | hypo | chr3:15292805-15292806_+ | 3'UTR | 2750 | 0.03908225 |
| CHCHD3 | 0.46699158 | hypo | chr7:132470183-132470184_- | 3'UTR | 1118 | 0.04283168 |
| FBLN1 | 0.46701986 | hypo | chr22:45927169-45927170_+ | CDS | 656 | 0.0261422 |
| LATS2 | 0.46738587 | hypo | chr13:21557853-21557854_- | CDS | 2432 | 0.00526012 |
| ACO2 | 0.46794354 | hypo | chr22:41924961-41924962_+ | 3'UTR | 2709 | 0.02424861 |
| NADK2 | 0.46852708 | hypo | chr5:36195078-36195079_- | 3'UTR | 1622 | 0.04804088 |
| SPPL3 | 0.46854647 | hypo | chr12:121202468-121202469_- | 3'UTR | 1972 | 0.03898911 |
| MRPS14 | 0.46863057 | hypo | chr1:174983647-174983648_- | 3'UTR | 590 | 0.01611293 |
| SNW1 | 0.46985659 | hypo | chr14:78184514-78184515_- | CDS | 1553 | 0.04866914 |
| POU2F1 | 0.46998766 | hypo | chr1:167385270-167385271_+ | 3'UTR | 2593 | 0.00469165 |
| TMEM248 | 0.47037618 | hypo | chr7:66420633-66420634_+ | 3'UTR | 1333 | 0.00838489 |
| MPV17L2 | 0.47112188 | hypo | chr19:18304176-18304177_+ | CDS | 137 | 0.02651303 |
| TRMT61B | 0.47115324 | hypo | chr2:29092619-29092620_- | CDS | 555 | 0.03005585 |
| NAA50 | 0.47120811 | hypo | chr3:113439630-113439631_- | 3'UTR | 1835 | 0.02403206 |
| OXA1L | 0.47191613 | hypo | chr14:23240908-23240909_+ | 3'UTR | 1463 | 0.04934058 |
| S100PBP | 0.47232012 | hypo | chr1:33292100-33292101_+ | CDS | 691 | 0.04391457 |
| RPLP0 | 0.4727116 | hypo | chr12:120637169-120637170_- | CDS | 349 | 0.02128141 |
| RNF169 | 0.47310374 | hypo | chr11:74553367-74553368_+ | 3'UTR | 7732 | 0.03965446 |
| CELSR3 | 0.47384589 | hypo | chr3:48699268-48699269_- | CDS | 1079 | 0.04463447 |
| RIF1 | 0.47406916 | hypo | chr2:152320592-152320593_+ | CDS | 4719 | 0.01170077 |
| SRA1 | 0.47492205 | hypo | chr5:139929854-139929855_- | 3'UTR | 1060 | 0.00294822 |
| DHODH | 0.47536265 | hypo | chr16:72058100-72058101_+ | 3'UTR | 1211 | 0.02983449 |
| ANKS6 | 0.47561397 | hypo | chr9:101494663-101494664_- | 3'UTR | 6803 | 0.03213938 |
| HIP1 | 0.47699929 | hypo | chr7:75165632-75165633_- | 3'UTR | 5025 | 0.04179911 |
| PEX26 | 0.47712803 | hypo | chr22:18571033-18571034_+ | 3'UTR | 1319 | 0.00207018 |
| FAM104A | 0.47893277 | hypo | chr17:71205634-71205635_- | 3'UTR | 744 | 0.02239778 |
| TAF7 | 0.47904137 | hypo | chr5:140698193-140698194_- | 3'UTR | 2157 | 0.04485321 |
| SRFBP1 | 0.48045333 | hypo | chr5:121356136-121356137_+ | CDS | 778 | 0.03442782 |
| DDX19B | 0.48049322 | hypo | chr16:70367634-70367635_+ | 3'UTR | 1834 | 0.02584906 |
| GOSR1 | 0.48064625 | hypo | chr17:28849400-28849401_+ | 3'UTR | 783 | 0.00739897 |
| PTPMT1 | 0.4807028 | hypo | chr11:47593175-47593176_+ | CDS | 793 | 0.02250397 |
| BDNF | 0.48127149 | hypo | chr11:27679749-27679750_- | CDS | 748 | 0.00731999 |
| NSD1 | 0.48188004 | hypo | chr5:176637126-176637127_+ | CDS | 1864 | 0.04171424 |
| DDX6 | 0.48215815 | hypo | chr11:118656964-118656965_- | 5'UTR | 356 | 0.04378992 |
| SPOP | 0.48265884 | hypo | chr17:47677435-47677436_- | 3'UTR | 1725 | 0.04135008 |
| EIF4EBP1 | 0.48296759 | hypo | chr8:37914660-37914661_+ | CDS | 279 | 0.02582772 |
| H19 | 0.48303511 | hypo | chr11:2017186-2017187_- | 1647 | 1647 | 0.0300171 |
| SSR2 | 0.48322373 | hypo | chr1:155979115-155979116_- | 3'UTR | 846 | 0.0403141 |
| BLMH | 0.48352978 | hypo | chr17:28575980-28575981_- | 3'UTR | 1546 | 0.02599049 |
| DDX19A | 0.48381692 | hypo | chr16:70406532-70406533_+ | 3'UTR | 2369 | 0.04871301 |
| WDR33 | 0.48511468 | hypo | chr2:128477828-128477829_- | CDS | 1968 | 0.0109711 |
| DCP1A | 0.48578635 | hypo | chr3:53321519-53321520_- | 3'UTR | 1936 | 0.0039544 |
| RILPL1 | 0.48639413 | hypo | chr12:123956932-123956933_- | 3'UTR | 1599 | 0.01416964 |
| CCDC47 | 0.48695784 | hypo | chr17:61829773-61829774_- | CDS | 1444 | 0.02356387 |
| DDX21 | 0.48708202 | hypo | chr10:70742626-70742627_+ | 3'UTR | 2513 | 0.02229402 |
| COX7A2L | 0.4872262 | hypo | chr2:42578365-42578366_- | CDS | 419 | 0.03183207 |
| FLYWCH1 | 0.48726611 | hypo | chr16:2998919-2998920_+ | 3'UTR | 2702 | 0.03843803 |
| SRRD | 0.48743752 | hypo | chr22:26887743-26887744_+ | 3'UTR | 1132 | 0.01418335 |
| BTG1 | 0.4882121 | hypo | chr12:92537964-92537965_- | CDS | 768 | 0.04617028 |
| PEAK1 | 0.48834779 | hypo | chr15:77425791-77425792_- | CDS | 4207 | 0.04853915 |
| TMEM44-AS1 | 0.48907317 | hypo | chr3:194310761-194310762_+ | 634 | 634 | 0.00051224 |
| ZKSCAN8 | 0.48936925 | hypo | chr6:28121226-28121227_+ | CDS | 1380 | 0.02905509 |
| G2E3 | 0.48936954 | hypo | chr14:31085609-31085610_+ | CDS | 2109 | 0.027465 |
| GALNT10 | 0.48950714 | hypo | chr5:153800159-153800160_+ | 3'UTR | 5571 | 0.02991932 |
| NUCKS1 | 0.48954198 | hypo | chr1:205687035-205687036_- | 3'UTR | 1374 | 0.02616566 |
| IGFBP7 | 0.49066774 | hypo | chr4:57897384-57897385_- | 3'UTR | 980 | 0.03929765 |
| EBNA1BP2 | 0.4920509 | hypo | chr1:43630018-43630019_- | 3'UTR | 1329 | 0.0090024 |
| ZNF304 | 0.49269939 | hypo | chr19:57868195-57868196_+ | CDS | 1349 | 0.04553141 |
| DOCK3 | 0.49303656 | hypo | chr3:51418631-51418632_+ | CDS | 5757 | 0.01467308 |
| CACUL1 | 0.49430331 | hypo | chr10:120445601-120445602_- | 3'UTR | 1355 | 0.04996421 |
| SDF2 | 0.49463869 | hypo | chr17:26975623-26975624_- | 3'UTR | 1314 | 0.01908299 |
| EMILIN2 | 0.49500321 | hypo | chr18:2913631-2913632_+ | 3'UTR | 3550 | 0.02190115 |
| USP4 | 0.49553891 | hypo | chr3:49315624-49315625_- | 3'UTR | 3071 | 0.02440595 |
| INPP5D | 0.49581589 | hypo | chr2:234115770-234115771_+ | 3'UTR | 4410 | 0.02413505 |
| ZNF507 | 0.49582017 | hypo | chr19:32874111-32874112_+ | 3'UTR | 3162 | 0.03560994 |
| ARL4C | 0.49582254 | hypo | chr2:235404542-235404543_- | 3'UTR | 1150 | 0.00992721 |
| NSMCE3 | 0.49582404 | hypo | chr15:29560967-29560968_- | 3'UTR | 1052 | 0.04538517 |
| SPOP | 0.49584177 | hypo | chr17:47677408-47677409_- | 3'UTR | 1752 | 0.04645819 |
| NCR3LG1 | 0.49629902 | hypo | chr11:17398355-17398356_+ | 3'UTR | 5869 | 0.03745733 |
| AASDH | 0.49635293 | hypo | chr4:57204699-57204700_- | CDS | 3353 | 0.03329219 |
| PVR | 0.4964749 | hypo | chr19:45166499-45166500_+ | 3'UTR | 2974 | 0.03504403 |
| LATS2 | 0.49796472 | hypo | chr13:21548781-21548782_- | 3'UTR | 3935 | 0.00682335 |
| CCT5 | 0.49921822 | hypo | chr5:10258557-10258558_+ | CDS | 1203 | 0.01880335 |
| RAB9A | 0.49987017 | hypo | chrX:13727049-13727050_+ | CDS | 378 | 0.03688443 |
| TANK | 0.50161203 | hypo | chr2:162087784-162087785_+ | CDS | 981 | 0.03429744 |
| SIAH2 | 0.50173947 | hypo | chr3:150460197-150460198_- | CDS | 1003 | 0.00463685 |
| DDHD1 | 0.50181281 | hypo | chr14:53513471-53513472_- | 3'UTR | 2946 | 0.01850648 |
| GRB10 | 0.50190406 | hypo | chr7:50660297-50660298_- | 3'UTR | 2898 | 0.03777956 |
| PWWP2A | 0.50204769 | hypo | chr5:159520676-159520677_- | CDS | 1036 | 0.04172624 |
| RNF169 | 0.50304588 | hypo | chr11:74553326-74553327_+ | 3'UTR | 7691 | 0.02378307 |
| PHF5A | 0.50431575 | hypo | chr22:41855952-41855953_- | 3'UTR | 832 | 0.02007039 |
| TGIF1 | 0.50484864 | hypo | chr18:3457954-3457955_+ | 3'UTR | 1797 | 0.01720345 |
| RPL3 | 0.5050563 | hypo | chr22:39713589-39713590_- | CDS | 266 | 0.04818022 |
| ERP29 | 0.50645045 | hypo | chr12:112460406-112460407_+ | CDS | 854 | 0.02307564 |
| ZNF384 | 0.50795963 | hypo | chr12:6776092-6776093_- | 3'UTR | 2790 | 0.0202323 |
| PEAK1 | 0.50826109 | hypo | chr15:77425634-77425635_- | CDS | 4364 | 0.00278158 |
| NFKBIE | 0.5088048 | hypo | chr6:44226742-44226743_- | 3'UTR | 1740 | 0.03400595 |
| CITED2 | 0.5096433 | hypo | chr6:139694146-139694147_- | 3'UTR | 1024 | 0.00946059 |
| DHX36 | 0.50988178 | hypo | chr3:153993924-153993925_- | 3'UTR | 3132 | 0.04569645 |
| PHF3 | 0.51007624 | hypo | chr6:64395566-64395567_+ | CDS | 1969 | 0.01912984 |
| SP3 | 0.51068655 | hypo | chr2:174774817-174774818_- | CDS | 2727 | 0.03653469 |
| TIGD2 | 0.51094944 | hypo | chr4:90035526-90035527_+ | CDS | 1559 | 0.036848 |
| ZNF133 | 0.51120479 | hypo | chr20:18297474-18297475_+ | 3'UTR | 2372 | 0.01721333 |
| PELO | 0.51139499 | hypo | chr5:52097695-52097696_+ | 3'UTR | 2164 | 0.02832875 |
| MATR3 | 0.51156759 | hypo | chr5:138643495-138643496_+ | CDS | 730 | 0.00396123 |
| ZSWIM1 | 0.51164055 | hypo | chr20:44511300-44511301_+ | CDS | 182 | 0.02629282 |
| THUMPD1 | 0.51302572 | hypo | chr16:20745577-20745578_- | 3'UTR | 3910 | 0.03402678 |
| MGA | 0.51392904 | hypo | chr15:42042608-42042609_+ | CDS | 6984 | 0.04137552 |
| KDM2A | 0.51422706 | hypo | chr11:67024734-67024735_+ | 3'UTR | 6561 | 0.00482161 |
| MID1 | 0.51458511 | hypo | chrX:10417374-10417375_- | 3'UTR | 2229 | 0.00275165 |
| RPLP0 | 0.51483988 | hypo | chr12:120636741-120636742_- | CDS | 556 | 0.02026302 |
| H6PD | 0.51485612 | hypo | chr1:9305258-9305259_+ | CDS | 538 | 0.00862259 |
| CDK13 | 0.51488444 | hypo | chr7:40027346-40027347_+ | CDS | 1746 | 0.0364238 |
| TMEM9B | 0.51742321 | hypo | chr11:8969101-8969102_- | 3'UTR | 1821 | 0.01650445 |
| ARHGAP5 | 0.51827887 | hypo | chr14:32560077-32560078_+ | CDS | 502 | 0.02704127 |
| SLC25A37 | 0.51906089 | hypo | chr8:23423789-23423790_+ | CDS | 587 | 0.00979067 |
| SEMA3A | 0.51917306 | hypo | chr7:83589909-83589910_- | 3'UTR | 3407 | 0.04237354 |
| C1orf43 | 0.52073963 | hypo | chr1:154179950-154179951_- | CDS | 1129 | 0.02329845 |
| RPL37A | 0.5209874 | hypo | chr2:217364725-217364726_+ | CDS | 264 | 0.03989711 |
| TMEM184C | 0.52152682 | hypo | chr4:148555585-148555586_+ | CDS | 1886 | 0.02181094 |
| NARF | 0.52161185 | hypo | chr17:80446122-80446123_+ | 3'UTR | 1523 | 0.01125647 |
| ZNF79 | 0.52161405 | hypo | chr9:130207482-130207483_+ | 3'UTR | 1917 | 0.0077709 |
| SGSH | 0.52232781 | hypo | chr17:78184009-78184010_- | 3'UTR | 1836 | 0.0428705 |
| CCDC93 | 0.52266954 | hypo | chr2:118677412-118677413_- | 3'UTR | 2569 | 0.01207767 |
| FOXQ1 | 0.52317814 | hypo | chr6:1313510-1313511_+ | CDS | 836 | 0.02792516 |
| ARPC5 | 0.52354968 | hypo | chr1:183604729-183604730_- | CDS | 346 | 0.03058184 |
| ZADH2 | 0.52401245 | hypo | chr18:72912845-72912846_- | 3'UTR | 1948 | 0.02956168 |
| BCAS3 | 0.52490607 | hypo | chr17:59469854-59469855_+ | 3'UTR | 3300 | 0.00926165 |
| FOXK2 | 0.52514516 | hypo | chr17:80559915-80559916_+ | 3'UTR | 2694 | 0.01785335 |
| RNMT | 0.52630258 | hypo | chr18:13731676-13731677_+ | CDS | 401 | 0.02181964 |
| ZBTB14 | 0.52643508 | hypo | chr18:5290753-5290754_- | 3'UTR | 1916 | 0.02573898 |
| NIP7 | 0.52729323 | hypo | chr16:69375573-69375574_+ | 3'UTR | 879 | 0.04694798 |
| SARM1 | 0.52738819 | hypo | chr17:26726486-26726487_+ | 3'UTR | 5724 | 0.03713077 |
| ZDHHC16 | 0.52794989 | hypo | chr10:99216820-99216821_+ | 3'UTR | 1534 | 0.00950566 |
| TOM1 | 0.52857419 | hypo | chr22:35728989-35728990_+ | CDS | 1040 | 0.0326324 |
| C1QBP | 0.52891212 | hypo | chr17:5336261-5336262_- | 3'UTR | 999 | 0.03376205 |
| F2R | 0.52893713 | hypo | chr5:76030340-76030341_+ | 3'UTR | 2555 | 0.01286733 |
| EEF2 | 0.52972352 | hypo | chr19:3981413-3981414_- | CDS | 1016 | 0.00905137 |
| ZNF768 | 0.53030825 | hypo | chr16:30537002-30537003_- | CDS | 633 | 0.02787874 |
| FZD6 | 0.53065964 | hypo | chr8:104342176-104342177_+ | CDS | 2125 | 0.00876373 |
| ATMIN | 0.53108668 | hypo | chr16:81077649-81077650_+ | CDS | 1564 | 0.01180278 |
| SCAF11 | 0.53110606 | hypo | chr12:46318836-46318837_- | CDS | 3866 | 0.00664264 |
| PHLPP2 | 0.53156819 | hypo | chr16:71683444-71683445_- | CDS | 3698 | 0.03532076 |
| PPP1R15B | 0.53176566 | hypo | chr1:204372964-204372965_- | 3'UTR | 4802 | 0.04205796 |
| UBE2Q1 | 0.53209428 | hypo | chr1:154522776-154522777_- | 3'UTR | 1496 | 0.01709558 |
| CLIP2 | 0.53223417 | hypo | chr7:73818320-73818321_+ | 3'UTR | 3610 | 0.02989985 |
| GABPB1-AS1 | 0.53241361 | hypo | chr15:50647795-50647796_+ | 1425 | 1425 | 0.04723061 |
| PPP2R5D | 0.53266728 | hypo | chr6:42979684-42979685_+ | 3'UTR | 2648 | 0.01952212 |
| RIC1 | 0.53279369 | hypo | chr9:5763407-5763408_+ | CDS | 2583 | 0.0414078 |
| TUT7 | 0.53305744 | hypo | chr9:88967683-88967684_- | CDS | 678 | 0.04246745 |
| INO80C | 0.53457129 | hypo | chr18:33048500-33048501_- | 3'UTR | 877 | 0.00277598 |
| TAF11 | 0.5348393 | hypo | chr6:34846408-34846409_- | CDS | 707 | 0.00643153 |
| INO80C | 0.5349393 | hypo | chr18:33048534-33048535_- | 3'UTR | 843 | 0.02822522 |
| NUP133 | 0.53563943 | hypo | chr1:229600423-229600424_- | CDS | 2589 | 0.0121181 |
| TGS1 | 0.53592894 | hypo | chr8:56699107-56699108_+ | CDS | 1037 | 0.02236458 |
| ZNF644 | 0.5359506 | hypo | chr1:91406302-91406303_- | CDS | 890 | 0.00781505 |
| CUTC | 0.53619297 | hypo | chr10:101515744-101515745_+ | 3'UTR | 1218 | 0.02855227 |
| C19orf25 | 0.53622274 | hypo | chr19:1474780-1474781_- | 3'UTR | 675 | 0.01081755 |
| FBXO31 | 0.53632789 | hypo | chr16:87364667-87364668_- | 3'UTR | 1894 | 0.00953844 |
| TICAM1 | 0.53691973 | hypo | chr19:4816093-4816094_- | 3'UTR | 2563 | 0.0033078 |
| MED19 | 0.53696654 | hypo | chr11:57471427-57471428_- | 3'UTR | 1286 | 0.03148048 |
| NFYA | 0.53700707 | hypo | chr6:41066691-41066692_+ | 3'UTR | 2764 | 0.01287198 |
| COL18A1 | 0.53732158 | hypo | chr21:46933482-46933483_+ | 3'UTR | 5730 | 0.01076707 |
| TBC1D14 | 0.53739387 | hypo | chr4:7034270-7034271_+ | 3'UTR | 4434 | 0.04577375 |
| ING5 | 0.53838006 | hypo | chr2:242664589-242664590_+ | 3'UTR | 913 | 0.00631496 |
| NSF | 0.53888495 | hypo | chr17:44834389-44834390_+ | 3'UTR | 3542 | 0.04343062 |
| LATS2 | 0.53889743 | hypo | chr13:21562317-21562318_- | CDS | 2042 | 0.02593934 |
| ILF3 | 0.53912794 | hypo | chr19:10801528-10801529_+ | 3'UTR | 4554 | 0.03029018 |
| XRCC5 | 0.53943134 | hypo | chr2:216981523-216981524_+ | CDS | 366 | 0.02125499 |
| ZNF324 | 0.54011634 | hypo | chr19:58984098-58984099_+ | 3'UTR | 2384 | 0.01464854 |
| MARCH8 | 0.54055034 | hypo | chr10:45953832-45953833_- | CDS | 991 | 0.03796905 |
| PUS3 | 0.54076652 | hypo | chr11:125763672-125763673_- | 3'UTR | 1550 | 0.04207539 |
| TARS | 0.54081269 | hypo | chr5:33459960-33459961_+ | CDS | 1654 | 0.01400737 |
| ASXL1 | 0.54137024 | hypo | chr20:31023090-31023091_+ | CDS | 3007 | 0.01120097 |
| TRIM25 | 0.54147695 | hypo | chr17:54965456-54965457_- | 3'UTR | 5556 | 0.04569815 |
| C12orf65 | 0.54189 | hypo | chr12:123738421-123738422_+ | CDS | 463 | 0.03657841 |
| CD2BP2 | 0.5419763 | hypo | chr16:30364988-30364989_- | CDS | 683 | 0.03129563 |
| MPHOSPH8 | 0.54201623 | hypo | chr13:20221312-20221313_+ | CDS | 1200 | 0.03456323 |
| ANKRD11 | 0.54217165 | hypo | chr16:89348042-89348043_- | CDS | 5364 | 0.00959888 |
| C11orf68 | 0.54234455 | hypo | chr11:65684416-65684417_- | 3'UTR | 1423 | 0.03005265 |
| SCAF8 | 0.54258202 | hypo | chr6:155153407-155153408_+ | CDS | 3217 | 0.01236248 |
| THAP11 | 0.54269276 | hypo | chr16:67877775-67877776_+ | 3'UTR | 1563 | 0.02696385 |
| PRPF38B | 0.54293733 | hypo | chr1:109242467-109242468_+ | CDS | 1748 | 0.01478326 |
| APC | 0.54402339 | hypo | chr5:112179399-112179400_+ | CDS | 8167 | 0.00958224 |
| XBP1 | 0.54441769 | hypo | chr22:29191205-29191206_- | CDS | 1135 | 0.03028476 |
| GCNT1 | 0.54510977 | hypo | chr9:79118478-79118479_+ | CDS | 1624 | 0.0259033 |
| NSMCE3 | 0.5453859 | hypo | chr15:29561910-29561911_- | 5'UTR | 109 | 0.04451381 |
| PCOLCE | 0.54662756 | hypo | chr7:100203338-100203339_+ | CDS | 722 | 0.02684877 |
| SHOC2 | 0.54687368 | hypo | chr10:112773007-112773008_+ | 3'UTR | 3529 | 0.0019944 |
| PDE4D | 0.547494 | hypo | chr5:58270574-58270575_- | CDS | 2517 | 0.03503951 |
| SLC7A1 | 0.54768751 | hypo | chr13:30088106-30088107_- | 3'UTR | 2786 | 0.04332545 |
| POLR1D | 0.54785324 | hypo | chr13:28239912-28239913_+ | CDS | 396 | 0.04407264 |
| ZC3H4 | 0.54865638 | hypo | chr19:47568484-47568485_- | 3'UTR | 5077 | 0.02511553 |
| JTB | 0.54886294 | hypo | chr1:153949925-153949926_- | 5'UTR | 525 | 0.02496381 |
| AFF4 | 0.5488906 | hypo | chr5:132232626-132232627_- | CDS | 2074 | 0.04216499 |
| IFIT5 | 0.54889623 | hypo | chr10:91176975-91176976_+ | CDS | 250 | 0.02880761 |
| KLF6 | 0.54892608 | hypo | chr10:3819652-3819653_- | 3'UTR | 3124 | 0.04661384 |
| NUP50 | 0.54895583 | hypo | chr22:45581481-45581482_+ | 3'UTR | 2734 | 0.01224433 |
| PRPSAP1 | 0.54909933 | hypo | chr17:74307641-74307642_- | CDS | 1584 | 0.02897213 |
| EIF1 | 0.54916162 | hypo | chr17:39847446-39847447_+ | 3'UTR | 874 | 0.01665809 |
| LINC00649 | 0.54973735 | hypo | chr21:35341565-35341566_+ | 735 | 735 | 0.03625674 |
| FAM193B | 0.54997907 | hypo | chr5:176951988-176951989_- | CDS | 1619 | 0.00685088 |
| POP5 | 0.55014596 | hypo | chr12:121016903-121016904_- | 3'UTR | 740 | 0.04120484 |
| BTBD6 | 0.55022563 | hypo | chr14:105716452-105716453_+ | CDS | 1009 | 0.02418849 |
| TSSC4 | 0.55085989 | hypo | chr11:2424537-2424538_+ | CDS | 1165 | 0.02709345 |
| ZNF451 | 0.5515819 | hypo | chr6:56965613-56965614_+ | CDS | 643 | 0.01787297 |
| TAF12 | 0.5520558 | hypo | chr1:28930026-28930027_- | 3'UTR | 694 | 0.0114474 |
| CLEC16A | 0.55242264 | hypo | chr16:11274595-11274596_+ | 3'UTR | 5364 | 0.00868465 |
| RBM4B | 0.55291244 | hypo | chr11:66436598-66436599_- | CDS | 711 | 0.01410907 |
| IL21R | 0.55321312 | hypo | chr16:27460116-27460117_+ | CDS | 1249 | 0.00948685 |
| LOC100130283 | 0.55334322 | hypo | chr16:8953995-8953996_+ | 645 | 645 | 0.03693737 |
| PPP2R3A | 0.55360135 | hypo | chr3:135722079-135722080_+ | CDS | 2301 | 0.01116376 |
| TMEM198B | 0.55368728 | hypo | chr12:56224067-56224068_+ | 542 | 542 | 0.02873304 |
| ZBTB38 | 0.55413087 | hypo | chr3:141164667-141164668_+ | CDS | 4419 | 0.04362832 |
| CCDC127 | 0.55444434 | hypo | chr5:205831-205832_- | CDS | 462 | 0.00133603 |
| CAMSAP2 | 0.55456717 | hypo | chr1:200827211-200827212_+ | 3'UTR | 4731 | 0.02498133 |
| SGPP1 | 0.55470957 | hypo | chr14:64194126-64194127_- | CDS | 686 | 0.03612061 |
| GFOD2 | 0.55492446 | hypo | chr16:67709067-67709068_- | CDS | 1388 | 0.04704462 |
| KHDC4 | 0.55531748 | hypo | chr1:155883126-155883127_- | 3'UTR | 2669 | 0.0248496 |
| MRM3 | 0.55543153 | hypo | chr17:695084-695085_+ | CDS | 1064 | 0.01713756 |
| ZNF3 | 0.5554765 | hypo | chr7:99669570-99669571_- | CDS | 862 | 0.01600711 |
| C1QBP | 0.55582105 | hypo | chr17:5336283-5336284_- | 3'UTR | 977 | 0.0220263 |
| SLC5A6 | 0.55605077 | hypo | chr2:27423206-27423207_- | 3'UTR | 2499 | 0.00361081 |
| ELK1 | 0.5565731 | hypo | chrX:47496150-47496151_- | 3'UTR | 1579 | 0.02694792 |
| ZNF687 | 0.55668242 | hypo | chr1:151258999-151259000_+ | CDS | 333 | 0.02798714 |
| BDNF | 0.55675027 | hypo | chr11:27679686-27679687_- | CDS | 811 | 0.00666812 |
| B4GAT1 | 0.55709608 | hypo | chr11:66114330-66114331_- | CDS | 769 | 0.02348985 |
| NCOA5 | 0.55749518 | hypo | chr20:44689992-44689993_- | 3'UTR | 2860 | 0.02232567 |
| ZNF623 | 0.55864778 | hypo | chr8:144734474-144734475_+ | 3'UTR | 2521 | 0.01684426 |
| ATL3 | 0.55868167 | hypo | chr11:63398877-63398878_- | CDS | 1567 | 0.02811211 |
| ZNF317 | 0.558789 | hypo | chr19:9272841-9272842_+ | 3'UTR | 2808 | 0.03202866 |
| SON | 0.55899684 | hypo | chr21:34926929-34926930_+ | CDS | 5447 | 0.02727951 |
| EDC3 | 0.55900406 | hypo | chr15:74925129-74925130_- | CDS | 1541 | 0.00643928 |
| ATG2B | 0.55952963 | hypo | chr14:96751982-96751983_- | 3'UTR | 6770 | 0.04104026 |
| ATMIN | 0.55995979 | hypo | chr16:81078461-81078462_+ | CDS | 2376 | 0.03746013 |
| PBRM1 | 0.56006575 | hypo | chr3:52643781-52643782_- | CDS | 2227 | 0.03082329 |
| ABCF1 | 0.56068438 | hypo | chr6:30545212-30545213_+ | CDS | 195 | 0.03597324 |
| STAM | 0.56097988 | hypo | chr10:17756824-17756825_+ | 3'UTR | 1857 | 0.0048081 |
| TRMT10C | 0.56181395 | hypo | chr3:101284764-101284765_+ | CDS | 1319 | 0.00768289 |
| LCORL | 0.56299135 | hypo | chr4:17886177-17886178_- | CDS | 1097 | 0.0025973 |
| ASNSD1 | 0.56380801 | hypo | chr2:190531129-190531130_+ | CDS | 705 | 0.03649202 |
| SRRD | 0.56499288 | hypo | chr22:26887606-26887607_+ | CDS | 995 | 0.04206016 |
| DDX17 | 0.56713559 | hypo | chr22:38879945-38879946_- | 3'UTR | 4292 | 0.02904878 |
| C12orf43 | 0.56748413 | hypo | chr12:121441159-121441160_- | 3'UTR | 1612 | 0.04615981 |
| ZCCHC8 | 0.5678263 | hypo | chr12:122958601-122958602_- | CDS | 1798 | 0.00648019 |
| ABCB6 | 0.56847494 | hypo | chr2:220074659-220074660_- | CDS | 2872 | 0.03009968 |
| PRRC2C | 0.56886489 | hypo | chr1:171501723-171501724_+ | CDS | 1756 | 0.04138584 |
| TUT4 | 0.56933908 | hypo | chr1:52940682-52940683_- | CDS | 2802 | 0.02887913 |
| SZRD1 | 0.56955548 | hypo | chr1:16721715-16721716_+ | 3'UTR | 767 | 0.02853695 |
| SHISA5 | 0.56985707 | hypo | chr3:48509466-48509467_- | 3'UTR | 1771 | 0.01652811 |
| BHLHE40 | 0.56997649 | hypo | chr3:5025243-5025244_+ | CDS | 1412 | 0.03887268 |
| FAM126A | 0.57024322 | hypo | chr7:22985549-22985550_- | CDS | 1478 | 0.01404204 |
| DDX28 | 0.57069455 | hypo | chr16:68055448-68055449_- | 3'UTR | 2321 | 0.04960839 |
| FAM53B | 0.57127528 | hypo | chr10:126370699-126370700_- | CDS | 904 | 0.03924511 |
| PHLDA1 | 0.57153111 | hypo | chr12:76424293-76424294_- | 3'UTR | 1262 | 0.02679837 |
| ABL2 | 0.57163703 | hypo | chr1:179077156-179077157_- | CDS | 3244 | 0.02740931 |
| CDYL | 0.57165629 | hypo | chr6:4892316-4892317_+ | CDS | 732 | 0.0072068 |
| PYCR1 | 0.57190842 | hypo | chr17:79890968-79890969_- | 3'UTR | 1523 | 0.00827005 |
| ACTR8 | 0.57196218 | hypo | chr3:53902711-53902712_- | 3'UTR | 1959 | 0.00984186 |
| RPP38 | 0.57216625 | hypo | chr10:15145941-15145942_+ | CDS | 868 | 0.04918765 |
| LOC100129034 | 0.57219486 | hypo | chr9:127116803-127116804_+ | 1052 | 1052 | 0.0273478 |
| KLHL21 | 0.57225417 | hypo | chr1:6659202-6659203_- | CDS | 1411 | 0.02169525 |
| RPP25L | 0.57289529 | hypo | chr9:34611012-34611013_- | CDS | 366 | 0.00823048 |
| EPS8 | 0.57308846 | hypo | chr12:15776153-15776154_- | CDS | 2729 | 0.0145449 |
| TRMT2A | 0.57329364 | hypo | chr22:20103816-20103817_- | CDS | 731 | 0.00994723 |
| EXOC3 | 0.57371002 | hypo | chr5:466969-466970_+ | CDS | 2322 | 0.04361721 |
| DUSP3 | 0.57414748 | hypo | chr17:41845774-41845775_- | 3'UTR | 1812 | 0.02154703 |
| BCL2L1 | 0.57427293 | hypo | chr20:30253863-30253864_- | CDS | 1200 | 0.02414518 |
| BLOC1S3 | 0.5750549 | hypo | chr19:45684751-45684752_+ | 3'UTR | 2293 | 0.02737712 |
| RPS25 | 0.57509208 | hypo | chr11:118888202-118888203_- | CDS | 214 | 0.03080071 |
| NXT1 | 0.57556114 | hypo | chr20:23335183-23335184_+ | 3'UTR | 892 | 0.01626634 |
| PKM | 0.57608219 | hypo | chr15:72491470-72491471_- | 3'UTR | 2974 | 0.02512908 |
| RPS4X | 0.57631715 | hypo | chrX:71492503-71492504_- | 3'UTR | 904 | 0.00948343 |
| CHD4 | 0.57668836 | hypo | chr12:6688016-6688017_- | CDS | 5230 | 0.03802672 |
| CDR2 | 0.57704654 | hypo | chr16:22357719-22357720_- | 3'UTR | 2240 | 0.00483757 |
| CCDC86 | 0.5778539 | hypo | chr11:60617975-60617976_+ | 3'UTR | 1429 | 0.00842077 |
| DYNLL2 | 0.57790967 | hypo | chr17:56166796-56166797_+ | 3'UTR | 700 | 0.01435876 |
| ZNF410 | 0.57843894 | hypo | chr14:74398438-74398439_+ | 3'UTR | 2250 | 0.02627596 |
| RIPOR1 | 0.5785383 | hypo | chr16:67576764-67576765_+ | CDS | 2321 | 0.01824778 |
| UTP3 | 0.57892658 | hypo | chr4:71555544-71555545_+ | CDS | 1349 | 0.03444201 |
| RAC1 | 0.57908789 | hypo | chr7:6443271-6443272_+ | 3'UTR | 2071 | 0.02625337 |
| DEAF1 | 0.57919366 | hypo | chr11:644339-644340_- | 3'UTR | 2614 | 0.01983121 |
| EEF2 | 0.57958676 | hypo | chr19:3976396-3976397_- | 3'UTR | 2814 | 0.01530402 |
| NONO | 0.57979304 | hypo | chrX:70519906-70519907_+ | CDS | 1935 | 0.02701247 |
| MAT2A | 0.5798694 | hypo | chr2:85769724-85769725_+ | CDS | 1115 | 0.04402627 |
| SPPL3 | 0.58005827 | hypo | chr12:121200749-121200750_- | 3'UTR | 3691 | 0.03043574 |
| ZC3H13 | 0.58032551 | hypo | chr13:46549888-46549889_- | CDS | 2086 | 0.04791457 |
| ZNF776 | 0.58042746 | hypo | chr19:58265298-58265299_+ | CDS | 1063 | 0.03627714 |
| USP53 | 0.58045037 | hypo | chr4:120213707-120213708_+ | CDS | 3629 | 0.00677069 |
| HMGN3 | 0.58048645 | hypo | chr6:79911270-79911271_- | 3'UTR | 704 | 0.03018393 |
| MFSD9 | 0.58078116 | hypo | chr2:103335476-103335477_- | CDS | 904 | 0.04725702 |
| ZNF445 | 0.58099018 | hypo | chr3:44487738-44487739_- | 3'UTR | 3771 | 0.00275629 |
| ZRANB1 | 0.58123445 | hypo | chr10:126631657-126631658_+ | CDS | 966 | 0.0231903 |
| NDUFV3 | 0.58161479 | hypo | chr21:44324265-44324266_+ | CDS | 1168 | 0.02222516 |
| URI1 | 0.58204348 | hypo | chr19:30500008-30500009_+ | CDS | 1092 | 0.03282065 |
| TMEM231 | 0.58216314 | hypo | chr16:75572604-75572605_- | 3'UTR | 2411 | 0.02911832 |
| VEGFB | 0.58301865 | hypo | chr11:64005909-64005910_+ | 3'UTR | 978 | 0.03769209 |
| UGGT1 | 0.58304362 | hypo | chr2:128947353-128947354_+ | 3'UTR | 4883 | 0.04189647 |
| FSTL3 | 0.58323257 | hypo | chr19:681717-681718_+ | 3'UTR | 836 | 0.00651941 |
| EIF5B | 0.5834397 | hypo | chr2:99977960-99977961_+ | CDS | 780 | 0.00939602 |
| NCBP2 | 0.58351395 | hypo | chr3:196663733-196663734_- | 3'UTR | 708 | 0.04050699 |
| AFG3L2 | 0.5836615 | hypo | chr18:12329651-12329652_- | CDS | 2499 | 0.03156024 |
| KIAA0930 | 0.583936 | hypo | chr22:45590765-45590766_- | 3'UTR | 3643 | 0.01352206 |
| ROCK2 | 0.58514701 | hypo | chr2:11323507-11323508_- | 3'UTR | 4670 | 0.03123423 |
| EEF2 | 0.58540891 | hypo | chr19:3976229-3976230_- | 3'UTR | 2981 | 0.01754883 |
| BOD1L1 | 0.58552645 | hypo | chr4:13600613-13600614_- | CDS | 8026 | 0.04892783 |
| ETFDH | 0.58553979 | hypo | chr4:159629540-159629541_+ | CDS | 2071 | 0.03824987 |
| DDX23 | 0.58584789 | hypo | chr12:49223781-49223782_- | 3'UTR | 3023 | 0.03483458 |
| AMOTL2 | 0.58597651 | hypo | chr3:134076433-134076434_- | 3'UTR | 2760 | 0.02391502 |
| REST | 0.58619165 | hypo | chr4:57797792-57797793_+ | CDS | 2904 | 0.03229313 |
| CTSC | 0.58621947 | hypo | chr11:88027285-88027286_- | CDS | 1394 | 0.04788099 |
| CDC16 | 0.5862412 | hypo | chr13:115037770-115037771_+ | CDS | 1955 | 0.04805996 |
| ARL4C | 0.58681445 | hypo | chr2:235404285-235404286_- | 3'UTR | 1407 | 0.01519371 |
| RYBP | 0.58687144 | hypo | chr3:72427640-72427641_- | CDS | 551 | 0.0112945 |
| GPATCH8 | 0.58701354 | hypo | chr17:42477679-42477680_- | CDS | 1800 | 0.02694255 |
| PIGM | 0.58708634 | hypo | chr1:160000042-160000043_- | 3'UTR | 1740 | 0.04567487 |
| UPP1 | 0.5873373 | hypo | chr7:48139295-48139296_+ | CDS | 757 | 0.01672394 |
| ASB8 | 0.58735229 | hypo | chr12:48543459-48543460_- | CDS | 725 | 0.04473647 |
| CRKL | 0.58737592 | hypo | chr22:21307518-21307519_+ | 3'UTR | 4806 | 0.01615105 |
| YBX3 | 0.58739405 | hypo | chr12:10852140-10852141_- | 3'UTR | 1508 | 0.00993944 |
| RNF8 | 0.58781853 | hypo | chr6:37358652-37358653_+ | 3'UTR | 1758 | 0.03863212 |
| C11orf68 | 0.58794054 | hypo | chr11:65684648-65684649_- | 3'UTR | 1191 | 0.03914806 |
| GOLGA3 | 0.5880723 | hypo | chr12:133349601-133349602_- | 3'UTR | 5144 | 0.03939748 |
| AEBP1 | 0.58832532 | hypo | chr7:44153972-44153973_+ | 3'UTR | 3910 | 0.00749036 |
| MFSD5 | 0.58852527 | hypo | chr12:53648101-53648102_+ | 3'UTR | 1950 | 0.0488243 |
| NSL1 | 0.58876508 | hypo | chr1:212911837-212911838_- | CDS | 771 | 0.01089281 |
| TCFL5 | 0.5890375 | hypo | chr20:61473295-61473296_- | 3'UTR | 1626 | 0.02110308 |
| DIDO1 | 0.58908953 | hypo | chr20:61542230-61542231_- | CDS | 1045 | 0.01461927 |
| PDE4B | 0.58918502 | hypo | chr1:66838384-66838385_+ | 3'UTR | 2658 | 0.01292641 |
| ZMIZ1 | 0.58934913 | hypo | chr10:81073564-81073565_+ | 3'UTR | 4834 | 0.04428993 |
| HERPUD1 | 0.5897555 | hypo | chr16:56977282-56977283_+ | 3'UTR | 1359 | 0.03692099 |
| SSH2 | 0.5898091 | hypo | chr17:27959198-27959199_- | CDS | 2971 | 0.02136999 |
| RPS11 | 0.58999006 | hypo | chr19:50002823-50002824_+ | CDS | 500 | 0.02275637 |
| PPP1R15A | 0.59017518 | hypo | chr19:49376743-49376744_+ | CDS | 522 | 0.03433095 |
| KLHL42 | 0.59040241 | hypo | chr12:27950881-27950882_+ | CDS | 1377 | 0.03593493 |
| MAP3K20 | 0.59072557 | hypo | chr2:174131353-174131354_+ | CDS | 2478 | 0.02653744 |
| RBM15 | 0.59091554 | hypo | chr1:110884137-110884138_+ | CDS | 2193 | 0.00398726 |
| ARL6IP5 | 0.59104617 | hypo | chr3:69153787-69153788_+ | CDS | 678 | 0.03118906 |
| DNAJC9 | 0.59133783 | hypo | chr10:75005706-75005707_- | CDS | 614 | 0.04982725 |
| CUEDC1 | 0.59181583 | hypo | chr17:55940549-55940550_- | 3'UTR | 1674 | 0.00764079 |
| SNX33 | 0.59292484 | hypo | chr15:75942578-75942579_+ | CDS | 1231 | 0.03279706 |
| SLC3A2 | 0.59376495 | hypo | chr11:62656175-62656176_+ | 3'UTR | 2161 | 0.02640639 |
| ABL1 | 0.59412011 | hypo | chr9:133761705-133761706_+ | 3'UTR | 4524 | 0.03287806 |
| GTF3C1 | 0.5943358 | hypo | chr16:27475759-27475760_- | CDS | 5792 | 0.02747209 |
| CNNM4 | 0.59438984 | hypo | chr2:97476010-97476011_+ | 3'UTR | 3182 | 0.03182232 |
| RAP2C | 0.59458734 | hypo | chrX:131351126-131351127_- | CDS | 953 | 0.02247197 |
| KHNYN | 0.59510725 | hypo | chr14:24907490-24907491_+ | 3'UTR | 3238 | 0.04362308 |
| MAD2L1BP | 0.59525697 | hypo | chr6:43608246-43608247_+ | CDS | 1105 | 0.04044176 |
| SLC35A4 | 0.59601009 | hypo | chr5:139946302-139946303_+ | 5'UTR | 546 | 0.03093599 |
| CCDC71 | 0.59621309 | hypo | chr3:49200779-49200780_- | CDS | 999 | 0.00918444 |
| KLHL22 | 0.59636043 | hypo | chr22:20796525-20796526_- | CDS | 1895 | 0.04051793 |
| SEL1L | 0.59646203 | hypo | chr14:81943286-81943287_- | 3'UTR | 2530 | 0.03098771 |
| PA2G4 | 0.59689414 | hypo | chr12:56507506-56507507_+ | 3'UTR | 2438 | 0.03387236 |
| LSM10 | 0.59691051 | hypo | chr1:36859599-36859600_- | CDS | 347 | 0.04255216 |
| NCBP2-AS2 | 0.59731387 | hypo | chr3:196670237-196670238_+ | 3'UTR | 744 | 0.01882341 |
| DAP3 | 0.59757855 | hypo | chr1:155708152-155708153_+ | 3'UTR | 1404 | 0.02389975 |
| CAMKK1 | 0.59769216 | hypo | chr17:3764649-3764650_- | 3'UTR | 2466 | 0.00167407 |
| ADAMTS1 | 0.59782999 | hypo | chr21:28210513-28210514_- | CDS | 2742 | 0.03114764 |
| NPEPPS | 0.59936637 | hypo | chr17:45699405-45699406_+ | 3'UTR | 3102 | 0.03832227 |
| FBLN1 | 0.59944412 | hypo | chr22:45914601-45914602_+ | CDS | 266 | 0.02044658 |
| BRD2 | 0.5996532 | hypo | chr6:32944675-32944676_+ | CDS | 1179 | 0.03868395 |
| SP4 | 0.59967721 | hypo | chr7:21550703-21550704_+ | CDS | 2340 | 0.00750741 |
| ZMIZ1 | 0.59997386 | hypo | chr10:81072660-81072661_+ | 3'UTR | 3930 | 0.01775206 |
| CAPN7 | 0.6012912 | hypo | chr3:15292700-15292701_+ | CDS | 2645 | 0.04358238 |
| TBL1X | 0.60141494 | hypo | chrX:9686600-9686601_+ | 3'UTR | 4535 | 0.0183761 |
| TACC1 | 0.60144279 | hypo | chr8:38677899-38677900_+ | CDS | 1516 | 0.04824424 |
| HDLBP | 0.60153426 | hypo | chr2:242192834-242192835_- | CDS | 1493 | 0.01692891 |
| GPX8 | 0.60158179 | hypo | chr5:54460416-54460417_+ | 3'UTR | 1075 | 0.02570497 |
| CDK12 | 0.60207931 | hypo | chr17:37627726-37627727_+ | CDS | 2202 | 0.03189116 |
| NEU1 | 0.60212664 | hypo | chr6:31827222-31827223_- | 3'UTR | 1676 | 0.01090943 |
| MEX3A | 0.60213205 | hypo | chr1:156046917-156046918_- | CDS | 1009 | 0.00484832 |
| CTSD | 0.60278343 | hypo | chr11:1774850-1774851_- | CDS | 1188 | 0.02526514 |
| LRP10 | 0.60295073 | hypo | chr14:23348800-23348801_+ | 3'UTR | 4793 | 0.03326331 |
| ZC3H12A | 0.60328406 | hypo | chr1:37948466-37948467_+ | CDS | 1142 | 0.0086185 |
| EPOR | 0.60336443 | hypo | chr19:11488657-11488658_- | 3'UTR | 1635 | 0.00343458 |
| MYO10 | 0.60385429 | hypo | chr5:16666562-16666563_- | 3'UTR | 6882 | 0.02845256 |
| ITGA5 | 0.60408346 | hypo | chr12:54789945-54789946_- | 3'UTR | 3348 | 0.00400837 |
| RND3 | 0.60411498 | hypo | chr2:151326734-151326735_- | CDS | 778 | 0.03700715 |
| DSP | 0.60415122 | hypo | chr6:7584136-7584137_+ | CDS | 6982 | 0.01232588 |
| BCAR3 | 0.60517218 | hypo | chr1:94047943-94047944_- | CDS | 1874 | 0.01172105 |
| MRPL50 | 0.60532701 | hypo | chr9:104152730-104152731_- | 3'UTR | 538 | 0.04957112 |
| NUPL2 | 0.60561212 | hypo | chr7:23240176-23240177_+ | CDS | 1122 | 0.02129383 |
| RNF20 | 0.6056855 | hypo | chr9:104324799-104324800_+ | 3'UTR | 3115 | 0.03202251 |
| GPR75 | 0.60571876 | hypo | chr2:54080687-54080688_- | CDS | 1520 | 0.03631636 |
| PPP2R5E | 0.60590416 | hypo | chr14:63842504-63842505_- | 3'UTR | 2231 | 0.03791274 |
| DCUN1D3 | 0.60591383 | hypo | chr16:20871475-20871476_- | CDS | 906 | 0.00251547 |
| ITPRIP | 0.60619374 | hypo | chr10:106073099-106073100_- | 3'UTR | 2850 | 0.04700737 |
| GUCD1 | 0.60689193 | hypo | chr22:24936424-24936425_- | 3'UTR | 3587 | 0.01116367 |
| POLR3E | 0.6069213 | hypo | chr16:22343481-22343482_+ | CDS | 2246 | 0.01582352 |
| UBL4A | 0.60699683 | hypo | chrX:153713200-153713201_- | 3'UTR | 1236 | 0.0156016 |
| YTHDF2 | 0.60709025 | hypo | chr1:29069282-29069283_+ | CDS | 763 | 0.03584028 |
| MBNL1 | 0.60725274 | hypo | chr3:152017325-152017326_+ | 5'UTR | 132 | 0.014532 |
| SETD1A | 0.60796557 | hypo | chr16:30991372-30991373_+ | CDS | 4951 | 0.03590388 |
| AP3D1 | 0.608772 | hypo | chr19:2101053-2101054_- | 3'UTR | 4997 | 0.02193299 |
| ZSCAN32 | 0.6091728 | hypo | chr16:3434466-3434467_- | CDS | 1431 | 0.02334399 |
| ZBTB26 | 0.60929115 | hypo | chr9:125681823-125681824_- | CDS | 514 | 0.04144991 |
| RPL36 | 0.60956709 | hypo | chr19:5691432-5691433_+ | CDS | 231 | 0.02549572 |
| CUX1 | 0.60983766 | hypo | chr7:101870845-101870846_+ | CDS | 3343 | 0.02704834 |
| CDYL | 0.61086174 | hypo | chr6:4892286-4892287_+ | CDS | 702 | 0.01844725 |
| NECAP2 | 0.61186069 | hypo | chr1:16785596-16785597_+ | 3'UTR | 1017 | 0.02835699 |
| RRAGA | 0.6127565 | hypo | chr9:19049856-19049857_+ | CDS | 485 | 0.02270879 |
| NINL | 0.61291911 | hypo | chr20:25434083-25434084_- | 3'UTR | 4239 | 0.02408613 |
| LMAN2L | 0.61297849 | hypo | chr2:97372815-97372816_- | 3'UTR | 1292 | 0.02027957 |
| ZBTB26 | 0.61327509 | hypo | chr9:125681763-125681764_- | CDS | 574 | 0.04959923 |
| DDOST | 0.61345288 | hypo | chr1:20981122-20981123_- | CDS | 822 | 0.04892375 |
| ZNF146 | 0.61393318 | hypo | chr19:36728332-36728333_+ | 3'UTR | 2439 | 0.03692255 |
| KDM3B | 0.61405468 | hypo | chr5:137726994-137726995_+ | CDS | 1873 | 0.02824558 |
| MUL1 | 0.61448435 | hypo | chr1:20826585-20826586_- | 3'UTR | 1812 | 0.01491383 |
| NCKAP5L | 0.61474529 | hypo | chr12:50190173-50190174_- | CDS | 1670 | 0.00188833 |
| ZNF565 | 0.61520486 | hypo | chr19:36674438-36674439_- | CDS | 545 | 0.00666743 |
| ZNF264 | 0.61546202 | hypo | chr19:57722951-57722952_+ | CDS | 900 | 0.03046224 |
| EEF2 | 0.61603322 | hypo | chr19:3976344-3976345_- | 3'UTR | 2866 | 0.04055521 |
| NMRAL1 | 0.61610995 | hypo | chr16:4513748-4513749_- | CDS | 1007 | 0.0365248 |
| PRDM4 | 0.61611565 | hypo | chr12:108128051-108128052_- | CDS | 2777 | 0.0396634 |
| BMPR2 | 0.61650165 | hypo | chr2:203420951-203420952_+ | CDS | 3711 | 0.04794083 |
| DNAJA1 | 0.61731762 | hypo | chr9:33038915-33038916_+ | 3'UTR | 1399 | 0.04733578 |
| DDX5 | 0.61735401 | hypo | chr17:62495976-62495977_- | 3'UTR | 2310 | 0.02797271 |
| TRIM21 | 0.6178205 | hypo | chr11:4406919-4406920_- | CDS | 1135 | 0.03385107 |
| NAGLU | 0.61799205 | hypo | chr17:40696188-40696189_+ | CDS | 2504 | 0.01828054 |
| POLR1B | 0.61811399 | hypo | chr2:113332869-113332870_+ | CDS | 3551 | 0.0168392 |
| SMAD2 | 0.61883878 | hypo | chr18:45422964-45422965_- | CDS | 562 | 0.04860111 |
| RAVER1 | 0.61927933 | hypo | chr19:10427232-10427233_- | 3'UTR | 3138 | 0.02340284 |
| FAM171A1 | 0.6193669 | hypo | chr10:15255541-15255542_- | CDS | 2051 | 0.0175182 |
| MAVS | 0.61947945 | hypo | chr20:3854521-3854522_+ | 3'UTR | 9522 | 0.03299994 |
| LSG1 | 0.61952389 | hypo | chr3:194362255-194362256_- | 3'UTR | 2832 | 0.02689317 |
| TSC22D1 | 0.61954285 | hypo | chr13:45008636-45008637_- | 3'UTR | 3837 | 0.01751931 |
| DAXX | 0.62050526 | hypo | chr6:33287560-33287561_- | CDS | 1673 | 0.0099848 |
| BSDC1 | 0.6207935 | hypo | chr1:32842070-32842071_- | CDS | 1045 | 0.0212938 |
| ATXN7L3 | 0.62131346 | hypo | chr17:42271640-42271641_- | CDS | 1145 | 0.003596 |
| NAPEPLD | 0.62147591 | hypo | chr7:102760244-102760245_- | CDS | 1047 | 0.01902679 |
| GFOD1 | 0.62172598 | hypo | chr6:13365104-13365105_- | CDS | 1789 | 0.01011067 |
| UBC | 0.6218988 | hypo | chr12:125397571-125397572_- | CDS | 1203 | 0.03329349 |
| IRS4 | 0.62202149 | hypo | chrX:107978716-107978717_- | CDS | 890 | 0.00159733 |
| KIF3C | 0.62282094 | hypo | chr2:26151296-26151297_- | 3'UTR | 3588 | 0.04278354 |
| CHAMP1 | 0.62297269 | hypo | chr13:115091181-115091182_+ | CDS | 2348 | 0.01126399 |
| EEF2 | 0.62309713 | hypo | chr19:3976160-3976161_- | 3'UTR | 3050 | 0.0316489 |
| ANKFY1 | 0.62337853 | hypo | chr17:4067924-4067925_- | 3'UTR | 6900 | 0.04607251 |
| HES1 | 0.62364385 | hypo | chr3:193856085-193856086_+ | 3'UTR | 1139 | 0.0369207 |
| ZNF395 | 0.62383011 | hypo | chr8:28206130-28206131_- | 3'UTR | 1772 | 0.04873634 |
| C12orf43 | 0.6242307 | hypo | chr12:121441892-121441893_- | 3'UTR | 879 | 0.01559934 |
| ZMIZ2 | 0.62436907 | hypo | chr7:44807302-44807303_+ | 3'UTR | 2966 | 0.04419585 |
| ARSJ | 0.62438377 | hypo | chr4:114824190-114824191_- | CDS | 1926 | 0.00680383 |
| USP15 | 0.6244917 | hypo | chr12:62798181-62798182_+ | 3'UTR | 3047 | 0.02450716 |
| NIF3L1 | 0.62492384 | hypo | chr2:201768375-201768376_+ | CDS | 1199 | 0.01646927 |
| HDLBP | 0.62503311 | hypo | chr2:242169002-242169003_- | 3'UTR | 4047 | 0.0094322 |
| TBL1X | 0.62526725 | hypo | chrX:9684588-9684589_+ | 3'UTR | 2523 | 0.04896677 |
| COQ9 | 0.62534552 | hypo | chr16:57494579-57494580_+ | 3'UTR | 1027 | 0.02255966 |
| KLHL42 | 0.62547206 | hypo | chr12:27950821-27950822_+ | CDS | 1317 | 0.03024063 |
| CELSR3 | 0.62555325 | hypo | chr3:48696714-48696715_- | CDS | 3633 | 0.04227363 |
| ERGIC3 | 0.6258282 | hypo | chr20:34144775-34144776_+ | CDS | 995 | 0.04060452 |
| ALPI | 0.6268416 | hypo | chr2:233322725-233322726_+ | CDS | 962 | 0.02385529 |
| UXT | 0.62705773 | hypo | chrX:47511219-47511220_- | 3'UTR | 747 | 0.03184327 |
| SLC35E1 | 0.62709138 | hypo | chr19:16661842-16661843_- | 3'UTR | 3930 | 0.03392378 |
| ZNF532 | 0.62717595 | hypo | chr18:56651579-56651580_+ | CDS | 4563 | 0.01541158 |
| SDR39U1 | 0.62723285 | hypo | chr14:24909105-24909106_- | 3'UTR | 1096 | 0.04134136 |
| CYP2S1 | 0.62726165 | hypo | chr19:41713121-41713122_+ | 3'UTR | 2301 | 0.01517447 |
| DDX24 | 0.62753984 | hypo | chr14:94528629-94528630_- | CDS | 1154 | 0.03177671 |
| RCN1 | 0.62819545 | hypo | chr11:32126970-32126971_+ | 3'UTR | 2214 | 0.0394136 |
| SYNRG | 0.6282008 | hypo | chr17:35878516-35878517_- | 3'UTR | 4319 | 0.01661004 |
| SLC12A6 | 0.62824153 | hypo | chr15:34629585-34629586_- | 5'UTR | 375 | 0.03645849 |
| KMT2C | 0.62852279 | hypo | chr7:151878887-151878888_- | CDS | 6273 | 0.03576598 |
| ASS1 | 0.62863777 | hypo | chr9:133327692-133327693_+ | CDS | 433 | 0.03325977 |
| JOSD1 | 0.62870194 | hypo | chr22:39083811-39083812_- | 3'UTR | 1398 | 0.03880105 |
| GRAMD4 | 0.62874733 | hypo | chr22:47073477-47073478_+ | 3'UTR | 2295 | 0.00457017 |
| SEC61A1 | 0.62884748 | hypo | chr3:127789069-127789070_+ | 3'UTR | 2179 | 0.01964176 |
| CCDC47 | 0.62899808 | hypo | chr17:61824142-61824143_- | 3'UTR | 1885 | 0.03592804 |
| TANC1 | 0.6291154 | hypo | chr2:160088378-160088379_+ | 3'UTR | 6715 | 0.04435298 |
| PIGV | 0.62933193 | hypo | chr1:27121554-27121555_+ | CDS | 1392 | 0.01354111 |
| ZFP1 | 0.62966675 | hypo | chr16:75203833-75203834_+ | CDS | 1071 | 0.01992731 |
| CBFA2T2 | 0.6308633 | hypo | chr20:32232922-32232923_+ | 3'UTR | 2404 | 0.02747822 |
| EEF2 | 0.63113506 | hypo | chr19:3977853-3977854_- | CDS | 2112 | 0.03540289 |
| ZNF584 | 0.63116927 | hypo | chr19:58929440-58929441_+ | 3'UTR | 2029 | 0.01696084 |
| KIAA0930 | 0.63157849 | hypo | chr22:45592518-45592519_- | 3'UTR | 1890 | 0.0385966 |
| SNAP47 | 0.63161479 | hypo | chr1:227968701-227968702_+ | 3'UTR | 2136 | 0.04500178 |
| IQSEC1 | 0.63175754 | hypo | chr3:12940479-12940480_- | 3'UTR | 5761 | 0.01097773 |
| MTPAP | 0.63183632 | hypo | chr10:30602330-30602331_- | 3'UTR | 2018 | 0.03471093 |
| INVS | 0.63211949 | hypo | chr9:103055128-103055129_+ | CDS | 2787 | 0.04491018 |
| MEX3D | 0.63269943 | hypo | chr19:1556782-1556783_- | CDS | 734 | 0.00370184 |
| MAP3K7 | 0.63282996 | hypo | chr6:91225879-91225880_- | 3'UTR | 2349 | 0.04546677 |
| CWC22 | 0.63347133 | hypo | chr2:180810284-180810285_- | CDS | 2597 | 0.04293867 |
| TSPYL1 | 0.63383859 | hypo | chr6:116600054-116600055_- | CDS | 1225 | 0.00804796 |
| RBBP6 | 0.63409544 | hypo | chr16:24582751-24582752_+ | CDS | 5404 | 0.03270801 |
| CETN2 | 0.63410814 | hypo | chrX:151995982-151995983_- | 3'UTR | 967 | 0.04639167 |
| RBM12 | 0.63415764 | hypo | chr20:34242025-34242026_- | CDS | 1480 | 0.04251841 |
| SPEN | 0.63447732 | hypo | chr1:16255898-16255899_+ | CDS | 3367 | 0.03198295 |
| PAFAH1B2 | 0.6346585 | hypo | chr11:117038316-117038317_+ | CDS | 733 | 0.03584912 |
| RNASEH1 | 0.63475438 | hypo | chr2:3593389-3593390_- | CDS | 956 | 0.02667275 |
| EEF2 | 0.63480788 | hypo | chr19:3977588-3977589_- | CDS | 2169 | 0.04896363 |
| ZNF266 | 0.63484817 | hypo | chr19:9523752-9523753_- | 3'UTR | 3087 | 0.00251136 |
| EXT2 | 0.63505059 | hypo | chr11:44129566-44129567_+ | CDS | 459 | 0.02453155 |
| AP2A2 | 0.63520777 | hypo | chr11:1010785-1010786_+ | 3'UTR | 3196 | 0.02374016 |
| EEF2 | 0.63521189 | hypo | chr19:3980007-3980008_- | CDS | 1485 | 0.01701312 |
| DHTKD1 | 0.63549558 | hypo | chr10:12163288-12163289_+ | 3'UTR | 3278 | 0.02420853 |
| HSP90AB1 | 0.63613313 | hypo | chr6:44219196-44219197_+ | CDS | 1344 | 0.04440976 |
| NRIP1 | 0.6370977 | hypo | chr21:16339107-16339108_- | CDS | 2003 | 0.04303367 |
| USP4 | 0.6374136 | hypo | chr3:49315790-49315791_- | CDS | 2905 | 0.03846308 |
| FOXO1 | 0.63743137 | hypo | chr13:41134395-41134396_- | CDS | 1660 | 0.01579772 |
| CLTB | 0.63759475 | hypo | chr5:175824696-175824697_- | CDS | 580 | 0.03346733 |
| SIRPA | 0.63776999 | hypo | chr20:1919468-1919469_+ | 3'UTR | 2796 | 0.0235013 |
| ELOA | 0.63804754 | hypo | chr1:24078002-24078003_+ | CDS | 1045 | 0.03571942 |
| WHAMM | 0.63812496 | hypo | chr15:83503107-83503108_+ | 3'UTR | 3348 | 0.03959858 |
| COL5A1 | 0.63901627 | hypo | chr9:137734320-137734321_+ | 3'UTR | 6071 | 0.04444894 |
| TCF3 | 0.63910363 | hypo | chr19:1611280-1611281_- | 3'UTR | 2085 | 0.03242847 |
| MNX1 | 0.63918085 | hypo | chr7:156798361-156798362_- | CDS | 1360 | 0.00320027 |
| ARHGDIA | 0.63944616 | hypo | chr17:79825906-79825907_- | 3'UTR | 1526 | 0.03352002 |
| SLAMF7 | 0.63948162 | hypo | chr1:160724339-160724340_+ | 3'UTR | 2625 | 0.0305377 |
| TRMT61B | 0.63981192 | hypo | chr2:29092916-29092917_- | CDS | 258 | 0.01891536 |
| TXNDC12 | 0.63992001 | hypo | chr1:52486332-52486333_- | 3'UTR | 1865 | 0.03440774 |
| POP5 | 0.64002759 | hypo | chr12:121016996-121016997_- | 3'UTR | 647 | 0.03098625 |
| PWWP3A | 0.64039031 | hypo | chr19:1377873-1377874_+ | 3'UTR | 3562 | 0.0446714 |
| PPP1R35 | 0.64056863 | hypo | chr7:100033025-100033026_- | CDS | 817 | 0.03179285 |
| VPS9D1-AS1 | 0.64069815 | hypo | chr16:89784082-89784083_+ | 1262 | 1262 | 0.01585443 |
| QSOX1 | 0.64069954 | hypo | chr1:180166632-180166633_+ | 3'UTR | 2779 | 0.02479644 |
| PCMTD1 | 0.64072412 | hypo | chr8:52773532-52773533_- | CDS | 380 | 0.04938487 |
| TNFAIP3 | 0.64081117 | hypo | chr6:138200172-138200173_+ | CDS | 1936 | 0.01547473 |
| PHF12 | 0.64126011 | hypo | chr17:27240032-27240033_- | CDS | 2085 | 0.0359161 |
| RNF40 | 0.64147964 | hypo | chr16:30785958-30785959_+ | 3'UTR | 4349 | 0.03084235 |
| EWSR1 | 0.64231486 | hypo | chr22:29693876-29693877_+ | CDS | 1697 | 0.02513448 |
| CTDNEP1 | 0.64338854 | hypo | chr17:7147338-7147339_- | 3'UTR | 1279 | 0.03838375 |
| KMT2C | 0.64354667 | hypo | chr7:151874851-151874852_- | CDS | 7902 | 0.03415906 |
| ZBTB7B | 0.64374971 | hypo | chr1:154989015-154989016_+ | CDS | 2034 | 0.03924186 |
| AHDC1 | 0.64376146 | hypo | chr1:27878009-27878010_- | CDS | 1447 | 0.04061354 |
| IGF1R | 0.64429494 | hypo | chr15:99505655-99505656_+ | 3'UTR | 10131 | 0.0436739 |
| PLEKHM2 | 0.64476771 | hypo | chr1:16060754-16060755_+ | 3'UTR | 3641 | 0.0411734 |
| GIMAP1 | 0.64485516 | hypo | chr7:150418012-150418013_+ | CDS | 1060 | 0.0466833 |
| SNX21 | 0.64531399 | hypo | chr20:44469845-44469846_+ | CDS | 1104 | 0.04210598 |
| ZBED3 | 0.64542547 | hypo | chr5:76372971-76372972_- | 3'UTR | 1019 | 0.01889593 |
| TMEM51 | 0.64543019 | hypo | chr1:15546710-15546711_+ | 3'UTR | 1648 | 0.03789551 |
| RIC8B | 0.64592822 | hypo | chr12:107209059-107209060_+ | CDS | 908 | 0.01135809 |
| MVB12B | 0.64609885 | hypo | chr9:129266127-129266128_+ | 3'UTR | 1631 | 0.00607223 |
| STC2 | 0.64635893 | hypo | chr5:172745029-172745030_- | CDS | 2038 | 0.0420258 |
| SALL1 | 0.64714534 | hypo | chr16:51175107-51175108_- | CDS | 1055 | 0.02596873 |
| YIF1A | 0.64715223 | hypo | chr11:66055337-66055338_- | CDS | 450 | 0.04801003 |
| NIN | 0.64728418 | hypo | chr14:51224822-51224823_- | CDS | 3115 | 0.01609346 |
| FBXO31 | 0.64752002 | hypo | chr16:87364102-87364103_- | 3'UTR | 2459 | 0.03982548 |
| MAP3K4 | 0.64844577 | hypo | chr6:161470895-161470896_+ | CDS | 1796 | 0.03275065 |
| ABR | 0.64852743 | hypo | chr17:907358-907359_- | 3'UTR | 4787 | 0.01933855 |
| SMIM26 | 0.64962458 | hypo | chr20:18548221-18548222_+ | CDS | 158 | 0.04648217 |
| PRRC2C | 0.64965638 | hypo | chr1:171560848-171560849_+ | CDS | 8582 | 0.02158408 |
| ZFYVE9 | 0.65007876 | hypo | chr1:52703361-52703362_+ | CDS | 724 | 0.04511566 |
| CCDC57 | 0.65026536 | hypo | chr17:80114690-80114691_- | 3'UTR | 3593 | 0.01590301 |
| TENT4B | 0.65056469 | hypo | chr16:50263199-50263200_+ | CDS | 2092 | 0.03492322 |
| NAGLU | 0.6512093 | hypo | chr17:40695945-40695946_+ | CDS | 2261 | 0.0147778 |
| SLC4A2 | 0.65146675 | hypo | chr7:150767993-150767994_+ | CDS | 1981 | 0.01150968 |
| DCP1A | 0.65164073 | hypo | chr3:53321554-53321555_- | 3'UTR | 1901 | 0.00259049 |
| BRD4 | 0.65215617 | hypo | chr19:15367024-15367025_- | CDS | 1822 | 0.00238412 |
| WSCD1 | 0.65278596 | hypo | chr17:6024479-6024480_+ | 3'UTR | 2553 | 0.0482913 |
| PSEN1 | 0.65321394 | hypo | chr14:73686195-73686196_+ | 3'UTR | 1814 | 0.04480981 |
| TRPS1 | 0.65328211 | hypo | chr8:116599418-116599419_- | CDS | 3113 | 0.01354777 |
| SLC12A7 | 0.65368228 | hypo | chr5:1051783-1051784_- | 3'UTR | 4008 | 0.03858766 |
| TUT1 | 0.6541044 | hypo | chr11:62343221-62343222_- | CDS | 2120 | 0.00910725 |
| OLFM1 | 0.6542245 | hypo | chr9:138011746-138011747_+ | CDS | 1590 | 0.00323154 |
| LRRC8C | 0.65437949 | hypo | chr1:90180532-90180533_+ | CDS | 2645 | 0.03633878 |
| BAP1 | 0.65446357 | hypo | chr3:52435981-52435982_- | 3'UTR | 2641 | 0.03709956 |
| ATG9A | 0.65482351 | hypo | chr2:220088972-220088973_- | CDS | 1302 | 0.03328263 |
| MAT2A | 0.65488333 | hypo | chr2:85771693-85771694_+ | 3'UTR | 2296 | 0.03832928 |
| TIGAR | 0.65506521 | hypo | chr12:4461952-4461953_+ | 3'UTR | 987 | 0.04133341 |
| NUDT19 | 0.65511479 | hypo | chr19:33202817-33202818_+ | CDS | 1082 | 0.0130575 |
| G3BP1 | 0.65516573 | hypo | chr5:151166263-151166264_+ | CDS | 222 | 0.02589643 |
| MAML1 | 0.65577652 | hypo | chr5:179200998-179200999_+ | CDS | 2434 | 0.02027746 |
| CCDC88A | 0.65603325 | hypo | chr2:55523112-55523113_- | CDS | 6009 | 0.01142936 |
| YLPM1 | 0.65660374 | hypo | chr14:75248259-75248260_+ | CDS | 1637 | 0.04964947 |
| INIP | 0.65680274 | hypo | chr9:115449811-115449812_- | 3'UTR | 539 | 0.01522377 |
| ZNF274 | 0.65685128 | hypo | chr19:58724620-58724621_+ | 3'UTR | 2270 | 0.00373067 |
| TRAM2 | 0.65757397 | hypo | chr6:52362541-52362542_- | 3'UTR | 6714 | 0.02323842 |
| WDFY2 | 0.65784601 | hypo | chr13:52333988-52333989_+ | 3'UTR | 1626 | 0.03487133 |
| EDF1 | 0.65803495 | hypo | chr9:139756734-139756735_- | CDS | 497 | 0.02808517 |
| ZNF189 | 0.65860046 | hypo | chr9:104171594-104171595_+ | CDS | 1847 | 0.03729096 |
| RTL10 | 0.65862601 | hypo | chr22:19839623-19839624_- | CDS | 671 | 0.01816641 |
| RPIA | 0.65898744 | hypo | chr2:89049569-89049570_+ | CDS | 951 | 0.04466979 |
| LAS1L | 0.65901833 | hypo | chrX:64737982-64737983_- | CDS | 1901 | 0.02472422 |
| ZC3HAV1 | 0.65957754 | hypo | chr7:138764509-138764510_- | CDS | 1564 | 0.03469701 |
| NCOA7 | 0.65972872 | hypo | chr6:126210613-126210614_+ | CDS | 1782 | 0.01189028 |
| PEX10 | 0.66024758 | hypo | chr1:2336733-2336734_- | 3'UTR | 1580 | 0.04759718 |
| DUSP1 | 0.66064357 | hypo | chr5:172195816-172195817_- | CDS | 1294 | 0.00245804 |
| PALB2 | 0.66071942 | hypo | chr16:23647077-23647078_- | CDS | 988 | 0.01323882 |
| PRSS23 | 0.66088341 | hypo | chr11:86662557-86662558_+ | 878 | 878 | 0.03523416 |
| DYRK1A | 0.66131732 | hypo | chr21:38884620-38884621_+ | CDS | 2416 | 0.03251987 |
| SASH1 | 0.66163389 | hypo | chr6:148869719-148869720_+ | 3'UTR | 4244 | 0.03561803 |
| WDR33 | 0.66171955 | hypo | chr2:128463824-128463825_- | 3'UTR | 4281 | 0.01913515 |
| TMEM115 | 0.6618406 | hypo | chr3:50392592-50392593_- | 3'UTR | 1681 | 0.0497052 |
| MRM3 | 0.66200133 | hypo | chr17:695205-695206_+ | CDS | 1185 | 0.0225092 |
| XBP1 | 0.66203041 | hypo | chr22:29191244-29191245_- | CDS | 1096 | 0.04226302 |
| BTN3A1 | 0.66204444 | hypo | chr6:26413210-26413211_+ | 3'UTR | 1957 | 0.03091642 |
| MARCH2 | 0.66210319 | hypo | chr19:8503564-8503565_+ | 3'UTR | 1045 | 0.00787639 |
| PPIL2 | 0.66296373 | hypo | chr22:22052044-22052045_+ | 3'UTR | 2552 | 0.03304056 |
| DEDD2 | 0.66309602 | hypo | chr19:42702953-42702954_- | 3'UTR | 1733 | 0.03685059 |
| COQ6 | 0.66311465 | hypo | chr14:74429742-74429743_+ | 3'UTR | 1528 | 0.00983917 |
| NRG1 | 0.66329092 | hypo | chr8:32406155-32406156_+ | 5'UTR | 428 | 0.04635112 |
| SFT2D3 | 0.66336417 | hypo | chr2:128460245-128460246_+ | 3'UTR | 1649 | 0.03856591 |
| RBBP8 | 0.66338288 | hypo | chr18:20573330-20573331_+ | CDS | 1871 | 0.03985883 |
| SERPINH1 | 0.66411453 | hypo | chr11:75283177-75283178_+ | 3'UTR | 1604 | 0.04382989 |
| GCC1 | 0.66428457 | hypo | chr7:127222221-127222222_- | CDS | 2591 | 0.02333253 |
| NCBP2-AS2 | 0.66437327 | hypo | chr3:196670132-196670133_+ | 3'UTR | 639 | 0.04281445 |
| METTL14 | 0.66448215 | hypo | chr4:119631466-119631467_+ | 3'UTR | 1545 | 0.03820514 |
| ZC3H4 | 0.66519207 | hypo | chr19:47569740-47569741_- | CDS | 3821 | 0.00142837 |
| SLC1A5 | 0.66567723 | hypo | chr19:47278826-47278827_- | CDS | 2194 | 0.00872535 |
| VPS39 | 0.66593811 | hypo | chr15:42452812-42452813_- | 3'UTR | 2939 | 0.03401012 |
| RARRES3 | 0.66595708 | hypo | chr11:63313890-63313891_+ | 3'UTR | 725 | 0.04818899 |
| SNIP1 | 0.66615556 | hypo | chr1:38006180-38006181_- | CDS | 617 | 0.03990341 |
| RACK1 | 0.66654517 | hypo | chr5:180668573-180668574_- | CDS | 452 | 0.03574374 |
