# Supplementary material for: Specific RNA m6A modification sites in bone marrow mesenchymal stem cells from the jawbone marrow of type 2 diabetes patients with dental implant failure
Source: Int J Oral Sci. 2023 Jan 12;15:6. doi: 10.1038/s41368-022-00202-3 (PMC9834262; doi:10.1038/s41368-022-00202-3)
Supplement: Supplementary file 5 — Supplementary Table S5 [file 41368_2022_202_MOESM5_ESM.docx]

**Table S5. PPI network of differentially m6A-methylated genes in DM-BMSCs.**

| **Gene symbol** | **Degree** | **Neighborhood Connectivity** | **Betweenness Centrality** | **Closeness Centrality** |
| --- | --- | --- | --- | --- |
| HSP90AA1 | 53 | 16.03774 | 0.159243 | 0.402281 |
| UBC | 51 | 12.66667 | 0.15241 | 0.376781 |
| ACTB | 49 | 15.34694 | 0.119328 | 0.384168 |
| RPL3 | 36 | 21.52778 | 0.023101 | 0.330006 |
| RPS15 | 36 | 20.16667 | 0.014713 | 0.315632 |
| CTNNB1 | 35 | 15 | 0.07019 | 0.362577 |
| HSP90AB1 | 35 | 17.65714 | 0.035365 | 0.363574 |
| RPLP0 | 34 | 23.11765 | 0.021681 | 0.340193 |
| RPS4X | 34 | 22.08824 | 0.020468 | 0.331661 |
| RPL19 | 33 | 21.84848 | 0.036702 | 0.34485 |
| NHP2L1 | 32 | 19.59375 | 0.025663 | 0.313018 |
| RPS6 | 32 | 20.9375 | 0.006292 | 0.316956 |
| EFTUD2 | 32 | 16.09375 | 0.057758 | 0.33187 |
| MYC | 31 | 14.77419 | 0.066416 | 0.356229 |
| RPS24 | 31 | 20.6129 | 0.008353 | 0.313947 |
| RAC1 | 30 | 13.5 | 0.062288 | 0.342173 |
| RPL35A | 30 | 21.16667 | 0.017486 | 0.310082 |
| MRPS7 | 29 | 21.55172 | 0.010367 | 0.294543 |
| GAPDH | 28 | 19.85714 | 0.068969 | 0.366343 |
| RPL37A | 28 | 21.67857 | 0.003378 | 0.305075 |
| VCP | 27 | 13.14815 | 0.047715 | 0.344625 |
| EEF2 | 27 | 25.07407 | 0.009196 | 0.328571 |
| MRPS14 | 24 | 22.16667 | 0.008839 | 0.298028 |
| PIK3R1 | 23 | 12.21739 | 0.025422 | 0.319252 |
| RHOA | 23 | 16.6087 | 0.050625 | 0.351963 |
| NCBP2 | 23 | 8.73913 | 0.029973 | 0.28797 |
| SMAD2 | 22 | 13.72727 | 0.040511 | 0.336943 |
| CAV1 | 22 | 18.63636 | 0.033055 | 0.344401 |
| SEC61A1 | 22 | 20.77273 | 0.028416 | 0.305075 |
| PA2G4 | 22 | 23.68182 | 0.001326 | 0.294216 |
| JUN | 21 | 16.90476 | 0.038241 | 0.342173 |
| CCT4 | 21 | 25.42857 | 0.007138 | 0.328367 |
| ETF1 | 21 | 23.2381 | 0.003557 | 0.29887 |
| GTPBP4 | 21 | 18.09524 | 0.018085 | 0.308455 |
| CCT5 | 20 | 23.7 | 0.011618 | 0.326947 |
| RBM25 | 20 | 13.5 | 0.019329 | 0.290659 |
| APP | 18 | 15.33333 | 0.039188 | 0.333965 |
| POLR1C | 18 | 20.77778 | 0.015578 | 0.318867 |
| NIP7 | 18 | 19.38889 | 0.015954 | 0.289229 |
| SNW1 | 18 | 14.38889 | 0.018867 | 0.308096 |
| MRPL36 | 18 | 20.16667 | 0.005791 | 0.285483 |
| GSPT1 | 17 | 24.47059 | 0.005478 | 0.300057 |
| EIF5B | 17 | 24.64706 | 0.003114 | 0.2905 |
| PKM | 16 | 14.5 | 0.038783 | 0.329595 |
| DDB1 | 16 | 14.3125 | 0.01777 | 0.320412 |
| MAPT | 16 | 16.75 | 0.021069 | 0.326745 |
| POLR1B | 16 | 16.625 | 0.012525 | 0.310264 |
| SMARCA4 | 16 | 15.0625 | 0.019784 | 0.3208 |
| BPTF | 16 | 7.625 | 0.013362 | 0.281084 |
| EIF4G1 | 16 | 18.125 | 0.021439 | 0.308275 |
| RPP38 | 16 | 21.125 | 0.011099 | 0.294872 |
| PHF5A | 16 | 13.4375 | 0.007708 | 0.275952 |
| UTP15 | 16 | 20.25 | 0.002813 | 0.29275 |
| TCP1 | 15 | 25.73333 | 0.001967 | 0.318867 |
| KAT5 | 15 | 17.8 | 0.014774 | 0.324939 |
| DYNC1H1 | 15 | 15.86667 | 0.017061 | 0.313018 |
| CALR | 15 | 15.06667 | 0.025137 | 0.321972 |
| PSMA5 | 15 | 20.73333 | 0.004901 | 0.32335 |
| PPP1CB | 15 | 8.266667 | 0.02977 | 0.288755 |
| ABL1 | 14 | 17.57143 | 0.009333 | 0.3181 |
| MAP3K7 | 14 | 14.07143 | 0.007755 | 0.304198 |
| CDK9 | 14 | 14.21429 | 0.013091 | 0.320218 |
| DNAJB1 | 14 | 13.85714 | 0.001944 | 0.298533 |
| HSPA2 | 14 | 13 | 0.013715 | 0.301253 |
| NCL | 14 | 23.21429 | 0.012449 | 0.339756 |
| MRPL19 | 14 | 19.14286 | 0.001465 | 0.266499 |
| PLEC | 14 | 28.57143 | 0.002099 | 0.285637 |
| LYN | 13 | 16.07692 | 0.013353 | 0.319444 |
| SOD1 | 13 | 13.07692 | 0.027138 | 0.310994 |
| COPS2 | 13 | 19.15385 | 0.022455 | 0.319444 |
| TRRAP | 13 | 18.84615 | 0.009594 | 0.318675 |
| HSP90B1 | 13 | 16.46154 | 0.012669 | 0.309719 |
| TRAF2 | 13 | 16 | 0.006627 | 0.310994 |
| SNIP1 | 13 | 15.69231 | 0.012581 | 0.290181 |
| RIPK1 | 12 | 21.58333 | 0.003894 | 0.319637 |
| FOXO1 | 12 | 13.5 | 0.005846 | 0.306134 |
| PSMA2 | 12 | 17.16667 | 0.001835 | 0.303326 |
| DDX23 | 12 | 14.08333 | 0.002285 | 0.2717 |
| MRPL46 | 12 | 16.66667 | 1.21E-04 | 0.241995 |
| DAP3 | 12 | 16.66667 | 1.21E-04 | 0.241995 |
| MRPL41 | 12 | 16.66667 | 1.21E-04 | 0.241995 |
| CPSF2 | 12 | 7.583333 | 0.00791 | 0.249646 |
| DHX16 | 12 | 15.83333 | 1.88E-04 | 0.2724 |
| PELO | 12 | 28.41667 | 1.38E-04 | 0.283039 |
| UTP3 | 12 | 22.41667 | 0.002419 | 0.292104 |
| BCAR1 | 11 | 12.36364 | 0.013297 | 0.283342 |
| ACO2 | 11 | 7.545455 | 0.014079 | 0.269348 |
| CUL4A | 11 | 17.45455 | 0.007087 | 0.312648 |
| KMT2A | 11 | 12 | 0.010774 | 0.294543 |
| RAB5A | 11 | 11.36364 | 0.028799 | 0.292589 |
| KHDRBS1 | 11 | 10.36364 | 0.009752 | 0.293726 |
| CHCHD1 | 11 | 15 | 0 | 0.232119 |
| MRPL44 | 11 | 15 | 0 | 0.232119 |
| MRPL50 | 11 | 15 | 0 | 0.232119 |
| MRPS21 | 11 | 15 | 0 | 0.232119 |
| MRPS33 | 11 | 15 | 0 | 0.232119 |
| DDX5 | 11 | 13.72727 | 0.013253 | 0.307201 |
| PRPF4 | 11 | 15.72727 | 1.04E-04 | 0.265429 |
| MFAP1 | 11 | 16.63636 | 1.87E-04 | 0.273668 |
| SERBP1 | 11 | 31.18182 | 0 | 0.276529 |
| PSMD7 | 11 | 14 | 0.003927 | 0.292913 |
| ATG5 | 10 | 14.6 | 0.013237 | 0.302632 |
| DCTN2 | 10 | 19.3 | 0.003261 | 0.304373 |
| MLLT3 | 10 | 13.7 | 0.010106 | 0.308275 |
| AHSA1 | 10 | 16.6 | 1.55E-04 | 0.293889 |
| CLPB | 10 | 17 | 0.0017 | 0.298533 |
| CDC37 | 10 | 20.4 | 5.84E-04 | 0.300057 |
| CSNK1D | 10 | 20.6 | 0.012423 | 0.311176 |
| DYNLL1 | 10 | 14 | 0.013414 | 0.299208 |
| IRS1 | 10 | 16.4 | 0.002616 | 0.302459 |
| TNFRSF1A | 10 | 13.6 | 0.002414 | 0.2913 |
| SETD2 | 10 | 9.3 | 0.007602 | 0.271561 |
| SSRP1 | 10 | 14.2 | 0.014178 | 0.306312 |
| RANBP2 | 10 | 7.2 | 0.00449 | 0.266097 |
| CHML | 10 | 5 | 0.017379 | 0.25868 |
| ZMAT2 | 10 | 16.9 | 1.92E-05 | 0.265296 |
| PRPF38A | 10 | 14.9 | 0.001813 | 0.263315 |
| EIF3A | 10 | 23.4 | 3.41E-04 | 0.273951 |
| TWISTNB | 10 | 12.8 | 0.024032 | 0.26384 |
| POLR1D | 10 | 14.8 | 0.001116 | 0.267172 |
| USP14 | 10 | 15.2 | 0.002384 | 0.2913 |
| CRKL | 9 | 14 | 8.85E-04 | 0.281683 |
| TOMM7 | 9 | 9.555556 | 0.012213 | 0.251905 |
| CAPZA1 | 9 | 20.88889 | 4.51E-04 | 0.302632 |
| COPS6 | 9 | 18 | 0.002992 | 0.300739 |
| MYL12B | 9 | 15.66667 | 0.008635 | 0.299547 |
| SUPT4H1 | 9 | 10.55556 | 0.002959 | 0.276963 |
| EDF1 | 9 | 29.55556 | 0.002769 | 0.308455 |
| DNAJA1 | 9 | 17.88889 | 0.001329 | 0.295861 |
| MAVS | 9 | 12.44444 | 0.004717 | 0.29034 |
| BAG3 | 9 | 20.44444 | 0.002537 | 0.295696 |
| SETD1A | 9 | 12.33333 | 0.005728 | 0.288441 |
| TUBA1B | 9 | 12.33333 | 0.003385 | 0.270865 |
| TCEB3 | 9 | 25.55556 | 0.002025 | 0.286721 |
| RAB8A | 9 | 4.555556 | 0.004659 | 0.246047 |
| LTN1 | 9 | 24.11111 | 0.010172 | 0.291139 |
| GRPEL1 | 9 | 13.88889 | 0.012438 | 0.295201 |
| NUP50 | 9 | 9.222222 | 0.002247 | 0.264368 |
| LSM10 | 9 | 15.55556 | 0.003502 | 0.272259 |
| TP53BP1 | 9 | 12.22222 | 0.004782 | 0.295366 |
| EIF3L | 9 | 25.88889 | 8.62E-04 | 0.275234 |
| LSG1 | 9 | 25.55556 | 0.001303 | 0.281233 |
| VIM | 9 | 15.55556 | 0.010059 | 0.311176 |
| SDHB | 8 | 9.375 | 0.024747 | 0.274235 |
| ACTR8 | 8 | 16.5 | 2.80E-04 | 0.286255 |
| BDNF | 8 | 24.375 | 0.003192 | 0.309176 |
| MYL6 | 8 | 17.375 | 0.002742 | 0.297191 |
| YKT6 | 8 | 4.375 | 0.022103 | 0.231611 |
| NDUFS5 | 8 | 4.75 | 0.007139 | 0.226359 |
| BID | 8 | 9.125 | 0.00483 | 0.275091 |
| USP34 | 8 | 12.875 | 0.013551 | 0.288283 |
| NIPBL | 8 | 6.125 | 0.007827 | 0.251306 |
| TRIP12 | 8 | 7 | 0.005193 | 0.249882 |
| SYNCRIP | 8 | 12.875 | 0.004938 | 0.300227 |
| MANF | 8 | 7.375 | 0.022976 | 0.266231 |
| TIMM8A | 8 | 6.75 | 0.003015 | 0.238611 |
| NCOA1 | 8 | 9 | 0.01604 | 0.277108 |
| PCF11 | 8 | 9.375 | 0.003006 | 0.23915 |
| SRSF4 | 8 | 11.5 | 0.001127 | 0.242772 |
| SRSF5 | 8 | 10.5 | 0.008324 | 0.242883 |
| HNRNPU | 8 | 9.875 | 0.003145 | 0.279303 |
| SPOP | 8 | 12.875 | 0.030356 | 0.296691 |
| PSME4 | 8 | 15.25 | 0.001082 | 0.281084 |
| PSMB5 | 8 | 15.875 | 7.40E-04 | 0.28797 |
| TOMM5 | 7 | 6.857143 | 6.32E-04 | 0.225011 |
| TIMM22 | 7 | 7 | 4.32E-04 | 0.224915 |
| ARPC3 | 7 | 14.71429 | 5.87E-04 | 0.29034 |
| MCL1 | 7 | 28.71429 | 0.002115 | 0.319444 |
| ANXA2 | 7 | 12 | 0.008053 | 0.287032 |
| DNAJB4 | 7 | 20.71429 | 3.29E-05 | 0.29275 |
| GNA12 | 7 | 21.57143 | 0.001901 | 0.310994 |
| PSEN1 | 7 | 12.14286 | 0.008056 | 0.285637 |
| ARFGAP1 | 7 | 11.14286 | 0.024856 | 0.274235 |
| APC | 7 | 18 | 0.003216 | 0.305251 |
| CLIP1 | 7 | 11.28571 | 0.00477 | 0.275808 |
| CAPZA2 | 7 | 19.28571 | 2.01E-04 | 0.2913 |
| RBPJ | 7 | 15.57143 | 0.003985 | 0.293563 |
| HIST1H3B | 7 | 8.857143 | 0.005462 | 0.27184 |
| XRCC5 | 7 | 7.571429 | 0.007673 | 0.257797 |
| COA6 | 7 | 5.142857 | 0.008162 | 0.218957 |
| CUL2 | 7 | 17.42857 | 0.00245 | 0.291943 |
| PAPOLA | 7 | 11.28571 | 0.002982 | 0.248008 |
| NUP133 | 7 | 10.85714 | 0.001397 | 0.261235 |
| ZEB1 | 7 | 18.28571 | 0.002862 | 0.296027 |
| POLR3B | 7 | 13.57143 | 4.32E-04 | 0.259187 |
| MATR3 | 7 | 11.14286 | 0.003232 | 0.285483 |
| NPLOC4 | 7 | 14.71429 | 0.001868 | 0.289229 |
| SHFM1 | 7 | 16.42857 | 0 | 0.279598 |
| YWHAH | 6 | 14 | 0.002608 | 0.285022 |
| ACLY | 6 | 8.166667 | 0.002547 | 0.256672 |
| PDHB | 6 | 8.5 | 2.21E-04 | 0.254327 |
| ITGA5 | 6 | 17.16667 | 0.003821 | 0.292427 |
| DYNLRB1 | 6 | 19.16667 | 8.71E-04 | 0.293075 |
| PIP5K1C | 6 | 27 | 0.001459 | 0.301768 |
| CETN2 | 6 | 16.83333 | 0.002857 | 0.288441 |
| AFF4 | 6 | 11.5 | 6.58E-04 | 0.265163 |
| UNC45A | 6 | 19.5 | 0.001065 | 0.288598 |
| CLTB | 6 | 4.833333 | 0.002354 | 0.237113 |
| RB1CC1 | 6 | 5.5 | 0.003561 | 0.251545 |
| DDX6 | 6 | 7.5 | 0.002078 | 0.269485 |
| DIDO1 | 6 | 10.66667 | 0.001679 | 0.25494 |
| TUBB6 | 6 | 14.33333 | 0.00192 | 0.263972 |
| TIMM17A | 6 | 7 | 0.001042 | 0.23786 |
| TUBB | 6 | 13.33333 | 0.003445 | 0.285483 |
| KPNA2 | 6 | 12.66667 | 0.003538 | 0.278275 |
| MLLT4 | 6 | 13.83333 | 0.008141 | 0.284103 |
| NFE2L2 | 6 | 12.16667 | 0.006819 | 0.284409 |
| TAF7 | 6 | 6.833333 | 0.001041 | 0.248707 |
| USP4 | 6 | 17.5 | 4.41E-05 | 0.283342 |
| TAF12 | 6 | 11.5 | 0.003034 | 0.27452 |
| TAF3 | 6 | 7.833333 | 0.001013 | 0.258175 |
| OSTC | 6 | 27.66667 | 5.50E-04 | 0.272962 |
| TKT | 5 | 13.2 | 5.65E-04 | 0.273526 |
| MORF4L2 | 5 | 15.8 | 0.007554 | 0.280935 |
| LDHB | 5 | 11.8 | 0.005222 | 0.2724 |
| AP2A2 | 5 | 4.8 | 0.006839 | 0.234174 |
| TGOLN2 | 5 | 3.2 | 0.008807 | 0.201217 |
| COL4A1 | 5 | 6.6 | 0.01147 | 0.256796 |
| COX6C | 5 | 5.8 | 0.001352 | 0.21707 |
| TRIP6 | 5 | 11 | 0.00873 | 0.271421 |
| DAXX | 5 | 18.6 | 0.001247 | 0.287032 |
| ENG | 5 | 7.8 | 8.56E-04 | 0.263184 |
| RSF1 | 5 | 16 | 0.011832 | 0.296027 |
| C17orf85 | 5 | 9.4 | 7.51E-04 | 0.240564 |
| SSR4 | 5 | 10 | 0.003166 | 0.255679 |
| PDIA6 | 5 | 12.2 | 0.002863 | 0.263972 |
| CTPS1 | 5 | 10.2 | 0.00102 | 0.249293 |
| CCDC47 | 5 | 29.8 | 0 | 0.272821 |
| CD2BP2 | 5 | 14.2 | 1.26E-04 | 0.254572 |
| PAF1 | 5 | 9.4 | 1.73E-04 | 0.255186 |
| CHCHD3 | 5 | 7.4 | 3.21E-04 | 0.209174 |
| RAB8B | 5 | 7.6 | 0.002329 | 0.238826 |
| CHMP7 | 5 | 4.6 | 0.002863 | 0.231206 |
| NRIP1 | 5 | 4.4 | 6.13E-04 | 0.232527 |
| COG8 | 5 | 4.2 | 0.006129 | 0.18981 |
| RBBP6 | 5 | 8.6 | 0.003993 | 0.236055 |
| SIRT7 | 5 | 13.8 | 0.004064 | 0.278861 |
| PRPF18 | 5 | 17 | 2.39E-05 | 0.260079 |
| SEL1L | 5 | 14.6 | 1.58E-04 | 0.270588 |
| PDCD6 | 5 | 4.6 | 0.001401 | 0.243107 |
| SMG1 | 5 | 16 | 0.001735 | 0.263709 |
| NFYA | 5 | 13 | 0.010637 | 0.271282 |
| FDFT1 | 5 | 4.6 | 0.005454 | 0.224343 |
| RHOB | 5 | 12.4 | 4.89E-05 | 0.267713 |
| RNF8 | 5 | 18 | 0.002096 | 0.284715 |
| SSR2 | 5 | 7.6 | 0.00538 | 0.240893 |
| THRAP3 | 5 | 6 | 0.011511 | 0.232835 |
| SEC23A | 5 | 8.4 | 0.012013 | 0.252989 |
| POLR3E | 5 | 11.2 | 0 | 0.245134 |
| POLR3H | 5 | 11.2 | 0 | 0.245134 |
| USP15 | 5 | 20 | 9.89E-04 | 0.288913 |
| PRDX1 | 4 | 21.5 | 4.33E-04 | 0.295201 |
| ADSL | 4 | 3.75 | 0.003814 | 0.217785 |
| FASN | 4 | 9.5 | 0.003176 | 0.269074 |
| TOMM6 | 4 | 8.5 | 0 | 0.222925 |
| VCAN | 4 | 1.5 | 0.011321 | 0.136657 |
| NME4 | 4 | 6.5 | 4.15E-04 | 0.252265 |
| ATP5J2 | 4 | 7 | 8.77E-05 | 0.216981 |
| ATXN2 | 4 | 15 | 0.001088 | 0.276096 |
| BMPR2 | 4 | 15.75 | 3.72E-04 | 0.279894 |
| CHD2 | 4 | 10 | 2.19E-04 | 0.245932 |
| EIF4E2 | 4 | 11.75 | 6.58E-04 | 0.249058 |
| ZC3H11A | 4 | 9.25 | 0.004213 | 0.244455 |
| C1QBP | 4 | 14.75 | 0.002048 | 0.279008 |
| EAPP | 4 | 13 | 4.18E-06 | 0.249764 |
| FZD6 | 4 | 29.25 | 0.004122 | 0.304023 |
| TRAPPC5 | 4 | 6.25 | 0.001055 | 0.219776 |
| RABGEF1 | 4 | 20.25 | 0.007172 | 0.283039 |
| CITED2 | 4 | 7 | 7.46E-04 | 0.243891 |
| GOSR1 | 4 | 4.5 | 0.001762 | 0.188794 |
| DCAF5 | 4 | 12.25 | 0 | 0.250711 |
| EGLN1 | 4 | 19.5 | 0.004151 | 0.295201 |
| SRPK1 | 4 | 9.5 | 5.52E-05 | 0.220785 |
| LSM1 | 4 | 12.5 | 7.10E-04 | 0.254449 |
| DERL2 | 4 | 15.25 | 3.97E-05 | 0.26812 |
| ELAC2 | 4 | 6.75 | 0.007937 | 0.255186 |
| ENC1 | 4 | 5.5 | 5.25E-04 | 0.239367 |
| EXT2 | 4 | 4 | 0.020044 | 0.210505 |
| IDI1 | 4 | 4 | 0.002376 | 0.215479 |
| IP6K2 | 4 | 24.75 | 0.008066 | 0.294052 |
| GEMIN6 | 4 | 12.25 | 5.84E-05 | 0.230702 |
| TIMM23 | 4 | 7.5 | 2.07E-04 | 0.231105 |
| RRAGC | 4 | 7 | 0.00817 | 0.232018 |
| U2SURP | 4 | 12 | 2.48E-04 | 0.2298 |
| PPP2R5E | 4 | 8.5 | 0.001219 | 0.250118 |
| UBXN1 | 4 | 23.5 | 1.38E-04 | 0.286721 |
| TPM1 | 4 | 7.5 | 8.16E-05 | 0.251068 |
| TPM2 | 4 | 7.5 | 8.16E-05 | 0.251068 |
| POP5 | 4 | 13.5 | 3.66E-04 | 0.242549 |
| SUMO3 | 4 | 19.25 | 0.003002 | 0.280191 |
| ABCF1 | 3 | 12.66667 | 0.003115 | 0.258553 |
| TCF19 | 3 | 3 | 0.001007 | 0.215654 |
| TCF3 | 3 | 20.33333 | 1.22E-05 | 0.274093 |
| ARPC5 | 3 | 19.66667 | 0 | 0.278128 |
| ARPC5L | 3 | 19.66667 | 0 | 0.278128 |
| CTGF | 3 | 24.33333 | 0.001454 | 0.28641 |
| AK1 | 3 | 8 | 9.01E-04 | 0.249176 |
| PLIN3 | 3 | 3.333333 | 3.94E-04 | 0.200531 |
| S100A10 | 3 | 4 | 2.63E-05 | 0.225202 |
| CTSB | 3 | 9.333333 | 6.49E-04 | 0.253961 |
| EPN2 | 3 | 20.66667 | 0.009358 | 0.279598 |
| NECAP2 | 3 | 6 | 0 | 0.216095 |
| KDELR2 | 3 | 7 | 0.001412 | 0.229004 |
| ASXL1 | 3 | 18.66667 | 7.79E-04 | 0.274663 |
| HIST1H1C | 3 | 5.333333 | 5.07E-04 | 0.242327 |
| ATG2B | 3 | 6.333333 | 0 | 0.234278 |
| WIPI2 | 3 | 6.333333 | 0 | 0.234278 |
| UVRAG | 3 | 10.66667 | 5.55E-04 | 0.253474 |
| ATXN1L | 3 | 8.333333 | 0.001679 | 0.252025 |
| RBM17 | 3 | 7.666667 | 3.85E-04 | 0.228214 |
| B4GALT7 | 3 | 2.666667 | 0.01529 | 0.157958 |
| SEMA3A | 3 | 13 | 0.003781 | 0.258806 |
| CDK13 | 3 | 11.33333 | 3.38E-04 | 0.257672 |
| PRRC2C | 3 | 11.66667 | 6.52E-05 | 0.236689 |
| RNMT | 3 | 10.66667 | 0 | 0.224343 |
| CAMK2G | 3 | 7.333333 | 0.001061 | 0.250592 |
| IFNGR1 | 3 | 9.333333 | 0.00111 | 0.244907 |
| MARK3 | 3 | 4.333333 | 1.54E-04 | 0.228411 |
| UBL4A | 3 | 11.66667 | 0.003781 | 0.256796 |
| CANT1 | 3 | 7.666667 | 2.68E-04 | 0.251905 |
| COMMD5 | 3 | 7.666667 | 0.003781 | 0.242549 |
| HARS | 3 | 8.666667 | 0.002665 | 0.249528 |
| TSSC4 | 3 | 13.66667 | 0 | 0.249528 |
| RAB9A | 3 | 5 | 0.001113 | 0.207532 |
| VTA1 | 3 | 19.33333 | 0.00539 | 0.275952 |
| SRA1 | 3 | 5.666667 | 0 | 0.219502 |
| SPAG5 | 3 | 5 | 0.001634 | 0.232119 |
| CNPY2 | 3 | 6 | 0.004388 | 0.240455 |
| GOLGA5 | 3 | 3.666667 | 1.49E-04 | 0.15977 |
| PLOD1 | 3 | 3.333333 | 0.001887 | 0.204642 |
| LAMC1 | 3 | 13.66667 | 7.40E-04 | 0.257296 |
| PLOD2 | 3 | 3.333333 | 0.001887 | 0.204642 |
| YTHDC1 | 3 | 8.333333 | 0.001012 | 0.234798 |
| EPOR | 3 | 15 | 0 | 0.253961 |
| CSE1L | 3 | 14.66667 | 3.89E-04 | 0.255186 |
| CTBP2 | 3 | 6.333333 | 2.86E-04 | 0.235007 |
| EDC3 | 3 | 8.666667 | 7.79E-05 | 0.242438 |
| DNAJC2 | 3 | 12.33333 | 0 | 0.235111 |
| HSBP1 | 3 | 34 | 0 | 0.2875 |
| SREK1 | 3 | 19 | 0.001831 | 0.25605 |
| KLHL42 | 3 | 5.666667 | 0 | 0.232527 |
| ERO1L | 3 | 3 | 8.63E-05 | 0.214692 |
| MSMO1 | 3 | 4 | 0 | 0.183426 |
| NSDHL | 3 | 4 | 0 | 0.183426 |
| S1PR3 | 3 | 20 | 0 | 0.266499 |
| HMOX2 | 3 | 9.666667 | 0.002133 | 0.25906 |
| ING5 | 3 | 11.66667 | 1.83E-04 | 0.252145 |
| NONO | 3 | 10.33333 | 2.95E-04 | 0.257421 |
| IL1R1 | 3 | 15.66667 | 7.46E-05 | 0.257546 |
| INTS10 | 3 | 4.333333 | 4.56E-04 | 0.205437 |
| MED11 | 3 | 3.333333 | 0.001887 | 0.189131 |
| MED6 | 3 | 3.333333 | 0.001887 | 0.189131 |
| PIGO | 3 | 1.666667 | 0.666667 | 1 |
| NXT1 | 3 | 14 | 5.84E-05 | 0.230602 |
| SAR1A | 3 | 10.66667 | 0.002716 | 0.262922 |
| POP4 | 3 | 7.666667 | 0 | 0.228312 |
| RPP25L | 3 | 7.666667 | 0 | 0.228312 |
| PPP1R7 | 3 | 14.66667 | 0.002062 | 0.267984 |
| PPP2R3A | 3 | 7 | 2.18E-05 | 0.226747 |
| TFRC | 3 | 8.333333 | 0.00548 | 0.229302 |
| UXT | 3 | 1.666667 | 0.666667 | 1 |
| TAF11 | 3 | 6 | 0 | 0.217159 |
| ZNF384 | 2 | 6 | 0.003781 | 0.221061 |
| ENSP00000449026 | 2 | 18 | 0 | 0.257296 |
| TFDP1 | 2 | 22.5 | 3.67E-05 | 0.268664 |
| LAP3 | 2 | 9.5 | 1.56E-04 | 0.21322 |
| PFN2 | 2 | 36 | 0 | 0.286101 |
| MYO1E | 2 | 28 | 0 | 0.277982 |
| MYO10 | 2 | 25 | 0.003781 | 0.277982 |
| ADNP | 2 | 16 | 0 | 0.25083 |
| HEXIM1 | 2 | 10 | 0 | 0.24559 |
| AGFG1 | 2 | 7 | 0.001388 | 0.227625 |
| AHNAK2 | 2 | 5 | 0 | 0.223207 |
| AKAP13 | 2 | 15 | 0 | 0.260591 |
| SPATA13 | 2 | 18.5 | 0 | 0.260977 |
| CAPN2 | 2 | 13 | 2.22E-04 | 0.257797 |
| PCYOX1 | 2 | 10 | 0 | 0.250592 |
| CLU | 2 | 10 | 0 | 0.250592 |
| RAP2A | 2 | 1.5 | 0.003781 | 0.203775 |
| FOXK2 | 2 | 27 | 0 | 0.274235 |
| ATP1B1 | 2 | 1 | 1 | 1 |
| AVL9 | 2 | 3 | 0.003781 | 0.213737 |
| B3GALNT2 | 2 | 2.5 | 0.009979 | 0.176805 |
| GMPPB | 2 | 5 | 0.01352 | 0.212024 |
| UXS1 | 2 | 3.5 | 0.009016 | 0.175224 |
| LATS1 | 2 | 6 | 2.06E-05 | 0.237539 |
| BOD1L1 | 2 | 12.5 | 0 | 0.234278 |
| ERP29 | 2 | 9 | 5.95E-04 | 0.244116 |
| CALU | 2 | 21 | 0 | 0.263577 |
| ENTPD4 | 2 | 9.5 | 0 | 0.248707 |
| ZFYVE9 | 2 | 22 | 0 | 0.272259 |
| FAM104A | 2 | 14 | 0.003781 | 0.256672 |
| CCM2 | 2 | 16 | 0.007547 | 0.255556 |
| TRAPPC12 | 2 | 7 | 0 | 0.207532 |
| RAB13 | 2 | 9.5 | 0 | 0.207451 |
| CHMP1B | 2 | 4 | 0 | 0.218776 |
| CLASP1 | 2 | 5 | 2.03E-04 | 0.218866 |
| HTRA1 | 2 | 13 | 2.85E-05 | 0.250592 |
| LPCAT1 | 2 | 1 | 1 | 1 |
| CNOT2 | 2 | 11 | 0 | 0.239042 |
| NIF3L1 | 2 | 7.5 | 0.007547 | 0.242661 |
| COX7A2L | 2 | 6.5 | 0 | 0.184707 |
| SCAF8 | 2 | 10 | 0 | 0.205757 |
| CSDE1 | 2 | 8 | 0 | 0.236266 |
| DCUN1D3 | 2 | 9 | 6.31E-06 | 0.242772 |
| DBN1 | 2 | 2 | 0.007547 | 0.186992 |
| SPTBN1 | 2 | 1.5 | 0.003781 | 0.157675 |
| ZMYND8 | 2 | 5 | 0.011299 | 0.229501 |
| KIF3C | 2 | 10 | 0 | 0.236161 |
| TUBG1 | 2 | 8.5 | 0.007547 | 0.238934 |
| MAP1B | 2 | 15.5 | 3.66E-05 | 0.249176 |
| FAM32A | 2 | 25 | 0 | 0.259568 |
| GPATCH8 | 2 | 25 | 0 | 0.259568 |
| INHBA | 2 | 13.5 | 1.23E-04 | 0.253961 |
| GPX8 | 2 | 8 | 9.45E-04 | 0.23786 |
| EXT1 | 2 | 3 | 0 | 0.174013 |
| NDST1 | 2 | 3 | 0 | 0.174013 |
| RILPL1 | 2 | 18.5 | 0.001574 | 0.274093 |
| GCC1 | 2 | 4 | 2.10E-04 | 0.167777 |
| GLB1 | 2 | 4.5 | 0.003781 | 0.195636 |
| STX18 | 2 | 6 | 0 | 0.188323 |
| GUK1 | 2 | 10 | 0 | 0.248707 |
| HARS2 | 2 | 3 | 6.27E-05 | 0.214692 |
| HAUS6 | 2 | 1.5 | 0.003781 | 0.193066 |
| HMGB2 | 2 | 6.5 | 4.16E-04 | 0.238396 |
| SHQ1 | 2 | 42.5 | 1.01E-04 | 0.298028 |
| IFNAR1 | 2 | 6 | 1.27E-04 | 0.2302 |
| INF2 | 2 | 4.5 | 0.003781 | 0.229104 |
| ZNF609 | 2 | 5.5 | 0 | 0.20137 |
| REST | 2 | 18.5 | 0 | 0.262141 |
| LAS1L | 2 | 8.5 | 0 | 0.228608 |
| LAMTOR2 | 2 | 3 | 0 | 0.188457 |
| RRAGA | 2 | 3 | 0 | 0.188457 |
| LEPRE1 | 2 | 3 | 0 | 0.169987 |
| LTBR | 2 | 11.5 | 0 | 0.237646 |
| NFKBIE | 2 | 32.5 | 0 | 0.274805 |
| OTUD5 | 2 | 30 | 0 | 0.275521 |
| MED19 | 2 | 3 | 0 | 0.159146 |
| MXD4 | 2 | 1.5 | 0.003781 | 0.195492 |
| NAA50 | 2 | 18.5 | 0.003781 | 0.248473 |
| SCAF11 | 2 | 6.5 | 0 | 0.230702 |
| PIGM | 2 | 2.5 | 0 | 0.75 |
| PIGV | 2 | 2.5 | 0 | 0.75 |
| PIP4K2B | 2 | 14.5 | 0 | 0.247775 |
| SH2D4A | 2 | 9 | 0 | 0.228905 |
| SHOC2 | 2 | 9 | 0 | 0.224248 |
| USP47 | 2 | 9.5 | 2.35E-05 | 0.233348 |
| TBC1D15 | 2 | 10 | 0 | 0.226844 |
| STXBP1 | 2 | 7 | 0 | 0.199698 |
| VPS33B | 2 | 5.5 | 3.14E-04 | 0.190356 |
| RNF169 | 2 | 30 | 0 | 0.276674 |
| ZC3H13 | 2 | 6.5 | 2.47E-04 | 0.218324 |
| SPPL3 | 2 | 5 | 0 | 0.205837 |
| TIFA | 2 | 32 | 0 | 0.279746 |
| URI1 | 2 | 2.5 | 0 | 0.75 |
| WDR92 | 2 | 2.5 | 0 | 0.75 |
| ABCA3 | 1 | 2 | 0 | 0.181102 |
| ZDHHC16 | 1 | 14 | 0 | 0.241442 |
| CORO1C | 1 | 49 | 0 | 0.27769 |
| ADAMTS1 | 1 | 4 | 0 | 0.120255 |
| PDE8A | 1 | 4 | 0 | 0.178898 |
| TMEM30A | 1 | 18 | 0 | 0.250473 |
| BLMH | 1 | 18 | 0 | 0.250473 |
| ARHGAP17 | 1 | 30 | 0 | 0.255063 |
| ARHGAP29 | 1 | 2 | 0 | 0.169334 |
| ATMIN | 1 | 10 | 0 | 0.230401 |
| ATP1A1 | 1 | 2 | 0 | 0.666667 |
| HDLBP | 1 | 2 | 0 | 0.666667 |
| KBTBD2 | 1 | 2 | 0 | 0.176157 |
| BAG5 | 1 | 14 | 0 | 0.231611 |
| PTPN12 | 1 | 11 | 0 | 0.220877 |
| NMT1 | 1 | 8 | 0 | 0.21583 |
| BIRC6 | 1 | 8 | 0 | 0.223868 |
| CACUL1 | 1 | 13 | 0 | 0.242216 |
| CAMLG | 1 | 3 | 0 | 0.204405 |
| CCDC127 | 1 | 2 | 0 | 0.204326 |
| CCDC86 | 1 | 18 | 0 | 0.224438 |
| CCDC93 | 1 | 3 | 0 | 0.195275 |
| CDC16 | 1 | 1 | 0 | 1 |
| TBC1D20 | 1 | 1 | 0 | 1 |
| CDR2 | 1 | 31 | 0 | 0.262792 |
| CDS2 | 1 | 1 | 0 | 1 |
| LPIN3 | 1 | 1 | 0 | 1 |
| CDYL | 1 | 1 | 0 | 1 |
| ZNF644 | 1 | 1 | 0 | 1 |
| CELSR1 | 1 | 4 | 0 | 0.233245 |
| RAB32 | 1 | 10 | 0 | 0.205597 |
| CHST3 | 1 | 4 | 0 | 0.120255 |
| CISD1 | 1 | 13 | 0 | 0.237326 |
| CLPTM1L | 1 | 2 | 0 | 0.666667 |
| CNBP | 1 | 21 | 0 | 0.235845 |
| NAB1 | 1 | 3 | 0 | 0.193915 |
| COA3 | 1 | 7 | 0 | 0.179688 |
| SCO1 | 1 | 7 | 0 | 0.179688 |
| TMEM115 | 1 | 5 | 0 | 0.159578 |
| COQ6 | 1 | 1 | 0 | 1 |
| ETFDH | 1 | 1 | 0 | 1 |
| WLS | 1 | 35 | 0 | 0.266231 |
| MAGI1 | 1 | 35 | 0 | 0.266231 |
| CTSC | 1 | 1 | 0 | 1 |
| MCFD2 | 1 | 1 | 0 | 1 |
| WDR26 | 1 | 16 | 0 | 0.242772 |
| DDX19A | 1 | 31 | 0 | 0.239042 |
| DHX36 | 1 | 11 | 0 | 0.235111 |
| DNAJB9 | 1 | 13 | 0 | 0.236583 |
| DSE | 1 | 4 | 0 | 0.120255 |
| SIAH2 | 1 | 4 | 0 | 0.228017 |
| ZCCHC6 | 1 | 16 | 0 | 0.23574 |
| EXOG | 1 | 4 | 0 | 0.203383 |
| TRMT10C | 1 | 4 | 0 | 0.203383 |
| EPB41L3 | 1 | 2 | 0 | 0.136235 |
| ERC1 | 1 | 14 | 0 | 0.233348 |
| ERLIN1 | 1 | 27 | 0 | 0.256423 |
| FRYL | 1 | 1 | 0 | 1 |
| KIAA0930 | 1 | 1 | 0 | 1 |
| GBP2 | 1 | 4 | 0 | 0.227331 |
| GDF11 | 1 | 22 | 0 | 0.252145 |
| ST3GAL5 | 1 | 2 | 0 | 0.163676 |
| HAUS7 | 1 | 2 | 0 | 0.161873 |
| HECTD1 | 1 | 8 | 0 | 0.2 |
| MPHOSPH8 | 1 | 7 | 0 | 0.213824 |
| SPIRE1 | 1 | 2 | 0 | 0.186465 |
| ITPR2 | 1 | 7 | 0 | 0.222269 |
| KLF6 | 1 | 21 | 0 | 0.255063 |
| KDM3B | 1 | 16 | 0 | 0.242995 |
| PAFAH1B2 | 1 | 2 | 0 | 0.666667 |
| PAG1 | 1 | 13 | 0 | 0.242216 |
| MAFK | 1 | 6 | 0 | 0.221524 |
| 2-Mar | 1 | 5 | 0 | 0.167564 |
| MEF2BNB | 1 | 1 | 0 | 1 |
| PRPSAP1 | 1 | 1 | 0 | 1 |
| MFSD11 | 1 | 1 | 0 | 1 |
| MFSD5 | 1 | 1 | 0 | 1 |
| SPG7 | 1 | 6 | 0 | 0.221339 |
| PVR | 1 | 6 | 0 | 0.221339 |
| MLXIP | 1 | 2 | 0 | 0.163575 |
| MRFAP1L1 | 1 | 5 | 0 | 0.219411 |
| MRFAP1 | 1 | 5 | 0 | 0.219411 |
| MPPE1 | 1 | 3 | 0 | 0.6 |
| MTCH1 | 1 | 7 | 0 | 0.222269 |
| MTMR3 | 1 | 23 | 0 | 0.242105 |
| NEO1 | 1 | 2 | 0 | 0.217606 |
| NAA60 | 1 | 2 | 0 | 0.199097 |
| NADK2 | 1 | 5 | 0 | 0.218144 |
| NDUFV3 | 1 | 8 | 0 | 0.184642 |
| NPEPPS | 1 | 1 | 0 | 1 |
| TMEM43 | 1 | 1 | 0 | 1 |
| NRP2 | 1 | 3 | 0 | 0.205677 |
| SNRNP25 | 1 | 16 | 0 | 0.21636 |
| PHKA1 | 1 | 34 | 0 | 0.249176 |
| POLL | 1 | 7 | 0 | 0.205039 |
| PPP1R18 | 1 | 15 | 0 | 0.224153 |
| RALB | 1 | 23 | 0 | 0.260463 |
| ZBTB38 | 1 | 5 | 0 | 0.191044 |
| RCAN1 | 1 | 3 | 0 | 0.6 |
| STARD13 | 1 | 23 | 0 | 0.260463 |
| STRA13 | 1 | 5 | 0 | 0.22851 |
| SLC30A1 | 1 | 1 | 0 | 1 |
| SLC39A6 | 1 | 1 | 0 | 1 |
| TRAM2 | 1 | 5 | 0 | 0.1942 |
| TDP1 | 1 | 7 | 0 | 0.205039 |
| UBAC1 | 1 | 9 | 0 | 0.237433 |
| UBE2Q1 | 1 | 51 | 0 | 0.27381 |
| ZBED6 | 1 | 4 | 0 | 0.196508 |
