# Supplementary material for: Specific RNA m6A modification sites in bone marrow mesenchymal stem cells from the jawbone marrow of type 2 diabetes patients with dental implant failure
Source: Int J Oral Sci. 2023 Jan 12;15:6. doi: 10.1038/s41368-022-00202-3 (PMC9834262; doi:10.1038/s41368-022-00202-3)
Supplement: Supplementary file 6 — Supplementary Table S6 [file 41368_2022_202_MOESM6_ESM.doc]

**Table S6. Primers for specific genes used in MazF-PCR and real-time RT-PCR.**

| **Specific Genes** | **Target Sequences** |
| --- | --- |
| EPB41L3-Forward  EPB41L3-Reverse  ADNP-Forward  ADNP-Reverse  GDF11-Forward  GDF11-Reverse  RGS2-Forward  RGS2-Reverse  β-actin-Forward  β-actin-Reverse | 5’‑TGTGAACACTTGAACTTGCTAGAG-3’ 5’‑ACACGATGTCATCTCGCAACT-3’ 5’-AGTTTAAGAAAAGCCCGGAA-3’ 5’-CACTATGGACATTGCGGAAA-3’  5’-CTAGAGAGCATCAAGTCGCAG-3’  5’-CTGCCATCTGTCTGTACTGCT-3’  5’-TCCAGCGGGAGAACGATAAT-3’  5’-GCTCAAACGGGTCTTCCAATC-3’  5’-GTGGCCGAGGACTTTGATTG-3’  5’-CCTGTAACAACGCATCTCATATT-3’ |
